# Combined lifestyle factors, incident cancer, and cancer mortality: a systematic review and meta-analysis of prospective cohort studies

**DOI:** 10.1038/s41416-020-0741-x

**Published:** 2020-02-10

**Authors:** Yan-Bo Zhang, Xiong-Fei Pan, Junxiang Chen, Anlan Cao, Yu-Ge Zhang, Lu Xia, Jing Wang, Huiqi Li, Gang Liu, An Pan

**Affiliations:** 1 Department of Epidemiology and Biostatistics, School of Public Health, Tongji Medical College, Huazhong University of Science and Technology, Wuhan, Hubei China; 2 Key Laboratory of Environment and Health, Ministry of Education & Ministry of Environmental Protection, State Key Laboratory of Environmental Health (Incubating), School of Public Health, Tongji Medical College, Huazhong University of Science and Technology, Wuhan, Hubei China; 3 Department of Forensic Medicine, Tongji Medical College, Huazhong University of Science and Technology, Wuhan, Hubei China; 4 Department of Nutrition and Food Hygiene, School of Public Health, Tongji Medical College, Huazhong University of Science and Technology, Wuhan, Hubei China

**Keywords:** Cancer prevention, Risk factors, Epidemiology, Cancer epidemiology

## Abstract

**Background:**

Cancer poses a huge disease burden, which could be reduced by adopting healthy lifestyles mainly composed of healthy diet, body weight, physical activity, limited alcohol consumption, and avoidance of smoking. However, no systematic review has summarised the relations of combined lifestyle factors with cancer morbidity and mortality.

**Methods:**

EMBASE and PubMed were searched up to April 2019. Cohort studies investigating the association of combined lifestyle factors with risks of incident cancer and cancer mortality were selected. Summary hazard ratios (HRs) and 95% confidence intervals (CIs) were calculated using random-effects models. Heterogeneity and publication bias tests were conducted.

**Results:**

The HRs (95% CIs) comparing individuals with the healthiest versus the least healthy lifestyles were 0.71 (0.66–0.76; 16 studies with 1.9 million participants) for incident cancer and 0.48 (0.42–0.54; 30 studies with 1.8 million participants) for cancer mortality. Adopting the healthiest lifestyles was also associated with 17 to 58% lower risks of bladder, breast, colon, endometrial, oesophageal, kidney, liver, lung, rectal, and gastric cancer. The relations were largely consistent and significant among participants with different characteristics in the subgroup analyses.

**Conclusions:**

Adopting healthy lifestyles is associated with substantial risk reduction in cancer morbidity and mortality, and thus should be given priority for cancer prevention.

## Background

Cancer is the second leading cause of death worldwide and clearly a major growing public health issue. It was estimated that there were 18.1 million incident cancer cases and 9.6 million deaths from cancer in 2018,^1^ which contributed to 234 million all-age disability-adjusted life-years in 2017 globally.^2^ Besides, cancer costed 124.6 billion US dollars in the US and 83.2 billion Euros in the European Union annually.^3,4^ Therefore, prevention of cancer and premature deaths caused by cancer is a matter of vital importance.

Cancer is largely preventable,^5^ and adopting a healthy lifestyle (including but not limited to avoiding smoking, maintaining a healthy weight, being physically active, avoiding harmful alcohol consumption, and keeping a healthy diet) is a ‘best buy’ strategy for prevention and management of cancer, as well as for other major non-communicable diseases.^6,7^ However, to the best of our knowledge, there is a lack of randomised controlled trials testing the effects of comprehensive lifestyle interventions on prevention and prognosis of cancer. Thus, evidence from long-term cohort studies is urgently needed for clinical guidelines and public health policy-making. Previous studies have systematically reviewed the associations of individual lifestyle factors with cancer incidence^8–12^ and cancer mortality,^13–17^ and a previous systematic review summarised evidence from 12 studies about the association between the adherence to established cancer prevention guidelines for diet and physical activity and cancer outcomes;^18^ however, no systematic review and meta-analysis is currently available to investigate the combined lifestyle factors with risks of incident cancer and cancer mortality, and whether the association was consistent across participants with different characteristics remained unclear. Hence, we conducted this systematic review and meta-analysis to fill in the gap. In addition, we investigated whether the associations varied across different regions and characteristics of participants.

## Methods

### Search strategy

The study was conducted according to the Meta-analysis Of Observational Studies in Epidemiology guideline.^19^ PubMed and EMBASE were searched for studies investigating the relations of combined lifestyle factors with incident cancer and cancer mortality from database inception to April 26, 2019 by Y.-B.Z. and J.C. independently. Since the study was a part of a larger systematic review of the association of combined lifestyle with risk of mortality and major non-communicable diseases (including incident cancer, cardiovascular disease, and diabetes), the search terms included the following Medical Subject Heading terms and related exploded versions as well as keywords: “combined”, “lifestyle”, “mortality”, “cancer”, “cardiovascular disease”, “diabetes”, and “cohort study”. The detailed search strategy was published previously.^20^ No date and language restrictions were made. To identify additional publications, reference lists of relevant reviews and original studies were further searched.

### Inclusion criteria

Y.-B.Z. filtered all the citations, and another group of investigators including J.C., A.C., Y.-G.Z., L.X., J.W., and H.L. also independently performed the study selection. Discrepancies were resolved by discussion, or by conferring with the senior investigator (A.P.). There were only 62 divergences (0.08%) among 82,214 citations, mostly due to the different comprehension of the included lifestyle factors or data format issues.

Included studies should fulfil the following criteria: (1) prospective cohort studies; (2) incident total and site-specific cancer, or cancer mortality as an outcome; (3) using the combination of lifestyle factors as an exposure variable. The lifestyle factors mainly included tobacco smoking, alcohol consumption, physical activity/sedentary behaviour, overweight/obesity, diet, and sleep duration, while some studies also included hot tea consumption, un-piped water consumption, exposure to indoor air pollution, and breastfeeding. Several studies used Life’s Simple 7 (LS7) defined by the American Heart Association as the exposure which additionally included metabolic factors, i.e., blood pressure, blood glucose, and blood lipid levels.^21,22^ These studies were also included in our main analyses, because the LS7 score could reflect one’s overall lifestyle status. Accordingly, there are three major scores, defined as basic lifestyle score (which gave an equal weight to each behavioural factor, for example, most studies^23^ assigned one or zero to individuals with or without a certain healthy behaviour), the World Cancer Research Fund/American Institute for Cancer Research (WCRF/AICR) score^24,25^ (which included body weight, physical activity, consumption of plant foods, consumption of fast foods and other processed foods high in fat, starches or sugars, consumption of animal foods, consumption of sugar-sweetened drinks, alcohol consumption, and breastfeeding), and the LS7 score^21,22^ (Supplementary Table 1).

Studies were excluded if they were: (1) other publication types (such as protocols, reviews, cross-sectional studies, case-control studies, and animal experiments) or not peer-reviewed publications (such as meeting abstracts, editorials, and commentaries); (2) focusing on a single lifestyle factor or a combination of only two lifestyle factors (we assumed that lifestyles could not be entirely reflected by two factors); (3) formulation or validation of prediction models; (4) duplicate reporting from the same cohort studies or duplicate publications; (5) studies without necessary or sufficient data. Considering that cancer survivors had substantially different prevalence of risk factors and comorbidity compared with the general population, studies conducted among cancer survivors were not included in the main analysis of combined lifestyle and mortality but were pooled separately. Besides, we did not additionally select studies according to the characteristics of the participants in the main analysis, and cohorts from certain occupational groups or diseased populations were also included. Conference abstracts were not included in our analysis, but for those reporting the relations of combined lifestyle factors with incident cancer or cancer mortality, we searched online and inquired of the authors about whether the full texts had been accepted for publication to avoid missing any potentially eligible studies.

### Data extraction and quality assessment

Y.-B.Z. performed data extraction and quality assessment for all studies. Another group of investigators including J.C., A.C., Y.-G.Z., L.X., J.W., and H.L. also independently extracted data and evaluated study quality. Discrepancies were resolved by discussion, or by conferring with the senior investigator (A.P.).

The following data were extracted using standardised extraction form: first author, publication year, title, cohort name, country, study duration and mean/median follow-up duration, sample size, definitions and attainments of outcomes, definitions of included healthy lifestyle factors, and the characteristics of the participants, including sex composition, age (range and mean/median), race/ethnicity, and education level. For articles with unclear information or insufficient data, we contacted the corresponding authors at least two attempts.

We used the Newcastle-Ottawa Scale (NOS) to evaluate the study quality,^26^ which assessed the selection of the study groups (four items), the comparability of the groups (two items), and the ascertainment of outcome (three items).

### Statistical analysis

We used STATA software (Version 14.0, StataCorp, College Station, Texas, USA) to perform all meta-analyses. Hazard ratio (HR) was applied as an effect size for the pooled estimate. Risk ratios (RRs) were used in some studies and were considered as interchangeable with HRs. The odds ratios (ORs) were transformed into RRs using the following formula: RR = OR/[(1 − P_0_) + (P_0_ × OR)], where P_0_ is the incidence of the outcome in the non-exposed group.^27^ Since different studies constructed varied healthy lifestyle scores (different numbers of factors and different weights for certain factors), we pooled HR comparing the participants in the highest score group with those in the lowest score group, to represent the risk estimate comparing adopting the healthiest with the least healthy lifestyles. Most studies divided participants into three to six groups according to the distributions of the healthy lifestyle scores. Data were synthesised by random-effects models to allow heterogeneity among different studies. The weight of each study was the inverse of the sum of the within-study variance plus variance across studies.^28^ Forest plots were used to visualise the effect sizes with its 95% confidence intervals (CIs) across studies.

*I*^2^ statistic (ranging from 0 to 100%) was used to assess heterogeneity across studies, with a small value indicating less heterogeneity.^28^ Prespecified stratified analyses were performed according to the study characteristics (such as geographical region, economic level, mean/median follow-up duration, and different combinations of lifestyle factors) and population characteristics (age group, sex, race and ethnicity, and education level). To explore the sources of heterogeneity and possible effect modifications, we also tested between-group *p*-values by meta-regression.^28^

The fail-safe N statistic, Begg and Mazumdar rank correlation test, and Egger’s test were used to assess publication bias. If significant publication bias was indicated, we used the Duval and Tweedie’s trim and fill method to generate an “unbiased” estimate by adding the hypothesised studies to make the funnel plot symmetrical.^28^

## Results

### Literature search and study characteristics

**Fig. 1:**
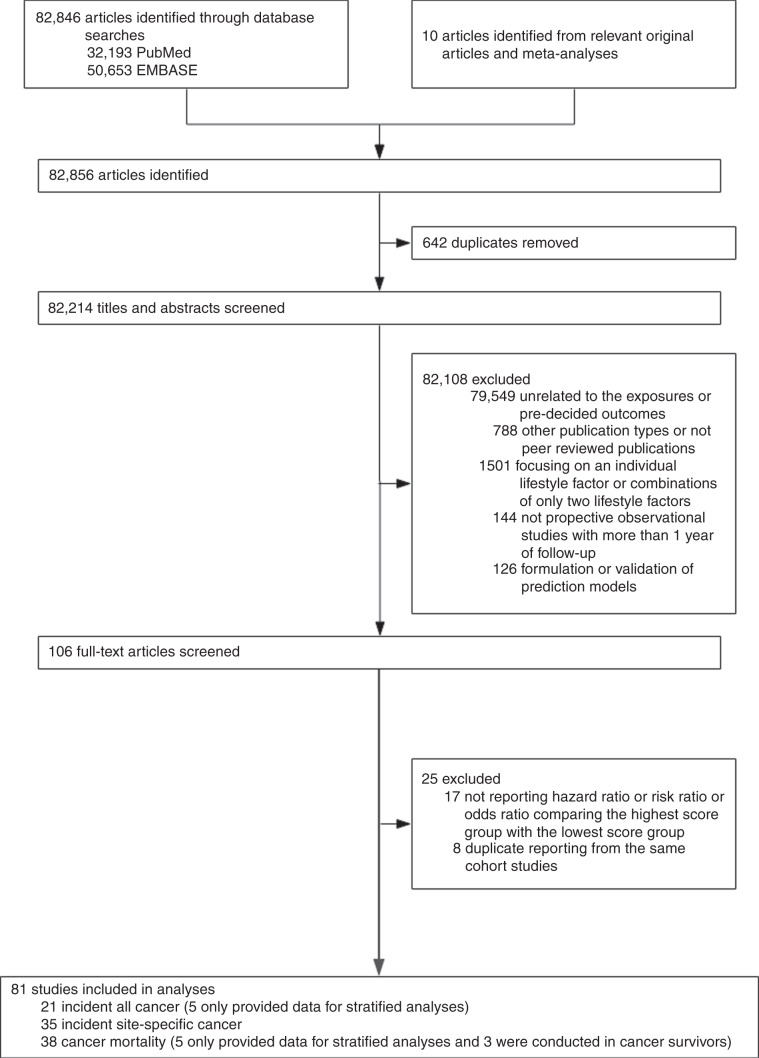
Flowchart of study selection. *HR* hazard ratio; *OR* odds ratio; RR, risk ratio. There were nine studies reporting multiple outcomes (two or more outcomes), so the total number of studies for different outcomes exceeded 81.

Based on the search strategy, 82,214 unique citations were identified, and 82,108 articles were excluded after screening for the titles and abstracts according to the inclusion/exclusion criteria. Through manual inspections of the full text, 25 studies were further excluded (the list of those publications is shown in the Supplementary Table 2). Finally, 21 studies (five studies were only used for stratified analyses) were included for meta-analyses of incident total cancer, 35 studies were included for meta-analyses of incident site-specific cancer, and 38 studies were included for meta-analyses of cancer mortality (five studies were only used for subgroup analyses and three studies were conducted in cancer survivors). Figure 1 shows the detailed study selection procedure.

The characteristics of the included studies on incident total cancer are shown in the Supplementary Table 3. Among 16 studies used for the main analysis, there were nine, three, and four studies from America, Asia, and Europe. Fourteen studies were from high-income countries. There were three and two studies being only conducted in women and men, respectively, and 11 studies reported results in men and women together (among which, seven studies also conducted subgroup analyses according to sex). The mean age at baseline ranged from less than 42.0–72.0 years old (median = 54.5, interquartile range [IQR] = 11.6). The sample size ranged from 635 to 476 396. The average follow-up duration ranged from 5.5 to over 22.2 years (median = 11.5, IQR = 4.3). The NOS scores of these studies were all equal to or greater than six (Supplementary Table 4), indicating that most studies were of high-quality.

Thirty-five studies investigated the association of combined lifestyle factors with incident site-specific cancer, including colorectal cancer (17 studies), breast cancer (16 studies), lung (8 studies), colon cancer (8 studies), prostate cancer (6 studies), rectal cancer (6 studies), ovarian cancer (6 studies), endometrial cancer (5 studies), and gastric cancer (5 studies). Several studies also investigated other cancers but with limited sample sizes (Supplementary Table 5).

The characteristics of the included studies on cancer mortality are shown in the Supplementary Table 6. Thirty studies were included in the main analysis. There were fourteen, seven, eight, and one studies from America, Asia, Europe, and Oceania, among which 27 studies were from high-income countries or regions. Three studies and two studies were conducted only in women and men, respectively (one study included in the main analysis pooled the Health Professional Follow-Up Study and the Nurses’ Health Study together and did not perform stratified analysis by sex, while another study only included participants from the Nurses’ Health Study and was additionally included in the stratified analysis by sex), and among the other 25 studies, 12 studies reported results in women and men separately. The mean baseline age ranged from 43.1 to 76.5 years old (median = 55.4, IQR = 11.1). The sample size ranged from 1,062 to 476,396. The average follow-up duration ranged from 4.0 to 33.9 years. The NOS scores of these studies were equal to or greater than five (Supplementary Table 4), indicating that most studies were of moderate- to high-quality.

### Association of combined lifestyle factors with risk of incident cancers

**Fig. 2:**
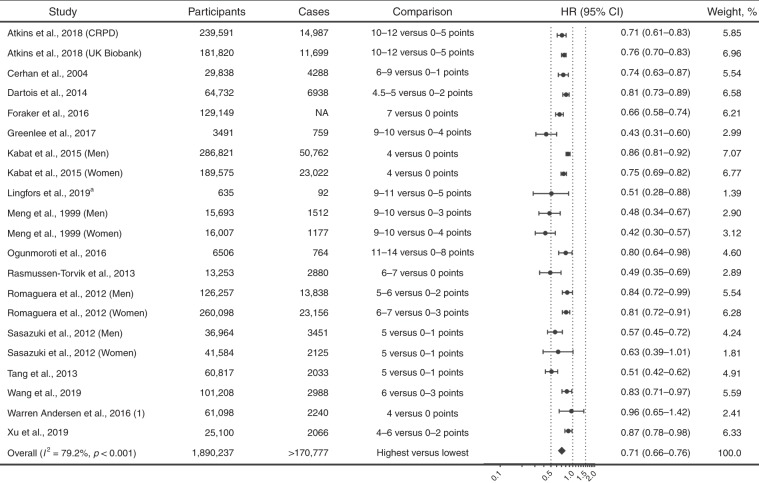
Association of combined lifestyle factors with incident cancer. *CI* confidence interval; *CRPD* Clinical Practice Research Datalink; *HR* hazard ratio; *NA* not available. The forest plot shows the HRs comparing individuals with the healthiest lifestyles (in the highest score group) with those with the least healthy lifestyles (in the lowest score group) for incident cancer. The number of participants and incident cases were shown in the figure. Each dot represents the HR for each original article, with the location of the circle representing both the direction and magnitude of the effect size, and the HR is bounded by a CI. The rhombs represent the pooled HRs. ^a^Odds ratio was reported in the study and was transformed into relative risk, which was then used in the pooled analysis.

Sixteen studies (1,890,237 participants and over 170,777 cases) reported results comparing participants with the healthiest lifestyles with those with the least healthy lifestyles for incident total cancer, and the pooled HR (95% CI) was 0.71 (0.66–0.76; *I*^*2*^ = 79.2%; Fig. 2).

**Fig. 3:**
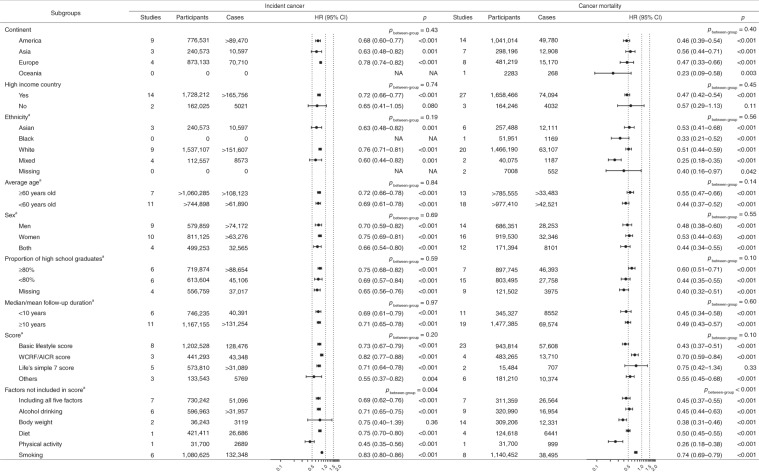
Association of combined lifestyle factors with incident cancer and cancer mortality in different subgroups. *AICR* American Institute for Cancer Research; *CI* confidence interval; *HR* hazard ratio; *NA* not available; *WCRF* World Cancer Research Fund. The forest plot shows HRs comparing individuals with the healthiest lifestyles (in the highest score group) with those with the least healthy lifestyles (in the lowest score group). Each dot represents the HR, with the location of the circle representing both the direction and magnitude of the effect size, and the HR is bounded by a CI. Foraker et al.^44^ did not report the number of incident cancer cases. ^a^Since a number of studies conducted subgroup analyses or sensitivity analyses, the total number of studies in these stratified analyses exceeded the number of studies included in main analysis.

The results were consistent in most stratified analyses (Fig. 3). However, the association was not statistically significant among individuals from non-high-income countries, although the between-group *p*-value was not statistically significant. Besides, it seemed that the association of combined lifestyle factors with incident cancer was attenuated when the lifestyle score did not include tobacco smoking (HRs [95% CI] comparing individuals with the healthiest lifestyles with those with the least healthy lifestyles were 0.69 [0.62–0.76] and 0.83 [0.80–0.86] when the lifestyle score included or did not include tobacco smoking as a component).

Begg and Mazumdar rank correlation test and Egger’s regression indicated the results might suffer from potential publication bias (Supplementary Table 7). However, the funnel plots seemed symmetrical (Supplementary Fig. 1), and the classic fail-safe N statistics indicated small possibility of publication bias.

### Association of combined lifestyle factors with risk of site-specific cancers

**Fig. 4:**
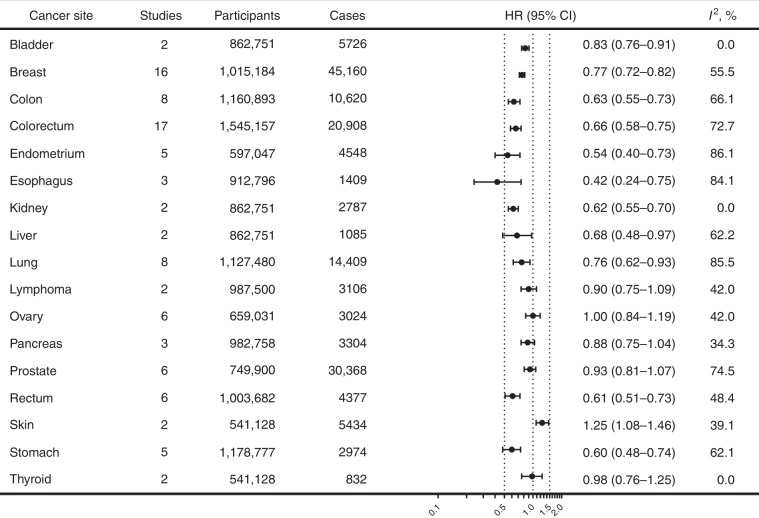
Association of combined lifestyle factors with the risks of site-specific cancers. *CI* confidence interval; *HR* hazard ratio. The forest plot shows the HRs comparing individuals with the healthiest lifestyles (in the highest score group) with those with the least healthy lifestyles (in the lowest score group) for the risks of site-specific cancers. Each dot represents the HR, with the location of the circle representing both the direction and magnitude of the effect size, and the HR is bounded by a CI.

Figure 4 showed the association of combined lifestyle factors with the risk of multiple site-specific cancers. Compared with participants with the least healthy lifestyles, those with the healthiest lifestyles were associated with 17–58% lower risks of cancer in different sites (i.e., cancer of bladder, breast, colon, colorectum, endometrium, oesophagus, kidney, liver, lung, rectum, and stomach). A positive association with skin cancer was reported (HR 1.25; 95% CI 1.08–1.46), but there were only two studies (Supplementary Fig. 2).

### Association of combined lifestyle factors with cancer mortality

**Fig. 5:**
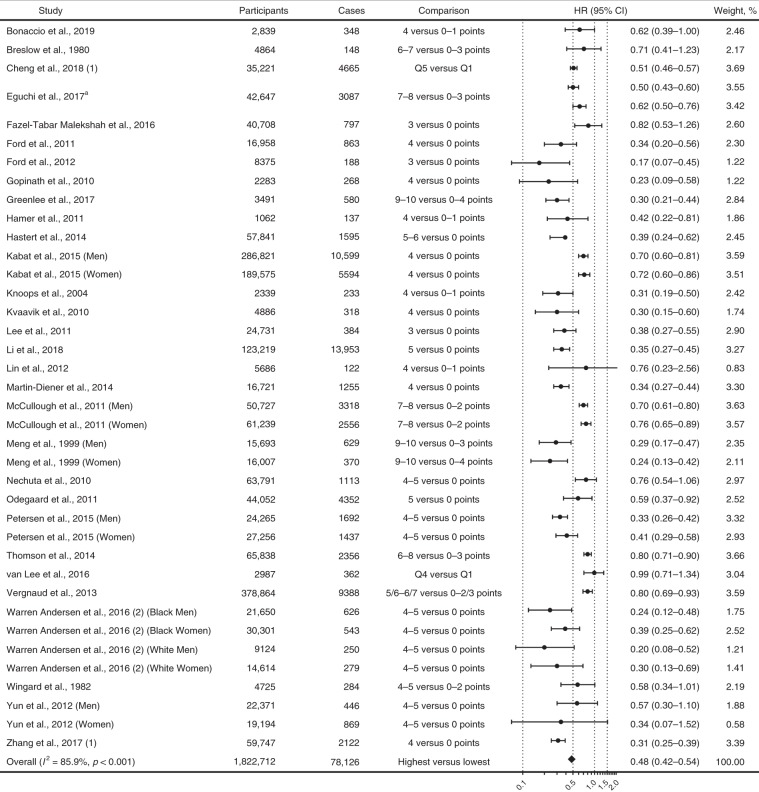
Association of combined lifestyle factors with cancer mortality. *CI* confidence interval; *CVD* cardiovascular disease; *HR* hazard ratio. The forest plot shows the HRs comparing individuals with the healthiest lifestyles (in the highest score group) with those with the least healthy lifestyles (in the lowest score group) for cancer mortality. Each dot represents the HR for each original article, with the location of the circle representing both the direction and magnitude of the effect size, and the HR is bounded by a CI. The rhombs represent the pooled HRs. ^a^Eguchi et al.^45^ reported results in well-educated and poor-educated groups respectively.

Thirty studies (1,822,712 participants and 78,126 deaths) reported results comparing participants with the healthiest lifestyles with those with the least healthy lifestyles for cancer mortality, and the pooled HR (95% CI) was 0.48 (0.42–0.54 *I*^*2*^ = 85.9%; Fig. 5).

The results were consistent in most stratified analyses (Fig. 3). However, the associations were not statistically significant among individuals from non-high-income countries, although the between-group *p*-value was not statistically significant. When evaluating different scoring systems with cancer mortality, the HRs (95% CIs) were 0.43 (0.37–0.51) for the basic lifestyle score (23 studies), 0.70 (0.59–0.84) for the WCRF/AICR score (four studies), and 0.75 (0.42–1.34) for the LS7 score (two studies). Besides, when considering all scores, the HR (95% CI) was 0.74 (0.69–0.79) when the score did not include tobacco smoking and it was 0.45 (0.37–0.55) when tobacco smoking was included in the score. Furthermore, the association seemed weaker among cancer survivors, and the HR (95% CI) was 0.70 (0.57–0.86; three studies with 6 146 patients; Supplementary Fig. 3).

Egger’s regression indicated the results might suffer from potential publication bias (Supplementary Table 7). However, the funnel plots seemed symmetrical (Supplementary Fig. 4), and the classic fail-safe N statistics along with Begg and Mazumdar rank correlation test indicated small possibility of publication bias.

## Discussion

In this systematic review and meta-analysis of prospective cohort studies, the combination of multiple healthy lifestyle factors was associated with a substantial risk reduction in incident cancer and cancer mortality. Adopting the healthiest lifestyle was associated with a 29 and 52% lower risk of incident cancer and cancer mortality compared with having the least healthy lifestyle. The associations remained largely consistent in the stratified analyses by different characteristics of studies and participants. Besides, adopting healthy lifestyles was associated with lower risks of several site-specific cancers, including bladder, breast, colon, colorectal, endometrial, oesophageal, kidney, liver, lung, rectal, and gastric cancer.

Although no meta-analysis has summarised the associations of combined lifestyle factors with cancer morbidity and mortality, the associations with each individual healthy lifestyle factor have been well established. For example, meta-analyses have shown a dose-response association between alcohol consumption and cancer risk: drinking 50 and 100 g of ethanol per day were associated with 22 and 91% higher risks of incident cancer compared with abstainers,^8^ and heavy drinkers were associated with a 31% higher risk of cancer mortality compared with non-drinkers.^14^ Body weight was also associated with several site-specific cancers: each five-unit increase in body mass index was associated with 5–50% higher risks of postmenopausal breast, colon and rectal, endometrial, oesophageal, gallbladder, kidney, liver, ovarian, pancreas, stomach cardia, and thyroid cancer, along with meningioma and multiple myeloma.^10^ In addition, men and women with obesity had 6 and 10% higher risks of cancer mortality compared with their normal-weight counterparts.^17^ For physical activity and diet, individuals in the highest group had 9%-42 and 10% lower risks of cancer,^11,12^ and 20 and 22% lower risks of cancer mortality,^13,16^ respectively, compared with those in the lowest group. Finally, tobacco smoking is the most important risk factor for cancer morbidity and mortality. Compared with never smokers, the risk of incident cancer in current smokers increased by several folds for some cancers, such as lung, laryngeal, pharyngeal, upper digestive tract, and oral cancer,^9^ furthermore, smokers had substantially increased risks for both smoking-related cancers and other cancers.^15^ Our stratified analyses showed that the associations were much stronger when smoking was included in the lifestyle scores, indicating the importance of not smoking for cancer prevention. However, 12 studies which did not include smoking in the lifestyle scores all adjusted for smoking status in the multivariate models, and the associations were still statistically significant, which indicated that other lifestyle factors except smoking were also important for cancer prevention. Taken together, encouraging the population to adopt overall healthy lifestyles is necessary for the comprehensive prevention of cancer morbidity and mortality.

On the basis of the current evidence, the World Health Organization,^29^ the American Cancer Society,^30^ the WCRF and AICR,^7^ and some other organisations^31^ have recommended general population and cancer survivors to adopt healthy lifestyles to prevent cancer and improve prognosis. Since no randomised controlled trials have provided evidence for the effects of multiple lifestyle interventions on cancer prevention and prognosis, our systematic review and meta-analysis of prospective cohort studies provided the highest-quality evidence to support these recommendations. Furthermore, our stratified analyses by different lifestyle scoring systems provided additional evidence for revisions of the current recommendations in the future. We found that the association of the WCRF/AICR score or the LS7 score with cancer mortality was weaker than the association of basic lifestyle score with cancer mortality. This is probably because avoiding smoking was not included in the WCRF/AICR recommendation or scoring system, although the WCRF/AICR acknowledged that avoiding any form of tobacco and other exposure to tobacco was the foremost means of reducing cancer risk. In addition, the weaker association with the LS7 score suggested that more emphases should be given to the upstream lifestyle factors (such as avoiding smoking, maintaining a healthy weight, being physically active, avoiding harmful alcohol consumption, and keeping a healthy diet), in addition to the intermediate metabolic changes, for the prevention of premature deaths from cancer. However, the results should also be interpreted cautiously given that the results for different score systems came from different studies with varied population characteristics, and no study has directly compared the relations of the three scoring methods with cancer mortality in the same population.

Our meta-analysis also found that adopting the healthiest lifestyles was associated with a 30% lower risk of cancer mortality among cancer survivors. However, there were only three studies on this topic. One study was conducted among 837 women with invasive breast cancer,^32^ one study among 2017 women with cancer,^33^ and the other study was conducted among 3292 patients with colorectal cancer.^34^ The mean follow-up durations were all less than 10 years, and thus more studies with longer follow-ups are still needed to investigate the relations of combined lifestyle factors with quality of life, cancer recurrence, and survival among cancer patients. Nonetheless, the limited evidence still indicated that lifestyle changes towards healthy behaviours should be recommended for patients with cancer.

We also evaluated the associations between combined lifestyles and risk of site-specific cancers. The meta-analysis found varying degrees of associations for different cancers, which may in part because of limited studies in certain cancers that precluded us from making a reliable conclusion. More importantly, the results also indicated that different cancers may have different aetiologies and some were more prone to lifestyle factors.^29^ However, it should be noted that some risk factors were not included in the lifestyle score when considering site-specific cancers, e.g., second-hand smoking and air pollution in scores for lung cancer, endogenous and exogenous oestrogen exposure history in scores for breast cancer. Intriguingly, two studies^35,36^ found individuals with the healthiest lifestyles were associated with a higher risk of skin cancer compared with those with the least healthy lifestyles. However, these two studies did not consider the sun/ultraviolet exposures on the individual level and behaviours related to the degree of sun exposure, which may confound the results, as shown in the illusive positive relation of physical activity with melanoma.^37^

The pooled estimates were stronger for the association between lifestyle factors with cancer mortality than that with incident cancer, which was also observed in original studies simultaneously reporting these two outcomes.^36,38–40^ Although the exact reasons are unknown, it is possible that lifestyle factors might have affected more aggressive cancers. In our analyses of different cancer types, we also found that the associations were stronger with more aggressive cancers (such as colorectum cancer, stomach cancer, and liver cancer) than less aggressive cancers (such as prostate cancer, thyroid cancer, lymphoma, and ovary cancer). In addition, participants with healthier lifestyles were more likely to adhere to screening guidelines, get diagnosed earlier, and have access to better treatment, which would be related to better prognosis and reduced risk of mortality.

Our stratified analyses showed that the relations of combined lifestyle factors with cancer morbidity and mortality were largely consistent among individuals from different socioeconomic backgrounds (such as different age groups, sexes, geographic regions, economic levels, races and ethnicities, and education levels). Hence, each country and region should formulate policies tailored to the preference of local population and the reality of local public health practice, in order to accelerate the progressions of achieving Sustainable Development Goal target 3.4.^41^ Notably, although most studies adjusted some of these socioeconomic factors, residual confounding in the original studies was still possible given that few studies fully adjusted for all of them.

Our study is the first systematic review and meta-analysis to quantitatively summarise the relations of combined lifestyle factors with cancer morbidity and mortality. We followed the standard procedures of the Meta-analysis Of Observational Studies in Epidemiology guideline and included 81 studies in the meta-analysis. We were able to perform various stratified analyses because of a sufficient number of included studies, and the results were largely consistent, indicating the universal importance of adopting healthy lifestyles. We were also able to identify knowledge gaps and future directions via the meta-analysis approach, and the findings of the meta-analysis are also important for establishment of public policy and clinical guidelines. Besides, the results were unlikely to be influenced by publication bias. However, several limitations should also be acknowledged. First, most studies were from high-income countries. More evidence from other countries is needed since lifestyle factors could be greatly varied across countries/regions.^42,43^ Second, different studies were conducted in populations from different socioeconomic backgrounds, and the combinations or definitions of healthy lifestyle factors varied across studies, which might generate potential heterogeneity. Hence, we only pooled the estimates comparing participants in the highest versus the lowest score group by random-effects model. Third, there is a lack of studies for several site-specific cancers, and evidence for lifestyle modifications in cancer survivors is also limited. Thus, the results should be interpreted cautiously, and more studies are needed.

In conclusion, adopting healthy lifestyles is associated with substantially lower risks of cancer morbidity and mortality. Given that the proportion of individuals having the healthiest lifestyles is low in many countries, creating an environment for better facilitating behaviour modifications should be a public health priority worldwide. More studies are still needed for site-specific cancers, and more evidence among cancer survivors and from populations in low- and middle-income countries is warranted.

## Supplementary information

Supplemental materials

## Data Availability

Data are available from Dr. An Pan (panan@hust.edu.cn) with a reasonable request.
