# Supplementary material for: Combined lifestyle factors, incident cancer, and cancer mortality: a systematic review and meta-analysis of prospective cohort studies
Source: Br J Cancer. 2020 Feb 10;122(7):1085–93. doi: 10.1038/s41416-020-0741-x (PMC7109112; doi:10.1038/s41416-020-0741-x)
Supplement: Supplementary file 1 — Supplemental materials [file 41416_2020_741_MOESM1_ESM.docx]

Supplementary Table 1 Examples of the definitions of the major lifestyle scores

| Factors | Basic lifestyle score^1^ | Life’s Simple 7 score^2^ | WCRF/AICR score^3^ |
| --- | --- | --- | --- |
| Smoking | 1 point for never smokers;  0 point for ever smokers. | 2 points for never smokers or quitting >1 year;  1 point for quitting ≤1 year;  0 point for current smokers. | Not included. |
| Alcohol drinking | For men and women respectively:  1 point for 0.1–14/7 drinks/w;  0 point for none or >14/7 drinks/w. | Not included. | For men and women respectively:  1 point for ≤2/1 drink per day;  0 point for >2/1 drink per day. |
| Physical activity | 1 point for moderate activity ≥2 h/w, or strenuous activity ≥0.5 h/w;  0 point for moderate activity <2 h/w, and strenuous activity <0.5 h/w. | 2 points for leisure–time physical activity ≥8.75 MET–h/w;  1 point for leisure–time physical activity 0.10–8.74 MET–h/w;  0 point for no leisure–time physical activity. | 1 point for moderate physical activity ≥30 minutes per day and ≥5 days per week in at least 7 years of the past 10 years;  0 point for moderate physical activity <30 minutes per day or <5 days per week or <7 years of the previous 10 years. |
| Body mass index | For participants aged <65 and ≥65 respectively:  1 point for 18.5–21.4/24.4 kg/m^2^;  0 point for <18.5 or ≥21.5/24.5 kg/m^2^. | 2 points for <25.0 kg/m^2^;  1 point for 25.0–29.9 kg/m^2^;  0 point for ≥30.0 kg/m^2^. | 1 point for 18.5–24.9 kg/m^2^;  0 point for <18.5 kg/m^2^ or ≥25.0 kg/m^2^. |
| Diet | Evaluated by the Alternate Healthy Eating Index:  1 point for the top two quintiles;  0 point for the lower three quintiles. | Evaluated by American Heart Association food guideline:  2 points for meeting 4–5 components;  1 point for meeting 2–3 components;  0 point for meeting 0–1 component. | Not included. |
| Energy density | Not included. | Not included. | 1 point for energy density <125 kcal/100g and sugar–sweetened beverage consumption <1 serving per week and fruit juice consumption ≤3 servings per week;  0 point for energy density ≥125 kcal/100g or sugar–sweetened beverage consumption ≥1 serving per week or fruit juice consumption >3 servings per week. |
| Plant food | Not included. | Not included. | 1 point for fruit and vegetable consumption ≥5 servings per day and whole grain/legume consumption ≥1 serving per day;  0 point for fruit and vegetable consumption <5 servings per day or whole grain/legume consumption <1 serving per day. |
| Animal food | Not included. | Not included. | 1 point for red meat consumption <18 ounces per week and processed meat consumption ≤1 serving per week;  0 point for red meat consumption ≥18 ounces per week or processed meat consumption >1 serving per week. |
| Blood pressure | Not included. | For systolic blood pressure and diastolic blood pressure:  2 points for SBP <120 and DBP <80 mmHg without medication;  1 point for SBP 120–139 or DBP 80–89 mmHg, or SBP <120 and DBP <80 mmHg with medication;  0 point for SBP ≥140 or DBP ≥90 mmHg. | Not included. |
| Blood glucose | Not included. | For fasting serum glucose:  2 points for <100 mg/dl (5.55 mmol/l) without medication;  1 point for 100–125 mg/dl (5.55–6.99 mmol/l) or <100 mg/dl (5.55 mmol/l) with medication;  0 point for ≥126 mg/dl (7.00 mmol/l). | Not included. |
| Blood lipid | Not included. | For blood total cholesterol:  2 points for <200 mg/dl (5.18 mmol/l) without medication;  1 point for 200–239 mg/dl (5.18–6.21 mmol/l) or <200 mg/dl (5.18 mmol/l) with medication;  0 point for ≥ 240 mg/dl (6.22 mmol/l). | Not included. |

*AICR* American Institute for Cancer Research; *DBP* diastolic blood pressure; *MET* metabolic equivalent of task; *SBP* systolic blood pressure; *WCRF* World Cancer Research Fund

The basic lifestyle scores only included behavioral factors, and the weights of included behavioral factor are identical. Those are just examples, and different studies may have varied definitions of healthy lifestyle factors for smoking, alcohol drinking, physical activity, body mass index, healthy diet; some studies may give more weight to certain variables, such as two for never smoking, one for past smoking, zero for current smoking, or two for normal weight, one for overweight and zero for obesity; some studies also included some other variables, such as sleep duration or quality, waist circumference, sedentary lifestyle, etc

Supplementary Table 2 List of studies excluded from analyses after manual inspections

| Study | Reasons for Exclusion from the Main Analyses |
| --- | --- |
| Seventeen articles not reporting HR or RR or OR comparing the highest score group with the lowest score group | |
| Brown-2018^4^ | The study used population attributable risk as statistic. |
| Catsburg-2014^5^ | The study only provided HRs for each additional healthy lifestyle factor instead of HRs comparing the healthiest lifestyle versus the least healthy lifestyle. |
| Gu-2018^6^ | The study used population attributable risk as statistic. |
| Jankovic-2017^7^ | The study only provided HRs for each additional healthy lifestyle factor instead of HRs comparing the healthiest lifestyle versus the least healthy lifestyle. |
| Kulhanova-2019^8^ | The study used population attributable risk as statistic. |
| Laaksonen-2016^9^ | The study used population attributable risk as statistic. |
| Laaksonen-2017^10^ | The study used population attributable risk as statistic. |
| Laaksonen-2018^11^ | The study used population attributable risk as statistic. |
| Menotti-2014^12^ | The study used life expectancy at 20 and 40 years old as statistic. |
| Makarem-2015^13^ | The study only provided HRs for each additional healthy lifestyle factor instead of HRs comparing the healthiest lifestyle versus the least healthy lifestyle. |
| Nöthlings-2010^14^ | The study only provided HRs for each additional healthy lifestyle factor instead of HRs comparing the healthiest lifestyle versus the least healthy lifestyle. |
| Pacheco-Figueiredo-2011^15^ | The study used adjusted prevalence of healthy lifestyle in cases and controls as statistic. |
| Platz-2000^16^ | The investigators divided the participants into 2 groups, which restricted the estimation of HRs comparing the healthiest lifestyle versus the least healthy lifestyle. |
| Vajdic-2017^17^ | The study used population attributable risk as statistic. |
| van Gemert-2015^18^ | The study used population attributable risk as statistic. |
| Yokoyama-2017^19^ | The investigators divided the participants into 2 groups, which restricted the estimation of HRs comparing the healthiest lifestyle versus the least healthy lifestyle. |
| Yun-2008^20^ | The study investigated the interaction between vegetable and fruit intake and physical activity stratified by smoking status, which restricted the estimation of the effect size of 3 combined lifestyle factors. |
| Eight duplicate reporting from the same cohort studies | |
| Assi-2018 (1)^21^ | There is a European Prospective Investigation into Cancer and Nutrition study^22^ which included the participants in this study also investigated the relationship between lifestyle and liver cancer incidence. |
| Assi-2018 (2)^23^ | There is a European Prospective Investigation into Cancer and Nutrition study^22^ which included the participants in this study also investigated the relationship between lifestyle and liver cancer incidence. |
| Charvat-2013^24^ | There is another study^25^ from Japan Public Health Center–Based Prospective Study investigating the relation of combined lifestyle factors with cancer mortality with higher Newcastle–Ottawa Scale score. |
| Erdrich-2015^26^ | The study was conducted in Nurses’ Health Study. However, there is another study^27^ investigated the relation of combined lifestyle factors with incident colorectal cancer in Nurses’ Health Study and Health Professionals Follow–up Study with longer follow–up. |
| Gramling-2010^28^ | There is another study^29^ from Women's Health Initiative Observational Study investigating the relation of combined lifestyle factors with incident breast cancer with longer follow–up duration. |
| Jiao-2009^30^ | There is another study^31^ from the American Association of Retired Persons study investigating the relation of combined lifestyle factors with incident pancreatic cancer with longer follow–up duration. |
| McKenzie-2015^32^ | The study investigated the relation of a 4–level score with incident breast cancer, whereas another study^22^ from European Prospective Investigation into Cancer and Nutrition study investigated the relation of World Cancer Research Fund score with incident breast cancer. |
| Veronese-2016^33^ | The study investigated the interaction of body weight and lifestyle and failed to report the relative risk for all–cause mortality and cause–specific mortality. Whereas other studies^34, 35^ conducted in Nurses’ Health Study and Health Professionals Follow–up Study investigated the relations of combined lifestyle factors with mortality directly. |

*HR* hazard ratio; *OR* odds ratio; *RR* risk ratio

Supplementary Table 3 Characteristics of studies related to cancer incidence

| **Author-year** | **Cohort** | **Country** | **Follow–up duration (mean or median)** | **Men (%)** | **Age (mean)** | **Ethnicity (%)^a^** | **Proportion of high school graduates (%)** | **Health status** | **Sample size** | **Outcome attainment** | **Definition of healthy lifestyles** | **NOS score** |
| --- | --- | --- | --- | --- | --- | --- | --- | --- | --- | --- | --- | --- |
| Atkins et al, 2018^36^ | Clinical Practice Research Datalink & UK Biobank | UK | 2000–2016 (6.25) | 48.83 | 60–69 (63.55) | White predominant | NA | General population | 421411 | The methods of identifying cancer cases were not reported. | Clinical Practice Research Datalink:  Smoking: 2. never smokers; 1. former smokers; 0. current smokers.  PA: 2. vigorous activity; 1. moderate activity; 0. none or mild activity.  BMI: 2. <25 kg/m^2^; 1. 25–29.99 kg/m^2^; 0. ≥30 kg/m^2^.  SBP/DBP: 2. <120 and 80 mmHg (untreated); 1. 120–139 or 80–89 mmHg or <120 and 80 mmHg (treated); 0. ≥140 or 90 mmHg.  FSG: 2. <5.6 mmol/l (not treated) or no data on FSG or diabetes; 1. 5.6–7 mmol/l (not treated) or <5.6 mmol/l (treated), or diabetes diagnosis and not treated or with no treatment information; 0. >7 mmol/l or diabetes diagnosis and treated.  TC: 2. <5.172 mmol/l (not treated) or no data on TG; 1. 5.172–6.21 mmol/l (not treated), or <5.172 mmol/l (treated), or hypercholesterolemia diagnosis and not treated or with no treatment information; 0. >6.21 mmol/l, or hypercholesterolemia diagnosis and treated.  UK Biobank:  Smoking: 2. never or quitting >12 m; 1. quitting ≤12 m; 0. current.  PA: 2. MPA ≥150 min/w, or VPA ≥75 min/w, or MVPA ≥150 min/w; 1. MPA 1–149 min/w, or VPA 1–74 min/w, or MVPA 1–149 min/w; 0. none.  BMI: 2. <25 kg/m^2^; 1. 25–29.99 kg/m^2^; 0. ≥30 kg/m^2^.  SBP/DBP: 2. <120 and 80 mmHg (untreated); 1. 120–139 or 80–89 mmHg or <120 and 80 mmHg (treated); 0. ≥140 or 90 mmHg.  FSG: 2. no self–reported prevalent diabetes and no insulin medication; 1. self–reported prevalent diabetes but no insulin medication; 0. self–reported prevalent diabetes and insulin medication.  TC: 2. no self–reported prevalent high cholesterol and no cholesterol medication; 1. self–reported prevalent high cholesterol but no cholesterol medication; 0. self–reported prevalent high cholesterol and cholesterol medication. | 7 |
| Cerhan et al, 2004^37^ | Iowa Women’s Health Study | US | 1986–1998 (11.39) | 0 | 55–69 (61.70) | White predominant | 86.10 | Postmenopausal women | 29838 | Cancer incidence was identified through the Iowa Cancer Registry. | Alcohol drinking: 1. <1 drink/d (14 g/d); 0. ≥1 drinks/d. PA: 1. exercise moderately daily and vigorously ≥1 h/w; 0. exercise moderately <once /d or vigorously <1 h/w. BMI: 1. ≤25.0 kg/m^2^; 0. > 25.0 kg/m^2^. Weight gain since age 18: 1. <11 pounds; 0. ≥11 pounds. Diet (vegetable and fruit intake excluding pulses and starchy, FFQ): 1. ≥5 servings/d; 0. <5 servings/d. Diet (complex carbohydrates intake, FFQ): 1. ≥400 g/d; 0. <400 g/d. Diet (red meat intake, FFQ): 1. <80 g/d; 0. ≥ 80g/d. Diet (consumption of fat as percentage total calories, FFQ): 1. ≤30%; 0. >30%. Diet (sodium, FFQ): 1. <2400 mg/d; 0. ≥2400 mg/d. | 7 |
| Dartois et al, 2014^38^ | *Étude Epidémiologique auprès des femmes de la Mutuelle Générale de l’Éducation Nationale* | France | 1993–2008 (12.98) | 0 | 43–68 (48.90) | White predominant | >82.20 | General population | 64732 | Invasive cancer cases (ICD-10, digestive C15–C26, lung C34, nonbasal skin C43–C44, breast C50, endometrial C54, ovarian C56, thyroid C73, hematopoietic C81–C96) were identified through questionnaires and the French National Service on Causes of Death and confirmed by physicians' confirmation. | Smoking: 1. never smoker; 0.5. former smoker; 0. current smoker. Alcohol drinking: 1. <1 drink/d (10 g/d); 0.5. 1–1.9 drink/d; 0. ≥2 drinks/d. PA: 1. ≥20 MET–h/w; 0.5. 10–19.9 MET–h/w; 0. <10 MET–h/w BMI: 1. 18.5–24.9 kg/m^2^; 0.5. 25.0–29.9 kg/m^2^; 0. ≥30 kg/m^2^; Diet (fruit and vegetable consumption): 1. ≥ 5 servings/d (400 g/d); 0.5. 3.5–4.9 servings/d; 0. <3.5 servings/d. | 8 |
| Foraker et al, 2016^39^ | Women’s Health Initiative Observational Study | US | 1993–2010 (12.70) | 0 | NA (63.50) | White 86.59  Black 8.84 | 94.92 | General population | 129149 | Cancer cases except non-melanomatous skin cancer were identified by medical records, death certificates, and autopsy reports. | Smoking: 1. Never smoking or quitting >12 m; 0. quitting ≤12 m or current smoking. PA: 1. MPA/MVPA >150 min/w or VPA >75 min/w; 0. MPA/MVPA <150 min/w and VPA <75 min/w. BMI: 1. <25 kg/m^2^; 0. ≥25 kg/m^2^. Diet (AHA, FFQ): 1. 4–5 components; 0. 0–3 components. SBP/DBP: 1. <120/80 mmHg without medication; 0. ≥120/80 mmHg or treated to <120/80 mmHg. FPG: 1. <100 mg/dl (5.55 mmol/l) without medication; 0. ≥100 mg/dl (5.55 mmol/l) or treated to <100 mg/dl (5.55 mmol/l). TC: 1. <200 mg/dl (5.18 mmol/l) without medication; 0. ≥200 mg/dl (5.18 mmol/l) or treated to <200 mg/dl (5.18 mmol/l). | 9 |
| Ford et al, 2009^b, 40^ | European Prospective Investigation into Cancer and Nutrition-Potsdam | Germany | 1994–2006 (7.80) | 38.72 | 35–65 (49.30) | White predominant | 62.40 | General population | 23153 | Cancer cases (ICD-10, C00–C43, C45–C97) were identified through self–reports and cancer registry and verified through medical records. | Smoking: 1. never smoker; 0. ever smoker. PA: 1. ≥3.5 h/w; 0. <3.5 h/w. BMI: 1. <30 kg/m^2^; 0. ≥30 kg/m^2^. Diet (summed *z* score consisting of fruits and vegetables, whole grain bread, and red meat consumption, FFQ): 1. >median; 0. ≤median. | 8 |
| Greenlee et al, 2017^2^ | Cardiovascular Health Study | US | 1989–2011 (15.00) | 38.56 | 65–98 (72.00) | White 86.71  Black 11.72 | 72.91 | General population | 3491 | Cancer cases were identified through record linkage with cancer registries. | ACS: Smoking: 2. never smokers or quitting >1 year; 1. quitting ≤1 year; 0. current smokers. Alcohol drinking (M/F): 2. non–drinker; 1. <2/1 unit/d; 0. >2/1 unit/d. PA: 2. LTPA ≥8.75 MET–h/w; 1. LTPA 0.10–8.74 MET–h/w; 0. 0 MET–h/w. BMI: 2. <25 kg/m^2^ at baseline and age 50; 1. 25–29.9 kg/m^2^ at baseline and <30 kg/m^2^ at age 50, or 25–29.9 kg/m^2^ at age 50 and <30 kg/m^2^ at baseline; 0. ≥30 kg/m^2^ at either baseline, age 50, or both. Diet (ACS, including vegetables and fruits, red and processed meats, and whole grains consumption, FFQ): 2. ≥6 score; 1. 3–5 score; 0. <3 score. AHA: Smoking: 2. never smokers or quitting >1 year; 1. quitting ≤1 year; 0. current smokers. PA: 2. LTPA ≥8.75 MET–h/w; 1. LTPA 0.10–8.74 MET–h/w; 0. 0 MET–h/w. BMI: 2. <25 kg/m^2^; 1. 25–29.9 kg/m^2^; 0. ≥30 kg/m^2^. Diet (AHA, FFQ): 2. 4–5 components; 1. 2–3 components; 0. 0–1 components. SBP/DBP: 2. <120 and 80 mmHg (untreated); 1. 120–139 or 80–89 mmHg (untreated) or <120 and 80 mmHg (treated); 0. ≥140 or 90 mmHg. FPG: 2. <100 mg/dl (5.55 mmol/l, untreated); 1. 100–125 mg/dl (5.55–6.99 mmol/l­, untreated) or <100 mg/dl (5.55 mmol/l, treated); 0. ≥126 mg/dl (7.00 mmol/l). TC: 2. <200 mg/dl (5.18 mmol/l, untreated); 1. 200–239 mg/dl (5.18–6.21 mmol/l, untreated) or <200 mg/dl (5.18 mmol/l, treated); 0. ≥ 240 mg/dl (6.22 mmol/l). | 8 |
| Harnack et al, 2002^b, 41^ | Iowa Women’s Health Study | US | 1986–1998 (NA) | 0 | 55–69 (61.70) | White predominant | 86.10 | General population | 34708 | Cancer cases including breast cancer (ICD-10, C50), colon cancer (ICD-10, C18), rectal cancer (ICD-10, C19–C20), bronchus and lung cancer (ICD-10, C34), uterus cancer (ICD-10, C54), ovary cancer (ICD-10, C56), upper digestive cancer (ICD-10, C0–C17), hematopoietic system cancer (ICD-10, C42), and lymphatic cancer cases (ICD-10, C77) were ascertained through the State Health Registry of Iowa, Iowa death certificates, active follow–up, and the National Death Index. | Alcohol drinking: 2. ≤1 drink/d; 0. >1 drink/d. PA: 2. >4 times/w; 1. 2–4 times/w; 0. <2 times/w. BMI: 2. <25.0 kg/m^2^; 1. 25.0–29.9 kg/m^2^; 0. ≥30.0 kg/m^2^. Diet (grain, FFQ): 0.4. ≥6 servings/d; 0. <6 servings/d. Diet (vegetable, FFQ): 0.4. ≥3 servings/d; 0. <3 servings/d. Diet (fruit, FFQ): 0.4. ≥2 servings/d; 0. <2 servings/d. Diet (milk, FFQ): 0.4. ≥2 servings/d; 0. <2 servings/d. Diet (meat, FFQ): 0.4. ≥2 servings/d; 0. <2 servings/d. Diet (grain food items, FFQ): 1. ≥6; 0.5. 4–5; 0. ≤3. Diet (whole grains, FFQ): 1. ≥3 servings/d; 0.5. 1–2 servings/d; 0. <1 servings/d. Diet (fruit food items, FFQ): 1. ≥7; 0.5. 5–6; 0. ≤4. Diet (vegetable food items, FFQ): 1. ≥10; 0.5. 7–9; 0. ≤6. Diet (total fat intake, FFQ): 0.67. ≤30%E; 0. >30%E. Diet (saturated fat, FFQ): 0.67. ≤10%E; 0. >10%E. Diet (cholesterol, FFQ): 0.67. ≤300 mg/d; 0. >300 mg/d. Diet (SSB, FFQ): 2. 0–1 serving/d; 1. 2–3 servings/d; 0. ≥4 servings/d. Diet (sodium, FFQ): 2. ≤2400 mg/d; 0. >2400 mg/d. | 9 |
| Kabat et al, 2015^31^ | American Association of Retired Persons | US | 1995–2008 (12.60) | 60.21 | 50–71 (62.00) | White 91.46 | 92.65 | General population | 476396 | Cancer cases were identified from cancer registries. Vital status was identified through Social Security Administration Death Master File, the National Death Index Plus, and cancer registry records, confirmed by questionnaires and other mailings. | 11–point score: Alcohol drinking (M/F): 2. 1–2/1 drinks/d; 1. nondrinkers; 0. ≥3/2 drinks/d. PA: 3. ≥5 times/w; 2. 3–4 times/w; 1. 1–2 times/w; 0. less than once /w. BMI: 3. 18.5–24.9 kg/m^2^; 2. 25.0–29.9 kg/m^2^; 1. 30.0–34.9 kg/m^2^; 0. ≥35 kg/m^2^. Diet (score consisting of fruit and vegetable, the ratio of whole grains to total grains, and red plus processed meats, FFQ): 3. 6–9 points; 2. 5 points; 1. 4 points; 0. 0–3 points. 4–point score: Alcohol drinking (M/F): 1. 1–2/1 drinks/d; 0. nondrinkers or ≥3/2 drinks/d. PA: 1. ≥5 times/w; 0. <5 times/w. BMI: 1. 18.5–24.9 kg/m^2^; 0. ≥25.0 kg/m^2^. Diet (score consisting of fruit and vegetable, the ratio of whole grains to total grains, and red plus processed meats, FFQ): 1. 6–9 points; 0. <6 points. | 8 |
| Lingfors et al, 2019^42^ | "Habo study" | Sweden | 1985–2013 (>22.23) | 100 | 33–42 (<42) | White predominant | 20.00 | General population | 635 | ICD-9 diagnoses until 1997 were 410, 411 and 431–6, and the ICD-10 diagnoses from 1997 were G45, I21–22, I61, I63–66 and FN (surgical operations of coronary arteries). Register data concerning cancer diagnosis, time of death and cause of death. Data from the register of patients treated in the hospital and the causes of mortality were also available. | Smoking: 4. not current smoker; 0. current smoker.  Alcohol drinking: 2. ≤109 g spirits/w; 0. >109 g spirits/w.  PA: 2. high level; 0. low–to–moderate level.  Diet (a weighted score consisting of consumption of vegetables, fine white bread, coarse fibre–rich bread, and visible fat, 4–item questionnaire): 3. 5–7 points; 0. 0–4 points. | 8 |
| McKenzie et al, 2016^b, 43^ | European Prospective Investigation into Cancer and Nutrition | Europe | 1992–2010 (11.74) | 30.95 | 25–70 (NA) | White predominant | <68.3 | General population | 391608 | Alcohol-related cancers (ICD-10, colorectal C18–C20, breast C50, and upper aero-digestive C01–C15 & C32), tobacco–related cancers (ICD-10, upper aero–digestive C01–C15 & C32, liver C22– C24, pancreas C25, bladder C67, kidney C64–C65, Cervix C53, stomach C16, trachea C33, lung C34, acute myeloid leukemia C92, and colorectum C18–C20), obesity–related cancers (ICD-10, esophagus C15, pancreas C25, colorectum C18–C20, breast C50, endometrium C54, kidney C64–C65, thyroid C73, and gallbladder C23), and female breast and reproductive–related cancers (ICD-10, breast C50, vulva C51, vagina C52, cervix C53, uterine C54–C55, ovary C56 and other female genital organs C57–C58) were identified through health insurance records, cancer/pathology registrations, and investigation with participants and next-of-kin. | Smoking: 4. never smokers; 3. quitting >10 years; 2. quitting ≤10 years; 1. current smoking ≤15 cigarettes/d; 0. current smoking >15 cigarettes/d. Alcohol drinking: 4. <6.0 g/d; 3. 6.0–11.9 g/d; 2. 12.0–24.9 g/d; 1. 25.0–59.9 g/d; 0. ≥60.0 g/d. PA (recreational and household PA MET): 4. 5th quintile; 3. 4th quintile; 2. 3rd quintile; 1. 2nd quintile; 0. 1st quintile. BMI: 4. <22 kg/m^2^; 3. 22–23.9 kg/m^2^; 2. 24–25.9 kg/m^2^; 1. 26–29.9 kg/m^2^; 0. ≥30 kg/m^2^. Diet (score consisting of cereal fiber, ratio of PUFA to SFA, fruits and vegetables, red and processed meat, margarine/trans–fat, and glycemic load, FFQ): 4. 5th quintile; 3. 4th quintile; 2. 3rd quintile; 1. 2nd quintile; 0. 1st quintile. | 5 |
| Meng et al, 1999^44^ | "Hawaii Department of Health survey" | US | 1975–1994 (15.61) | 49.50 | 18–NA (44.81) | White 31.06  Asian 62.61 | NA | General population | 31700 | Cancer cases were identified through the Hawaii Tumor Registry. | Smoking: 4. never smokers; 3. former smoker; 2. current smokers ≤1 ppd; 1. current smokers 1.1–1.5 ppd; 0. current smokers >1.5 ppd. Alcohol drinking (M/F): 1. 1–7/3 drinks/w; 0. none or >7/3 drinks/w. BMI: 3. 19.6–24.8 kg/m^2^; 2. <19.6 kg/m^2^ or 24.9–29.2 kg/m^2^; 1. 29.3–32.5 kg/m^2^; 0. ≥32.6 kg/m^2^. Diet (fat intake from animal products): 1. >385 g/w; 0. ≤385 g/w. Diet (fruit and vegetables consumption): 1. >1350 g/w; 0. ≤1350 g/w. | 8 |
| Ogunmoroti et al, 2016^45^ | Multi-Ethnic Study of Atherosclerosis | US | 2000–2015 (10.20) | 47.20 | 45–84 (62.00) | White 61.00  Black 28.00  Asian 11.00 | 82.30 | General population | 6506 | Malignant neoplasms (ICD-9, 140–208.92) were identified through medical records. | Smoking: 2. never smokers or quitting >12 m; 1. quitting ≤12 m; 0. current smokers.  PA: 2. MPA ≥150 min/w or VPA ≥75 min/w; 1. MPA 1–149 min/w or VPA 1–74 min/w; 0. no PA.  BMI: 2. <25 kg/m^2^; 1. 25–29.99 kg/m^2^; 0. ≥30 kg/m^2^.  Diet (AHA, FFQ): 2. 4–5 components; 1. 2–3 components; 0. 0–1 component.  SBP/DBP: 2. <120 and 80 mmHg (not treated); 1. 120–139 or 80–89 mmHg, or <120 and 80 mmHg (treated) 0. ≥140 or 90 mmHg.  FBG: 2. <100 mg/dl (5.55 mmol/l, not treated); 1. 100–125 mg/dl (5.55–6.99 mmol/l­) or <100 mg/dl (5.55 mmol/l, treated); 0. ≥126 mg/d (7.00 mmol/l).  TC: 2. <200 mg/dl (5.18 mmol/l, not treated); 1. 200–239 mg/dl (5.18–6.21 mmol/l) or <200 mg/dl (5.18 mmol/l, treated); 0. ≥240 mg/dl (6.22 mmol/l). | 6 |
| Rasmussen-Torvik et al, 2013^46^ | Atherosclerosis Risk in Communities Study | US | 1987–2006 (>13.30) | 45.50 | 45–64 (54.11) | White 74.89  Black 25.11 | 80.00 | General population | 13253 | Cancer cases (excluding nonmelanoma skin cancers) were identified through cancer registries and hospital surveillance. | Smoking: 1. never smokers or quitting >12 m; 0. quitting ≤12 m or current smokers. PA: 1. MVPA ≥150 min/w or VPA ≥75 min/w; 0. MVPA <150 min/w and VPA <75 min/w. BMI: 1. <25 kg/m^2^; 0. ≥25 kg/m^2^. Diet (AHA, FFQ): 1. 4–5 components; 0. 0–3 components. SBP/DBP: 1. <120 and 80 mmHg (untreated); 0. ≥120 or 80 mmHg, or <120/80 mmHg (treated). FSG: 1. <100 mg/dl (5.55 mmol/l, untreated); 0. ≥100 mg/dl (5.55 mmol/l) or <100 mg/dl (5.55 mmol/l, treated). TC: 1. <200 mg/dl (6.22 mmol/l, untreated); 0. ≥200 mg/dl (6.22 mmol/l) or <200 mg/dl (6.22 mmol/l, treated). | 9 |
| Romaguera et al, 2012^22^ | European Prospective Investigation into Cancer and Nutrition | Europe | 1992–NA (11.00) | 32.68 | 25–70 (52.12) | White predominant | <68.30 | General population | 386355 | Cancer cases were identified through population cancer registries, health insurance records, cancer pathology registries, and active follow-up. | Alcohol drinking (M/F): 1. ≤20/10 g/d; 0.5. 20–30/10–20 g/d; 0. >30/20 g/d. PA: 1. manual/heavy manual job, or VPA >2 h/w, or cycling/sports >30 min/d; 0.5. cycling/sports 15–30 min/d; 0. cycling/sports <15 min/d. BMI: 1. 18.5–24.9 kg/m^2^; 0.5. 25–29.9 kg/m^2^; 0. <18.5 kg/m^2^ or ≥30 kg/m^2^. Diet (energy density, FFQ): 0.5. ≤125 kcal/(100g.d); 0.25. 126–175 kcal/(100g.d); 0. ≥175 kcal/(100g.d). Diet (SSB, FFQ): 0.5. 0 g/d; 0.25. ≤250 g/d; 0. >250 g/d. Diet (fruit and vegetables, FFQ): 0.5. ≥400 g/d; 0.25. 200–399 g/d; 0. <200 g/d. Diet (DF, FFQ): 0.5. ≥25 g/d; 0.25. 12.5–24.9 g/d; 0. <12.5 g/d. Diet (red and processed meat, FFQ): 1. <500 g/w and <3 g/d; 0.5. <500 g/w and 3–49.9 g/w; 0. ≥500 g/w or ≥50 g/d. | 8 |
| Sasazuki et al, 2012^25^ | Japan Public Health Center-Based Prospective Study | Japan | 1995–2006 (9.26) | 47.06 | 45–74 (56.50) | Asian predominant | NA | General population | 78548 | Cancer cases were identified by active patient notification from major local hospitals in the study area and from data linkage with population-based cancer registries. | Smoking: 1. never smoking; 0. ever smoking. Alcohol drinking: 1. <150 g/w; 0. ≥150 g/w. PA: 1. ≥37.5 MET–h/d; 0. <37.5 MET–h/d. BMI: 1. 21–27 kg/m^2^; 0. <21 kg/m^2^ or ≥27 kg/m^2^. Diet (fish roe, FFQ): 1. <0.67 g/d; 0. ≥0.67 g/d. | 9 |
| Struijk et al, 2014^b, 47^ | European Prospective Investigation into Cancer and Nutrition-Netherlands | Netherlands | 1993–2007 (12.70) | 26.03 | 20–70 (49.13) | White predominant | 20.70 | General population | 35608 | Cancer cases were identified by annual linkage to the Dutch Cancer Registry. | Alcohol drinking (M/F): 10. ≤20/10 g/d; 0. ≥60/40 g/d. PA: 10. ≥3.5 h/w; 0. 0 h/w. Diet (vegetables, FFQ): 10. ≥200 g/d; 0. 0 g/d. Diet (fruit, FFQ): 10. ≥200 g/d; 0. 0 g/d. Diet (DF, FFQ): 10. ≥14 g/4.2MJ; 0. 0 g/4.2MJ. Diet (EPA/DHA, FFQ): 10. ≥450 mg/d; 0. 0 mg/d. Diet (SFA, FFQ): 10. <10%E; 0. ≥15%E. Diet (mono trans–FA, FFQ): 10. ≥1%E; 0. <1%E. Diet (sodium, FFQ): 10. <1.68 g/d; 0. ≥2.52 g/d. | 9 |
| Tang et al, 2013^48^ | Shanghai Men’s Health Study | China | 2002–2009 (5.50) | 100 | NA (54.80) | Asian 100 | 60.42 | General population | 60817 | Cancer cases were identified through medical records, Shanghai Cancer Registry, and the Shanghai Municipal Vital Statistics. | Smoking: 1. not a current smoker; 0. current smoker. Alcohol drinking: 1. <1 drink/d; 0. ≥1 drink/d. PA: 1. ≥54 MET–h/w; 0. <54 MET–h/w. BMI: 1. 18.5–27.9 kg/m^2^; 0. <18.5 kg/m^2^ or ≥28 kg/m^2^. Diet (fruit and vegetables, FFQ): 1. ≥451 g/d; 0. <451 g/d. | 7 |
| Thomson et al, 2014^b, 29^ | Women’s Health Initiative Observational Study | US | 1993–NA (12.60) | 0 | 50–79 (63.23) | White 88.57  Black 7.01  Asian 2.97 | >79.57 | Postmenopausal women | 65838 | Cancer cases (other than nonmelanoma skin cancer) were identified through self–reported data and verified through medical records. | Alcohol drinking: 2. nondrinker; 1. 0.1–1 drink/d; 0. >1 drink/d PA: 2. >17.5 MET–h/w; 1. 8.75–17.5 MET–h/w; 0. <8.75 MET–h/w. BMI: 2. <25 kg/m^2^ at age 18 years and baseline; 1. 25–29.9 kg/m^2^ at age 18 years and baseline; 0. ≥30 kg/m^2^ at age 18 years and baseline. Diet (score consisting of fruit and vegetable intake, total carotenoids level, whole grains%, and red and processed meat, FFQ): 2. 7–9 components; 1. 3–6 components; 0. 0–2 components. | 8 |
| Wang et al, 2019^49^ | Prediction for ASCVD Risk in China project | China | 1998–2015 (7.26) | 39.80 | 18–NA (51.72) | Asian predominant | 13.24 | General population | 101208 | Cancer cases were collected through interviewing study participants or their proxies and checking hospital records and/or death certificates to validate the diagnosis using ICD-10 (C00–C97). | Smoking: 1. <20 packs and 500 g dry tobacco during lifestyle; 0. ≥20 packs or 500 g dry tobacco during lifestyle.  Alcohol drinking (M/F): 1. ≤25/15 g/d; 0. >25/15 g/d.  PA: 1. MPA ≥150 min/w or VPA≥75 min/w; 0. MPA <150 min/w and VPA <75 min/w.  BMI: 1. 18.5–24.9 kg/m^2^; 0. <18.5 or ≥25.0 kg/m^2^.  Diet (consumption of vegetables and fruits, FFQ): 1. ≥500 g/d; 0. <500 g/d.  Diet (consumption of red meat, FFQ): 1. <50 g/d; 0. ≥50 g/d. | 9 |
| Warren Andersen et al, 2016 (1)^50^ | the Southern Community Cohort Study | US | 2002–2011 (6.00) | 40.30 | 40–79 (50.00) | White 27.00  Black 69.00 | 71.90 | General population | 61098 | Cancer cases (invasive cancers for all primary sites and in situ bladder cancers) were identified through cancer registries. | Alcohol drinking (M/F): 1. 0.1– ≤2/1 drink/d; 0. none or >2/1 drinks/d. PA: 1. MPA ≥150 min/w, or VPA ≥75 min/w, or MVPA ≥150 min/w; 0. MPA <150 min/w, and VPA <75 min/w, and MVPA <150 min/w. BMI: 1. 18.5–24.9 kg/m^2^; 0. <18.5 kg/m^2^ or ≥25.0 kg/m^2^. Diet ( score consisting of processed meat and red meat, whole grains, and vegetables and fruits, FFQ): 1. 2–3 points; 0. 0–1 point. | 7 |
| Xu et al, 2019^51^ | Alberta's Tomorrow Project | Canada | 2001–2016 (11.70) | 37.10 | 35–69 (50.50) | White predominant | >72.50 | General population | 25100 | Incident cancer was identified via linkage with the Alberta Cancer Registry. | Alcohol drinking (M/F): 1. ≤2/1 drink/d; 0. >2/1 drink/d.  PA: 1. moderate/vigorous–intensity recreational physical activity <210 min/w over the last 12 months; 0. moderate/vigorous–intensity recreational physical activity ≥210 min/w over the last 12 months.  BMI: 1. <25.0 kg/m^2^; 0. ≥25.0 kg/m^2^.  Diet (fruit and vegetables, FFQ): 1. ≥5 servings/d over the past 12 months; 0. <5 servings/d over the past 12 months.  Diet (red meat, FFQ): 1. ≥500 g/w; 0. <500 g/w.  Diet (dietary supplements, FFQ): 1. no dietary supplement over the past 12 months; 0. at least one dietary supplement over the past 12 months. | 8 |

*%E* percentage of total energy intake; *ACS* American Cancer Society; *AHA* American Heart Association; *BMI* body mass index; *DBP* diastolic blood pressure; *DF* dietary fiber; *DHA* docosahexaenoic acid; *EPA* eicosapentaenoic acid; *FA* fatty acid; *FBG* fasting blood glucose; *FFQ* food frequency questionnaire; *FPG* fasting plasma glucose; *FSG* fasting serum glucose; *ICD* International Classification of Diseases; *LTPA* leisure time physical activity; *M/F* for male and female respectively; *MET* metabolic equivalent of task; *MPA* moderate physical activity; *MVPA* moderate to vigorous physical activity; *NA* not available; *NOS* Newcastle-Ottawa Scale; *PA* physical activity; *PUFA* polyunsaturated fatty acid; *SBP* systolic blood pressure; *SFA* saturated fatty acid; *SSB* sugar-sweetened beverage; *TC* total cholesterol; *UK* the United Kingdom; *US* the United States; *VPA* vigorous physical activity

^a^The percentage of ethnic groups may not sum to 100% since some participants belonged to the other ethnic groups or did not report the information

^b^These studies were only used in stratified analyses

Supplementary Table 4 Risk of bias within studies evaluated by the Newcastle–Ottawa Scale

| Author-year | Selection of cohorts | | | | Comparability of cohorts^a^ | | | Assessment of outcome | | | NOS score |
| --- | --- | --- | --- | --- | --- | --- | --- | --- | --- | --- | --- |
|  | REC | SNEC | AE | NO | Demographic characteristic | Complications | Other factors | AO | FULE^b^ | AFUC^c^ |  |
| Aleksandrova et al, 2014^52^ | 1 | 1 | 0 | 0 | Adjusted | Not adjusted | Not adjusted | 1 | 1 | 1 | 6 |
| Artero et al, 2012^53^ | 1 | 1 | 1 | 1 | Adjusted | Not adjusted | Adjusted | 1 | 1 | 1 | 9 |
| Arthur et al, 2018^54^ | 1 | 1 | 1 | 1 | Adjusted | Adjusted | Adjusted | 1 | 1 | 1 | 9 |
| Arthur et al, 2019^55^ | 1 | 1 | 1 | 1 | Adjusted | Adjusted | Adjusted | 1 | 1 | 1 | 9 |
| Atkins et al, 2018^36^ | 1 | 1 | 1 | 1 | Adjusted | Not adjusted | Adjusted | 0 | 1 | 0 | 7 |
| Bonaccio et al, 2019^56^ | 1 | 1 | 1 | 1 | Adjusted | Adjusted | Adjusted | 1 | 1 | 1 | 9 |
| Breslow et al, 1980^57^ | 1 | 1 | 0 | 1 | Adjusted | Not adjusted | Not adjusted | 0 | 1 | 1 | 6 |
| Buckland et al, 2015^58^ | 1 | 1 | 0 | 1 | Adjusted | Not adjusted | Adjusted | 1 | 1 | 1 | 8 |
| Cerhan et al, 2004^37^ | 1 | 1 | 0 | 1 | Adjusted | Not adjusted | Not adjusted | 1 | 1 | 1 | 7 |
| Cheng et al, 2018 (1)^59^ | 1 | 1 | 0 | 1 | Adjusted | Adjusted | Adjusted | 1 | 1 | 1 | 8 |
| Cheng et al, 2018 (2)^60^ | 1 | 1 | 0 | 1 | Adjusted | Adjusted | Adjusted | 1 | 1 | 1 | 8 |
| Chang et al, 2006^61^ | 1 | 1 | 0 | 1 | Adjusted | Adjusted | Adjusted | 0 | 0 | 1 | 7 |
| Cifu et al, 2018^62^ | 1 | 1 | 0 | 1 | Adjusted | Not adjusted | Adjusted | 1 | 1 | 1 | 8 |
| Dartois et al, 2014^38^ | 1 | 1 | 0 | 1 | Adjusted | Not adjusted | Adjusted | 1 | 1 | 1 | 8 |
| Eguchi et al, 2017^63^ | 1 | 1 | 0 | 1 | Adjusted | Adjusted | Adjusted | 1 | 1 | 1 | 8 |
| Er et al, 2014^64^ | 1 | 1 | 0 | 1 | Adjusted | Not adjusted | Adjusted | 1 | 1 | 1 | 8 |
| Erdrich et al, 2015^26^ | 1 | 1 | 0 | 1 | Adjusted | Adjusted | Adjusted | 1 | 1 | 1 | 8 |
| Fazel-Tabar Malekshah et al, 2016^65^ | 1 | 1 | 1 | 1 | Adjusted | Not adjusted | Adjusted | 0 | 1 | 1 | 8 |
| Foraker et al, 2016^39^ | 1 | 1 | 1 | 1 | Adjusted | Not adjusted | Adjusted | 1 | 1 | 1 | 9 |
| Ford et al, 2009^40^ | 1 | 1 | 1 | 1 | Adjusted | Not adjusted | Not adjusted | 1 | 1 | 1 | 8 |
| Ford et al, 2011^66^ | 1 | 1 | 1 | 1 | Adjusted | Adjusted | Not adjusted | 1 | 1 | 0 | 8 |
| Ford et al, 2012^67^ | 1 | 1 | 1 | 1 | Adjusted | Adjusted | Adjusted | 1 | 1 | 0 | 8 |
| Gopinath et al, 2010^68^ | 1 | 1 | 1 | 1 | Adjusted | Adjusted | Adjusted | 1 | 1 | 0 | 8 |
| Greenlee et al, 2017^2^ | 1 | 1 | 01 | 1 | Adjusted | Adjusted | Adjusted | 0 | 1 | 1 | 8 |
| Guinter et al, 2018^69^ | 1 | 1 | 0 | 1 | Adjusted | Adjusted | Adjusted | 1 | 1 | 0 | 7 |
| Hamer et al, 2011^70^ | 1 | 1 | 1 | 1 | Adjusted | Not adjusted | Adjusted | 1 | 1 | 0 | 8 |
| Harnack et al, 2002^41^ | 1 | 1 | 1 | 1 | Adjusted | Adjusted | Adjusted | 1 | 1 | 1 | 9 |
| Harris et al, 2016^71^ | 1 | 1 | 0 | 1 | Adjusted | Adjusted | Adjusted | 1 | 1 | 0 | 8 |
| Hastert et al, 2013^72^ | 1 | 1 | 0 | 1 | Adjusted | Not adjusted | Adjusted | 1 | 1 | 1 | 8 |
| Hastert et al, 2014^73^ | 1 | 1 | 0 | 1 | Adjusted | Not adjusted | Adjusted | 1 | 1 | 1 | 8 |
| Hastert et al, 2016^3^ | 1 | 1 | 0 | 1 | Adjusted | Not adjusted | Adjusted | 1 | 1 | 1 | 8 |
| Heitz et al, 2017^74^ | 1 | 1 | 1 | 1 | Adjusted | Not adjusted | Not adjusted | 1 | 1 | 0 | 7 |
| Inoue-Choi et al, 2013^75^ | 1 | 1 | 0 | 1 | Adjusted | Adjusted | Adjusted | 1 | 1 | 1 | 8 |
| Jones et al, 2018^76^ | 1 | 1 | 0 | 1 | Adjusted | Not adjusted | Adjusted | 1 | 1 | 1 | 8 |
| Kabat et al, 2015^31^ | 1 | 1 | 0 | 1 | Adjusted | Not adjusted | Adjusted | 1 | 1 | 1 | 8 |
| Kenfield et al, 2016^77^ | 1 | 1 | 0 | 1 | Adjusted | Adjusted | Adjusted | 0 | 1 | 1 | 7 |
| Khaw et al, 2008^78^ | 1 | 1 | 1 | 1 | Adjusted | Not adjusted | Adjusted | 1 | 1 | 1 | 9 |
| Kirkegaard et al, 2010^79^ | 1 | 1 | 1 | 1 | Adjusted | Not adjusted | Adjusted | 1 | 1 | 1 | 9 |
| Kohler et al, 2018^80^ | 1 | 1 | 0 | 1 | Adjusted | Adjusted | Adjusted | 1 | 0 | 1 | 7 |
| Knoops et al, 2004^81^ | 1 | 1 | 1 | 1 | Adjusted | Not adjusted | Adjusted | 0 | 1 | 1 | 8 |
| Kvaavik et al, 2010^82^ | 1 | 1 | 1 | 1 | Adjusted | Adjusted | Adjusted | 1 | 1 | 0 | 8 |
| Lee et al, 2011^83^ | 1 | 1 | 1 | 1 | Adjusted | Not adjusted | Adjusted | 1 | 1 | 1 | 9 |
| Li et al, 2018^35^ | 1 | 1 | 0 | 1 | Adjusted | Adjusted | Adjusted | 1 | 1 | 1 | 8 |
| Lin et al, 2012^84^ | 1 | 1 | 1 | 1 | Adjusted | Adjusted | Adjusted | 1 | 0 | 0 | 7 |
| Lingfors et al, 2019^42^ | 1 | 1 | 1 | 1 | Adjusted | Not adjusted | Not adjusted | 1 | 1 | 1 | 8 |
| Lohse et al, 2016^85^ | 1 | 1 | 1 | 1 | Adjusted | Not adjusted | Adjusted | 1 | 1 | 1 | 9 |
| Martin-Diener et al, 2014^86^ | 1 | 1 | 1 | 1 | Adjusted | Not adjusted | Adjusted | 1 | 1 | 1 | 8 |
| McCullough et al, 2011^87^ | 1 | 1 | 0 | 1 | Adjusted | Not adjusted | Adjusted | 1 | 1 | 0 | 7 |
| McKenzie et al, 2016^43^ | 1 | 1 | 0 | 0 | Adjusted | Not adjusted | Adjusted | 0 | 1 | 0 | 5 |
| Meng et al, 1999^44^ | 1 | 1 | 0 | 1 | Adjusted | Not adjusted | Not adjusted | 1 | 1 | 1 | 8 |
| Navarro Silvera et al, 2006^88^ | 1 | 1 | 1 | 1 | Adjusted | Not adjusted | Adjusted | 1 | 1 | 0 | 8 |
| Nechuta et al, 2010^89^ | 1 | 1 | 1 | 1 | Adjusted | Not adjusted | Not adjusted | 1 | 1 | 1 | 8 |
| Nomura et al, 2016 (1)^90^ | 1 | 1 | 0 | 1 | Adjusted | Not adjusted | Adjusted | 0 | 1 | 1 | 7 |
| Nomura et al, 2016 (2)^91^ | 1 | 1 | 0 | 1 | Adjusted | Adjusted | Adjusted | 0 | 1 | 1 | 7 |
| Nomura et al, 2016 (3)^92^ | 1 | 1 | 0 | 1 | Adjusted | Adjusted | Adjusted | 1 | 1 | 1 | 8 |
| Odegaard et al, 2011^1^ | 1 | 1 | 0 | 1 | Adjusted | Not adjusted | Adjusted | 1 | 1 | 0 | 7 |
| Odegaard et al, 2013^93^ | 1 | 1 | 0 | 0 | Adjusted | Adjusted | Adjusted | 1 | 1 | 1 | 7 |
| Ogunmoroti et al, 2016^45^ | 1 | 1 | 1 | 0 | Adjusted | Not adjusted | Not adjusted | 1 | 1 | 0 | 6 |
| Petersen et al, 2015^94^ | 1 | 1 | 1 | 1 | Adjusted | Not adjusted | Not adjusted | 1 | 1 | 1 | 8 |
| Petimar et al, 2019^27^ | 1 | 1 | 0 | 1 | Adjusted | Adjusted | Adjusted | 1 | 1 | 1 | 8 |
| Rasmussen-Torvik et al, 2013^46^ | 1 | 1 | 1 | 1 | Adjusted | Not adjusted | Adjusted | 1 | 1 | 1 | 9 |
| Romaguera et al, 2012^22^ | 1 | 1 | 0 | 1 | Adjusted | Adjusted | Adjusted | 1 | 1 | 1 | 8 |
| Romaguera et al, 2015^95^ | 1 | 1 | 0 | 1 | Adjusted | Not adjusted | Adjusted | 0 | 0 | 1 | 6 |
| Sasazuki et al, 2012^25^ | 1 | 1 | 1 | 1 | Adjusted | Adjusted | Adjusted | 1 | 1 | 1 | 9 |
| Sheikh et al, 2019^96^ | 1 | 1 | 1 | 1 | Adjusted | Not adjusted | Not adjusted | 1 | 1 | 1 | 8 |
| Struijk et al, 2014^47^ | 1 | 1 | 1 | 1 | Adjusted | Not adjusted | Adjusted | 1 | 1 | 1 | 9 |
| Tang et al, 2013^48^ | 1 | 1 | 1 | 0 | Adjusted | Not adjusted | Adjusted | 1 | 1 | 0 | 7 |
| Thomson et al, 2014^29^ | 1 | 1 | 1 | 1 | Adjusted | Not adjusted | Adjusted | 1 | 1 | 0 | 8 |
| Van Dam et al, 2008^34^ | 1 | 1 | 0 | 1 | Adjusted | Not adjusted | Adjusted | 0 | 1 | 0 | 6 |
| van Lee et al, 2016^97^ | 1 | 1 | 1 | 1 | Adjusted | Not adjusted | Adjusted | 1 | 1 | 1 | 9 |
| Vergnaud et al, 2013^98^ | 1 | 1 | 0 | 1 | Adjusted | Not adjusted | Adjusted | 0 | 1 | 1 | 7 |
| Wang et al, 2017^99^ | 1 | 1 | 0 | 1 | Adjusted | Not adjusted | Adjusted | 1 | 1 | 0 | 7 |
| Wang et al, 2018^100^ | 1 | 1 | 0 | 1 | Adjusted | Not adjusted | Adjusted | 1 | 1 | 1 | 8 |
| Wang et al, 2019^49^ | 1 | 1 | 1 | 1 | Adjusted | Not adjusted | Adjusted | 1 | 1 | 1 | 9 |
| Warren Andersen et al, 2016 (1)^50^ | 1 | 1 | 0 | 1 | Adjusted | Not adjusted | Adjusted | 1 | 1 | 0 | 7 |
| Warren Andersen et al, 2016 (2)^101^ | 1 | 1 | 1 | 1 | Adjusted | Not adjusted | Adjusted | 1 | 0 | 0 | 7 |
| Wingard et al, 1982^102^ | 1 | 1 | 0 | 1 | Adjusted | Not adjusted | Adjusted | 1 | 0 | 1 | 7 |
| Xu et al, 2019^51^ | 1 | 1 | 0 | 1 | Adjusted | Adjusted | Adjusted | 1 | 1 | 1 | 8 |
| Yun et al, 2012^103^ | 1 | 1 | 1 | 1 | Adjusted | Not adjusted | Not adjusted | 1 | 1 | 0 | 7 |
| Zhang et al, 2017 (1)^104^ | 1 | 1 | 1 | 1 | Adjusted | Adjusted | Adjusted | 1 | 1 | 0 | 8 |
| Zhang et al, 2017 (2)^105^ | 1 | 1 | 1 | 1 | Adjusted | Not adjusted | Adjusted | 0 | 1 | 1 | 8 |
| Zhang et al, 2018^106^ | 1 | 1 | 1 | 0 | Adjusted | Adjusted | Adjusted | 1 | 1 | 1 | 8 |

*AE* ascertainment of exposure; *AFUC* adequacy of follow up of cohorts; *AO* assessment of outcome; *FULE* follow-up long enough for outcome to occur; *NO* demonstration that outcome of interest was not present at start of study; *NOS* Newcastle-Ottawa Scale; *REC* representativeness of the exposed cohort; *SNEC* selection of the non-exposed cohort

^a^To evaluate the comparability of the exposed cohort and non-exposed cohort, if the authors performed stratified analyses or adjustments for one of the following demographic characteristics, age, gender, race, marriage, education, occupation or income, then a point would be assigned to this study. If the authors performed stratified analyses or adjustments for participants’ health status or other characteristics, another point would be assigned to this study

^b^The follow-up duration was not deemed as long enough for the outcome to occur if the median or mean follow-up duration was less than five years. When the median or mean follow-up duration was not reported, the follow-up duration was not deemed as long enough for the outcome to occur if the study duration was less than ten years or not reported

^c^The follow up of a cohort was deemed as inadequate if more than 20% of the participants failed to be followed up or the study failed to report this information

Supplementary Table 5 Characteristics of studies related to incident site-specific cancers

| Author-year | Cohort | Country | Follow–up duration (mean or median) | Men (%) | Age (mean) | Ethnicity (%)^a^ | Proportion of high school graduates (%) | Health status | Sample size | | Outcome attainment | Definition of healthy lifestyles | NOS score | |
| --- | --- | --- | --- | --- | --- | --- | --- | --- | --- | --- | --- | --- | --- | --- |
| Aleksandrova et al, 2014^52^ | European Prospective Investigation into Cancer and Nutrition | Europe | 1992–2010 (12.00) | 35.00 | 25–70 (51.80) | White predominant | >23.76 | General population | 347237 | Cancer cases were identified through population cancer registries, health insurance records, cancer pathology registries, and active follow-up. | | Smoking: 1. not a current smoker; 0. current smoker. Alcohol drinking (M/F): 1. ≤24/12 g/d; 0. >24/12 g/d. PA (M/F): 1. >57/82 METs; 0. ≤57/82 METs. BMI and WC (M/F): 1. BMI <25 kg/m^2^ and WC <94/80 cm; 0. BMI ≥25 kg/m^2^ or WC ≥94/80 cm. Diet (score consisting of fruits, vegetables, red and processed meat, fiber, fish, nuts, garlic and yogurt, FFQ): 1. 5–8 points; 0. 0–4 points. | 6 | |
| Arthur et al, 2018^54^ | Canadian Study of Diet, Lifestyle, and Health | Canada | 1992–2010 (11.00) | 46.40 | <44–>75 (58.00–67.00) | NA | >98.00 | General population | 1936 for breast cancer, 2519 for endometrial cancer, and 2735 for ovarian cancer | Incident breast, endometrial, and ovarian cancer cases were ascertained via record linkage to the Canadian Cancer Registry and to the Ontario Cancer Registry. | | Smoking: 4. never smokers; 3. former smokers quitting >10 years; 2. former smokers ≤10 years; 1. current smokers ≤15 cigarettes/d; 0. current smokers >15 cigarettes/d.  Alcohol drinking: 4. <4.9 g/d; 3. 5.0–9.9 g/d; 2. 10.0–19.9 g/d; 1. 20.0–29.9 g/d; 0. ≥30.0 g/d.  PA (based on METs): 4. 5th quintile; 3. 4th quintile; 2. 3rd quintile; 1. 2nd quintile; 0. 1st quintile.  BMI: 4. <22.0 kg/m^2^; 3. 22.0–23.9 kg/m^2^; 2. 24.0–25.9 kg/m^2^; 1. 26.0–29.9 kg/m^2^; 0. ≥30.0 kg/m^2^.  Diet (score consisting of cereal fiber, red and processed meat, margarine, and fruit and vegetable intake, FFQ): 4. 5th quintile; 3. 4th quintile; 2. 3rd quintile; 1. 2nd quintile; 0. 1st quintile. | 9 | |
| Arthur et al, 2019^55^ | Women's Health Initiative study | US | 1993–2016 (17.90) | 0 | 50–79 (63.20) | White 84.20  Black 7.59  Other 7.96  Missing 0.25 | >29.72 | General population | 80123 for endometrial cancer  107183 for ovarian cancer | Primary invasive endometrial and ovarian cancer cases were collected semiannually in the clinical trials groups and annually in the Observational Study group, using in–person, mailed, or telephone questionnaires. Cancer diagnoses and tumor characteristics (histological subtype, grade, and stage) were then adjudicated centrally by trained physicians, who reviewed medical records and pathology reports. | | Smoking: 4. never smokers; 3. former smokers ≤15 pack years; 2. former smokers >15 pack years; 1. current smokers ≤15 pack years; 0. current smokers >15 pack years.  Alcohol drinking: 4. <6.0 g/day; 3. 6.0–11.9 g/day; 2. 12.0–24.9 g/day; 1. 24.0–59.9 g/day; 0. ≥60 g/day.  PA (based on METs): 4. 5th quintile; 3. 4th quintile; 2. 3rd quintile; 1. 2nd quintile; 0. 1st quintile.  BMI: 4. <25.0 kg/m2; 3. 25.0–29.9 kg/m2; 2. 30.0–34.9 kg/m2; 1. 35.0–39.9 kg/m2; 0. ≥40.0 kg/m2.  Diet (FFQ, energy–adjusted deciles of 6 dietary components, including cereal fiber, red and processed meat, the ratio of polyunsaturated to saturated fat, trans–fats, glycemic load, and fruits and vegetables, created using the residual method): 4. 5th quintile; 3. 4th quintile; 2. 3rd quintile; 1. 2nd quintile; 0. 1st quintile. | 9 | |
| Buckland et al, 2015^58^ | European Prospective Investigation into Cancer and Nutrition | Europe | 1992–2010 (11.40) | 29.80 | 25–70 (51.20) | White predominant | 45.60 | General population | 461550 | Gastric cancer cases (ICD-10, C16) were identified through cancer registries except for France, Germany, and Greece (active follow-up). And a panel of pathologists confirmed the diagnosis, classification of tumor site and morphology of the tumors for 81% of the cases. | | Smoking: 1. never smoking or quitting >10 years; 0. quitting ≤10 years or current smoking. Alcohol drinking (M/F): 1. ≤12.5/25.0 g/d; 0. >12.5/25 g/d. Diet (mMDS, FFQ or 7–d food records): 1. >8 points; 0. ≤8 points. | 8 | |
| Chang et al, 2006^61^ | Prostate, Lung, Colorectal, and Ovarian Cancer Screening Trial | US | 1993–2003 (4.90) | 0 | 55–74 (62.90) | White 92.59  Black 4.05  Asian 2.81 | 94.38 | General population | 27541 | Breast cancer cases were ascertained through self–report in the annual survey, state cancer registries, death certificates, physician reports, and next-of-kin reports. 73% of ascertained breast cancer cases were confirmed through medical record review. | | PA: 1. VPA ≥4 h/w; 0. VPA <4 h/w. BMI: 1. <30 kg/m^2^; 0. ≥ 30 kg/m^2^. Diet (energy intake, FFQ): 1. lowest quartile; 0. top 75%. | 7 | |
| Cheng et al, 2018 (2)^60^ | Iowa Women’s Health Study | US | 1986–2012 (>10.00) | 0 | 55–69 (61.70) | White predominant | 86.10 | Postmenopausal women | 35221 | Colorectal cancer diagnoses were collected through linkage with the State Health Registry of Iowa and were defined as adenocarcinoma of the colon or rectum (ICD-O-3 codes: C18.0–18.9, C19.9, and C20.9). | | Smoking: 5. never smoker; 3. former smoker; 1. current smoker.  PA: 5. VPA ≥twice/w or MPA >4 times/w; 3.VPA once/w plus MPA once/w, or MPA 2–4 times/w; 1. no VPA or MPA <twice/w.  BMI: 5. <25.0 kg/m^2^; 3. 25.0–29.9 kg/m^2^; 1. ≥30.0 kg/m^2^.  Diet (Evolutionary–concordance diet score, FFQ): 5. quintile 5; 4. quintile 4; 3. quintile 3; 2. quintile 2; 1. quintile 1. | 8 | |
| Cifu et al, 2018^62^ | National Institutes of Health-American Association of Retired Persons | US | 1995–2011 (12.50) | 0 | 50–71 (62.20) | White 91.66 | 73.02 | General population | 106126 | Incident breast cancer cases were identified by the linkage between the National Institutes of Health-American Association of Retired Persons cohort and state cancer registries. | | Smoking: 3. never smokers; 2. former smokers quitting >10 years; 1. former smokers quitting 1–10 years; 0. former smokers quitting <1 year or current smokers.  Alcohol drinking: 3. never drinking; 2. ≤14 g/d; 1. 15–28 g/d; 0. >28 g/d.  PA: 3. ≥4 h/w; 2. 1–3 h/w; 1. <1 h/w; 0. never or rarely.  BMI: 3. 18.5–24.9 kg/m^2^; 2. 15.0–29.9 kg/m^2^; 1. 20.0–34.9 kg/m^2^; 0. ≥35.0 kg/m^2^.  Diet (ACS score, FFQ): 3. 3–4 components; 2. 2 components; 1. 1 component; 0. 0 component.  TV time: 3. <1 h/d; 2. 1–4 h/d; 1. 5–6 h/d; 0. ≥7 h/d. | 8 | |
| Dartois et al, 2014^38^ | *Étude Epidémiologique auprès des femmes de la Mutuelle Générale de l’Éducation Nationale* | France | 1993–2008 (12.98) | 0 | 43–68 (48.90) | White predominant | >82.20 | General population | 64732 | Invasive cancer cases (ICD-10, digestive C15–C26, lung C34, nonbasal skin C43–C44, breast C50, endometrial C54, ovarian C56, thyroid C73, hematopoietic C81–C96) were identified through questionnaires and the French National Service on Causes of Death and confirmed by physicians' confirmation. | | Smoking: 1. never smoker; 0.5. former smoker; 0. current smoker. Alcohol drinking: 1. <1 drink/d (10 g/d); 0.5. 1–1.9 drink/d; 0. ≥2 drinks/d. PA: 1. ≥20 MET–h/w; 0.5. 10–19.9 MET–h/w; 0. <10 MET–h/w BMI: 1. 18.5–24.9 kg/m^2^; 0.5. 25.0–29.9 kg/m^2^; 0. ≥30 kg/m^2^; Diet (fruit and vegetable consumption): 1. ≥ 5 servings/d (400 g/d); 0.5. 3.5–4.9 servings/d; 0. <3.5 servings/d. | 8 | |
| Er et al, 2014^64^ | Prostate Testing for Cancer and Treatment trial | UK | 2001–2009 (NA) | 100 | 50–69 (61.65) | White >98.60 | NA | General population | 13811 | | Prostate cancer cases were histologically confirmed according to prostate-specific antigen testing. | Alcohol drinking: 1. ≤20 g/d; 0.5. 21–30 g/d; 0. >30 g/d. PA: 1. ≥7 times/w; 0.5. 3–6 times/w; 0. <3 times/wk. BMI: 0.5. lean at aged 20, 40 & trial entry; 0.25. lean at 2 time–points; 0. overweight at aged 20, 40 & trial entry. WC: 0.5. <94.0 cm; 0.25. 94.0–101.9 cm 0. ≥102.0 cm. Diet (dietary energy density, FFQ): 0.5. ≤125.0 kcal/100g/d; 0.25. 125.1–175.0 kcal/100g/d; 0. >175.0 kcal/100g/d. Diet (SSB, FFQ): 0.5. no or ≤1 fruit juice; 0.25. ≤250 g/d SSB or 1.1–2 fruit juice; 0. >250 g/d SSB or >2 fruit juice. Diet (fruit and vegetable, FFQ): 0.5. ≥400 g/d; 0.25. 200–399 g/d; 0. <200 g/d. Diet (non–starch polysaccharides, FFQ): 0.5. ≥18 g/d; 0.25. 10–17.9 g/d; 0. <10 g/d. Diet (red and processed meat): 1. <500 g/w and processed meat <3 g/d; 0.5. <500 g/w and processed meat 3–20 g/d; 0. ≥500 g/w and processed meat >20 g/d. | 8 | |
| Guinter et al, 2018^69^ | Prostate, Lung, Colorectal, and Ovarian Cancer Screening Trial | US | 1993–2009 (11.50) | 0 | 55–74 (62.00) | White 92.59  Black 4.05  Asian 2.81 | 94.38 | General population | 27153 | | Incident breast cancer cases were identified through self-report via annually mailed follow-up questionnaires, the National Death Index, physician reports, state cancer registries, and next of kin reports. (over 96% of cases were confirmed through hospital records). | Alcohol drinking: 2. 0 drink/w; 1. 0.1–7 drinks/w; 0. >7 drinks/w.  VPA: 1. >2 h/w; 0. ≤2 h/w.  BMI: 2. <25 kg/m^2^; 1. 25.0–29.9 kg/m^2^; 0. ≥30 kg/m^2^.  Diet (estrogen–related dietary pattern, FFQ): 1. <median; 0. ≥median. | 7 | |
| Harnack et al, 2002^41^ | Iowa Women’s Health Study | US | 1986–1998 (NA) | 0 | 55–69 (61.70) | White predominant | 86.10 | General population | 34708 | | Cancer cases including breast cancer (ICD-10, C50), colon cancer (ICD-10, C18), rectal cancer (ICD-10, C19–C20), bronchus and lung cancer (ICD-10, C34), uterus cancer (ICD-10, C54), ovary cancer (ICD-10, C56), upper digestive cancer (ICD-10, C0–C17), hematopoietic system cancer (ICD-10, C42), and lymphatic cancer cases (ICD-10, C77) were ascertained through the State Health Registry of Iowa, Iowa death certificates, active follow-up, and the National Death Index. | Alcohol drinking: 2. ≤1 drink/d; 0. >1 drink/d. PA: 2. >4 times/w; 1. 2–4 times/w; 0. <2 times/w. BMI: 2. <25.0 kg/m^2^; 1. 25.0–29.9 kg/m^2^; 0. ≥30.0 kg/m^2^. Diet (grain, FFQ): 0.4. ≥6 servings/d; 0. <6 servings/d. Diet (vegetable, FFQ): 0.4. ≥3 servings/d; 0. <3 servings/d. Diet (fruit, FFQ): 0.4. ≥2 servings/d; 0. <2 servings/d. Diet (milk, FFQ): 0.4. ≥2 servings/d; 0. <2 servings/d. Diet (meat, FFQ): 0.4. ≥2 servings/d; 0. <2 servings/d. Diet (grain food items, FFQ): 1. ≥6; 0.5. 4–5; 0. ≤3. Diet (whole grains, FFQ): 1. ≥3 servings/d; 0.5. 1–2 servings/d; 0. <1 servings/d. Diet (fruit food items, FFQ): 1. ≥7; 0.5. 5–6; 0. ≤4. Diet (vegetable food items, FFQ): 1. ≥10; 0.5. 7–9; 0. ≤6. Diet (total fat intake, FFQ): 0.67. ≤30%E; 0. >30%E. Diet (SFA, FFQ): 0.67. ≤10%E; 0. >10%E. Diet (cholesterol, FFQ): 0.67. ≤300 mg/d; 0. >300 mg/d. Diet (SSB, FFQ): 2. 0–1 serving/d; 1. 2–3 servings/d; 0. ≥4 servings/d. Diet (sodium, FFQ): 2. ≤2400 mg/d; 0. >2400 mg/d. | 9 | |
| Harris et al, 2016^71^ | Swedish Mammography Cohort | Sweden | 1997–2012 (NA) | 0 | 49–83 (61.52) | White predominant | >20.29 | Primarily postmenopausal women | 31514 | | Histologically confirmed incident invasive breast cancer cases were ascertained by linkage with Swedish Cancer Registers. | Alcohol drinking: 1. <10 g/d; 0. ≥10 g/d. PA: 1. walking/cycling and LTPA ≥30 min/d; 0. walking/cycling and LTPA <30 min/d BMI: 1. maintain 18.5–24.99 kg/m^2^ from age 21; 0. not maintain 18.5–24.99 kg/m^2^ from age 21. Diet (energy dense foods and soda/juice, FFQ): 1. <14 servings/w and 2 glasses/d; 0. ≥14 servings/w or 2 glasses/d. Diet (plant foods, FFQ): 1. ≥5 servings/d (400 g/d); 0. <5 servings/d. Diet (animal foods, FFQ): 1. <500 g/w; 0. ≥500 g/w. Diet (dietary supplements, FFQ): 1. not consume vitamins, minerals or other supplements on a regular basis; 0. consume vitamins, minerals or other supplements on a regular basis. | 8 | |
| Hastert et al, 2013^72^ | VITamins And Lifestyle Study | US | 2000–2008 (6.70) | 0 | 50–76 (61.30) | White >93.4 | >77.30 | Post–menopausal women | 30797 | | Incident primary breast cancers were ascertained by annual linkage with the Western Washington Surveillance, Epidemiology and End Results database (all incident cancers except for nonmelanoma skin cancers were reported from all area hospitals and offices of pathologists, oncologists, and radiotherapists). | Alcohol drinking: 1. ≤ 1 drink/d; 0. >1 drink/d.  PA: 1. MPA ≥30 min/d and ≥5 d/w; 0. MPA <30 min/d or <5 d/w.  BMI: 1. 18.5–24.9 kg/m^2^; 0. <18.5 kg/m^2^ or ≥25.0 kg/m^2^.  Diet (energy density, FFQ): 1. <125 kcal/100g and <1 SSB; 0. ≥125 kcal/100g or ≥1 SSB.  Diet (plant food, FFQ): 1. fruits and vegetables ≥5 servings/d and whole grains/legumes ≥1 serving/d; 0. fruits and vegetables <5 servings/d or whole grains/legumes <1 serving/d.  Diet (red meat, FFQ): 1. <18 oz/w; 0. ≥18 oz/w. | 8 | |
| Hastert et al, 2016^3^ | VITamins And Lifestyle Study | US | 2000–2009 (7.60) | 49.00 | 50–76 (61.10) | White >94.30  Black >1.10 | >81.30 | General population | 66920 | | Colorectal cancers (ICD-10-CM, 153–154.1) were ascertained by annual linkage with the Western Washington Surveillance, Epidemiology and End Results cancer registry. | Alcohol drinking (M/F): 1. ≤ 2/1 drink/d; 0. >2/1 drink/d.  PA: 1. MPA ≥30 min/d and ≥5 d/w in at least 7 of the past 10 years; 0. MPA <30 min/d or <5 d/w or <7 of the previous 10 years.  BMI: 1. 18.5–24.9 kg/m^2^; 0. <18.5 kg/m^2^ or ≥25.0 kg/m^2^.  Diet (energy density, FFQ): 1. <125 kcal/100g and SSB <1 /w and fruit juice ≤3 servings/w; 0. ≥125 kcal/100g or SSB ≥1 /w or fruit juice >3 servings/w.  Diet (plant food, FFQ): 1. fruits and vegetables ≥5 servings/d and whole grains/legumes ≥1 serving/d; 0. fruits and vegetables <5 servings/d or whole grains/legumes <1 serving/d.  Diet (red meat, FFQ): 1. <18 oz/w and processed meat ≤1 serving/w; 0. ≥18 oz/w or processed meat >1 serving/w. | 8 | |
| Jones et al, 2018^76^ | UK Women’s Cohort Study | UK | 1995–2014 (18.70) | 0 | 35–69 (52.00) | White 98.70 | 83.60 | General population | 30968 | | Malignant neoplasms of the colon and of the rectosigmoid junction and of the rectum (ICD-9, 153.0–154.1; ICD-10, C18–C20) were identified via record linkage of identification codes to the central register of the National Health Service Digital. | Alcohol drinking: 1. ≤10 g/d; 0.5. 10.1–20.0 g/d; 0. >20 g/d.  VPA: 1. >30 min/d; 0.5. 15–30 min/d; 0. <15 min/d.  BMI: 1. 18.5–24.9 kg/m^2^; 0.5 25.0–29.9 kg/m^2^; 0. <18.5 or ≥30.0 kg/m^2^.  Diet (energy density, FFQ): 0.5. ≤125 kcal/100g/d; 0.25 126–175 kcal/100g/d; 0. >175 kcal/100g/d.  Diet (SSB, FFQ): 0.5. 0 g/d; 0.25. ≤250 g/d; 0. >250 g/d.  Diet (fruit and vegetable, FFQ): 0.5. ≥400 g/d; 0.25. 200–399 g/d; 0. <200 g/d.  Diet (DF, FFQ): 0.5. ≥25 g/d; 0.25. 12.5–24.9 g/d; 0. <12.5 g/d.  Diet (red meat and processed meat, FFQ): 1. <500 g/d and <3 g/d; 0.5. <500 g/d and 3–49 g/d; 0. ≥500 g/d or ≥50 g/d.  Diet (sodium, FFQ): 1. ≤1.5 g/d; 0.5. 1.6–2.4 g/d; 0. >2.4 g/d.  Breast feeding: 1. ≥6 m; 0.5 0.1–5.9 m; 0. no | 8 | |
| Kabat et al, 2015^31^ | American Association of Retired Persons | US | 1995–2008 (12.60) | 60.21 | 50–71 (62.00) | White 91.46 | 92.65 | General population | 476396 | | Cancer cases were identified from cancer registries. Vital status was identified through Social Security Administration Death Master File, the National Death Index Plus, and cancer registry records, confirmed by questionnaires and other mailings. | 11–point score: Alcohol drinking (M/F): 2. 1–2/1 drinks/d; 1. nondrinkers; 0. ≥3/2 drinks/d. PA: 3. ≥5 times/w; 2. 3–4 times/w; 1. 1–2 times/w; 0. less than once /w. BMI: 3. 18.5–24.9 kg/m^2^; 2. 25.0–29.9 kg/m^2^; 1. 30.0–34.9 kg/m^2^; 0. ≥35 kg/m^2^. Diet (score consisting of fruit and vegetable, the ratio of whole grains to total grains, and red plus processed meats, FFQ): 3. 6–9 points; 2. 5 points; 1. 4 points; 0. 0–3 points. 4–point score: Alcohol drinking (M/F): 1. 1–2/1 drinks/d; 0. nondrinkers or ≥3/2 drinks/d. PA: 1. ≥5 times/w; 0. <5 times/w. BMI: 1. 18.5–24.9 kg/m^2^; 0. ≥25.0 kg/m^2^. Diet (score consisting of fruit and vegetable, the ratio of whole grains to total grains, and red plus processed meats, FFQ): 1. 6–9 points; 0. <6 points. | 8 | |
| Kenfield et al, 2016^77^ | Health Professionals Follow-up Study & Physicians’ Health Study | US | 1987–2010 (11.81) | 100 | 40–84 (55.82) | White predominant | Predominant | General population | 63025 | | Incident prostate cancer cases were self–reported and confirmed through medical record and pathology report. Prostate cancer deaths were determined through death certificates, medical record, and next of kin. Lethal prostate cancer cases were metastases or death from prostate cancer. | Smoking 1. never smokers or quitting ≥10 years; 0. current smokers or quitting <10 years. PA: for women, 1. VPA ≥3 h/w or brisk walking ≥7 h/w; 0. VPA <3 h/w and brisk walking <7 h/w; for men, 1. exercise to sweat ≥2–3 times/w; 0. exercise to sweat <2–3 times/w. BMI: 1. <30 kg/m^2^; 0. ≥30 kg/m^2^. Diet (tomatoes including raw tomatoes, tomato juice, tomato sauce, salsa and pizza for women, whereas tomatoes and tomato juice for men, FFQ): for women, 1. ≥7 servings/w; 0. <7 servings/w; for men, 1. ≥4 servings/w; 0. <4 servings/w. Diet (fatty fish including mackerel, salmon, sardines, bluefish and swordfish for women, whereas dark fish for men, FFQ): 1. ≥1 serving/w; 0. <1 serving/w. Diet (processed meat including beef or pork hot dogs, bacon, salami, bologna, or other processed meat sandwiches and other processed meats for women, FFQ): for women, 1. <3 servings/w; 0. ≥3 servings/w; for men, 1. hot dogs <1 serving/w or hotdogs, bacon, and processed meats <3 servings/w; 0. hot dogs ≥1 serving/w or hotdogs, bacon, and processed meats ≥3 servings/w. | 7 | |
| Kirkegaard et al, 2010^79^ | Diet, Cancer and Health Cohort Study | Denmark | 1993–2006 (9.90) | 48.00 | 50–64 (56.00) | White predominant | <21.00 | General population | 55487 | | Colorectal cancer cases were identified through the Danish Cancer Registry and Danish Pathology Databank. | Smoking: 1. not a current smoker; 0. current smoker. Alcohol drinking (M/F): 1. ≤14/7 drinks/w; 0. >14/7 drinks/w. PA: 1. ≥30 min/d, or had a job with manual activity; 0. <30 min/d, and had a job with manual activity. WC (M/F): 1. <102/88 cm; 0. ≥102/88 cm. Diet (score consisting of fruit and vegetables, red and processed meat, DF, and fat consumption, FFQ): 1. 4 points; 0. 0–3 points. | 9 | |
| Kohler et al, 2018^80^ | Wheat Bran Fiber and Ursodeoxycholic Acid trials | US | 1990–NA (3.10–3.20) | 66.95 | 40–80 (65.85) | White 94.80 | >34.87 | Patients who had at least one adenoma removed via a colonoscopy. | 1357 | | Medical records and pathology reports were used to collect recurrent adenoma characteristics such as number, size, location, and histology. | Alcohol drinking (M/F): 2. non–drinker; 1. 1–2/1 drink/d; 0. ≥3/2 drinks/d.  PA: 2. >17.5 MET h/w; 1. 8.75–17.5 MET h/w; 0. <8.75 MET h/w.  BMI: 2. 18.5–25.0 kg/m^2^; 1. 25.1–30.0 kg/m^2^; 0. >30.0 kg/m^2^.  Diet (a 6–point score consisting of consumption of fruit & vegetables, total carotenoids, and red & processed meat, FFQ): 2. 5–6 sub–score sum; 1. 2–4 sub–score sum; 0. 0–1 sub–score sum. | 7 | |
| Navarro Silvera et al, 2006^88^ | Canadian National Breast Screening Study | Canada | 1980–2000 (16.40) | 0 | 40–59 (48.54) | NA | 54.95 | General population | 40318 | | Breast cancer cases were identified through cancer databases. | PA: 1. any VPA; 0. no VPA. BMI: 1. <25 kg/m^2^; 0. ≥25 kg/m^2^. Diet (energy intake, FFQ): 1. <1972 kcal/d; 0. ≥1972 kcal/d. | 8 | |
| Nomura et al, 2016 (1)^90^ | Black Women’s Health Study | US | 1995–2011 (13.86) | 0 | 21–69 (38.23) | Black 100 | 82.10 | General population | 42792 | | Breast cancer cases were ascertained through self-report and cancers registries. Hospital or registry pathology data were obtained for >85% of cases, among which 99.4% were confirmed. | Alcohol drinking: 1. <7 servings/w; 0.5. 7–13 servings/w; 0. ≥14 servings/w. PA: 1. VPA 3–4 h/w or walking for exercise 5–6 h/w and <8 h/d sitting; 0.5. VPA 3–4 h/w or walking for exercise 5–6 h/w and ≥8 h/d sitting, or VPA 1–2 h/w or walking for exercise 1–4 h/w and <8 h/d sitting, or VPA or walking for exercise <1 h/w and ≤5 h/d sitting; 0. VPA 1–2 h/w or walking for exercise 1–4 h/w and ≥8 h/d sitting, or VPA or walking for exercise <1 h/w and >5 h/d sitting. BMI: 1. 18.5–24.9 kg/m^2^ and weight gain ≤6.8 kg; 0.5. 25.0–29.9 kg/m^2^ or weight gain 6.81–13.61 kg; 0. ≥30 kg/m^2^ and weight gain ≥13.62 kg. Diet (SSB, FFQ): 1. 0 g/d; 0.5. <250 g/d; 0. ≥250 g/d. Diet (fruit and vegetables, FFQ): 1. ≥5 servings/d and DF ≥25 g/d; 0.5. 3–4.9 servings/d and DF 12.5–24.9 g/d; 0. <3 servings/d and DF <12.5 g/d. Diet (red/processed meat, FFQ): 1. <500 g/w and <3 g/w; 0.5. <500 g/w and 3–49.9 g/w; 0. ≥500 g/w and ≥50 g/w. Diet (sodium, FFQ): 1. ≤1500 mg/d; 0.5. 1501–2400 mg/d; 0. >2400 mg/d. | 7 | |
| Nomura et al, 2016 (2)^91^ | Black Women’s Health Study | US | 1995–2013 (13.86) | 0 | 21–69 (38.23) | Black 100 | 82.20 | General population | 42792 | | Colon and rectal cancer cases (ICD-10, colon cancer C18.0–C18.9; rectal cancer C19.9 and C20.9) were ascertained through self-report and cancers registries. Hospital or registry pathology data were obtained for >85% of cases, among which 99.4% were confirmed. | Alcohol drinking: 1. <7 servings/w; 0.5. 7–13 servings/w; 0. ≥14 servings/w. PA: 1. VPA 3–4 h/w or walking for exercise 5–6 h/w and <8 h/d sitting; 0.5. VPA 3–4 h/w or walking for exercise 5–6 h/w and ≥8 h/d sitting, or VPA 1–2 h/w or walking for exercise 1–4 h/w and <8 h/d sitting, or VPA or walking for exercise <1 h/w and ≤5 h/d sitting; 0. VPA 1–2 h/w or walking for exercise 1–4 h/w and ≥8 h/d sitting, or VPA or walking for exercise <1 h/w and >5 h/d sitting. BMI: 1. 18.5–24.9 kg/m^2^ and weight gain ≤6.8 kg; 0.5. 25.0–29.9 kg/m^2^ or weight gain 6.81–13.61 kg; 0. ≥30 kg/m^2^ and weight gain ≥13.62 kg. Diet (SSB, FFQ): 1. 0 g/d; 0.5. <250 g/d; 0. ≥250 g/d. Diet (fruit and vegetables, FFQ): 1. ≥5 servings/d and DF ≥25 g/d; 0.5. 3–4.9 servings/d and DF 12.5–24.9 g/d; 0. <3 servings/d and DF <12.5 g/d. Diet (red/processed meat, FFQ): 1. <500 g/w and <3 g/w; 0.5. <500 g/w and 3–49.9 g/w; 0. ≥500 g/w and ≥50 g/w. Diet (sodium, FFQ): 1. ≤1500 mg/d; 0.5. 1501–2400 mg/d; 0. >2400 mg/d. | 7 | |
| Nomura et al, 2016 (3)^92^ | Iowa Women’s Health Study | US | 1986–2010 (NA) | 0 | 55–69 (61.70) | White predominant | 80.70 | Postmenopausal women | 36626 | | Breast cancer cases were identified through annual linkage with the State Health Registry of Iowa. | Alcohol drinking: 1. ≤10 g/d; 0.5. 10.1–20 g/d; 0. >20 g/d. PA: 1. VPA ≥2 times/w or MPA ≥5 times/w; 0.5. MPA 2–4 times/w or MVPA once /w; 0. MPA <twice /w, or MVPA <once /w. BMI: 1. 18.5–24.9 kg/m^2^; 0.5. 25.0–29.9 kg/m^2^; 0. ≥30 kg/m^2^ or <18.5 kg/m^2^. Diet (SSB, FFQ): 1. 0 g/d; 0.5. <250 g/d; 0. ≥250 g/d. Diet (fruit and vegetables, FFQ): 1. ≥5 servings/d; 0.5. 3–4.9 servings/d; 0. <3 servings/d. Diet (DF, FFQ): 1. ≥25 g/d; 0.5. 12.5–24.9 g/d; 0. <12.5 g/d. Diet (red/processed meat, FFQ): 1. <500 g/w and <3 g/w; 0.5. <500 g/w and 3–49.9 g/w; 0. ≥500 g/w and ≥50 g/w. Diet (sodium, FFQ): 1. ≤1500 mg/d; 0.5. 1501–2400 mg/d; 0. >2400 mg/d. | 8 | |
| Odegaard et al, 2013^93^ | Singapore Chinese Health Study | Singapore | 1993–2007 (11.49) | 45.60 | 45–74 (55.92) | Asian predominant | <31.62 | General population | 50466 | | Colorectal cancer cases were identified through the Singapore Cancer Registry and the Singapore Registry of Births and Deaths. | Smoking: 2. never smokers; 1. start to smoke after 15 years or <13 cigarettes/d; 0. start to smoke before 15 years and ≥13 cigarettes/d. Alcohol drinking: 2. 0–7 drinks/w; 1. 8–14 drinks/w; 0. >14 drinks/w. PA (strenuous PA or vigorous work): 2. ≥1.5 h/w; 0. <1.5 h/w. BMI: 1. 18.5–27.4 kg/m^2^; 0. <18.5 kg/m^2^ or ≥27.4 kg/m^2^. Diet (vegetable–fruit–soy dietary pattern score, FFQ): 2. highest 25th percentile; 1. middle 50th percentile; 0. lowest 25th percentile. Sleeping: 1. 6–8 h/d; 0. <6 h/d or ≥9 h/d. | 7 | |
| Petimar et al, 2019^27^ | Nurses’ Health Study and Health Professionals Follow-up Study | US | 1986–2012 (>24.00) | 39.72 | 40–75 (52.80) | White predominant | Predominant | General population | 114419 | | Colorectal cancer cases (ICD-9, 153–154) were self-reported (confirmed by medical records) or through next of kin, the National Death Index, death certificates, or medical records. | Diet (consumption of fruits and vegetables, FFQ): 0.143. ≥5 servings/d; 0.071. 2.5–4.9 servings/d; 0. <2.5 servings/d.  Diet (consumption of fiber, FFQ): 0.143. ≥30 g/d; 0.071. 15–29 g/d; 0. <15 g/d.  Diet (consumption of whole grains or pulses, FFQ): 0.143. ≥3 servings/d; 0.071. 1.5–2.9 servings/d; 0. <1.5 servings/d.  Diet (consumption of refined grains, pastries, sweets, and salty snacks, FFQ): 0.143. <1.5 servings/d; 0.071. 1.5–2.9 servings/d; 0. ≥3 servings/d.  Diet (consumption of red meat, FFQ): 0.071. ≤3 servings/w; 0.004. 3.1–5.9 servings/w; 0. ≥6 servings/w.  Diet (consumption of processed meat, FFQ): 0.071. <3 g/d; 0.004. 3–26 g/d; 0. ≥27 g/d.  Diet (intake of sodas and other beverages with added sugars, FFQ): 0.071. 0 drinks/d; 0.004. 0.1–0.9 drinks/d; 0. ≥1 drink/d.  Diet (intake of juices, FFQ): 0.071. <1 drink/d; 0.004. 1–1.9 drinks/d; 0. ≥2 drinks/d.  Alcohol drinking (M/F): 0.143. 0 drinks/d; 0.071. 0.1–1.9/0.9 drinks/d; 0. ≥2/1 drinks/d.  BMI: 0.333. 18.5–24.9 kg/m^2^; 0.167. 15–18.4 or 25–29.9 kg/m^2^; 0. <15 or ≥30 kg/m^2^.  Weight gain in last 10 years: 0.333. no; 0.167. 0–9.9 lb.; 0. ≥10 lb..  WC (M/F): 0.333. <37/31.5 inches; 0.167. 37–40.1/31.5–34.5 inches; 0. ≥40.2/34.6 inches.  MPA: 0.5. ≥150 min/w; 0.25. 75–149 min/w; 0. <75 min/w.  TV watching: 0.5. <5 h/w; 0.25. 5–19.9 h/w; 0. ≥20 h/w. | 8 | |
| Rasmussen-Torvik et al, 2013^46^ | Atherosclerosis Risk in Communities Study | US | 1987–2006 (NA) | 45.50 | 45–64 (54.11) | White 74.89  Black 25.11 | 80.00 | General population | 13253 | | Cancer cases (excluding nonmelanoma skin cancers) were identified through cancer registries and hospital surveillance. | Smoking: 1. never smokers or quitting >12 m; 0. quitting ≤12 m or current smokers. PA: 1. MVPA ≥150 min/w or VPA ≥75 min/w; 0. MVPA <150 min/w and VPA <75 min/w. BMI: 1. <25 kg/m^2^; 0. ≥25 kg/m^2^. Diet (AHA, FFQ): 1. 4–5 components; 0. 0–3 components. SBP/DBP: 1. <120 and 80 mmHg (untreated); 0. ≥120 or 80 mmHg, or <120/80 mmHg (treated). FSG: 1. <100 mg/dl (5.55 mmol/l, untreated); 0. ≥100 mg/dl (5.55 mmol/l) or <100 mg/dl (5.55 mmol/l, treated). TC: 1. <200 mg/dl (6.22 mmol/l, untreated); 0. ≥200 mg/dl (6.22 mmol/l) or <200 mg/dl (6.22 mmol/l, treated). | 9 | |
| Romaguera et al, 2012^22^ | European Prospective Investigation into Cancer and Nutrition | Europe | 1992–NA (11.00) | 32.68 | 25–70 (52.12) | White predominant | <68.30 | General population | 386355 | | Cancer cases were identified through population cancer registries, health insurance records, cancer pathology registries, and active follow-up. | Alcohol drinking (M/F): 1. ≤20/10 g/d; 0.5. 20–30/10–20 g/d; 0. >30/20 g/d. PA: 1. manual/heavy manual job, or VPA >2 h/w, or cycling/sports >30 min/d; 0.5. cycling/sports 15–30 min/d; 0. cycling/sports <15 min/d. BMI: 1. 18.5–24.9 kg/m^2^; 0.5. 25–29.9 kg/m^2^; 0. <18.5 kg/m^2^ or ≥30 kg/m^2^. Diet (energy density, FFQ): 0.5. ≤125 kcal/(100g.d); 0.25. 126–175 kcal/(100g.d); 0. ≥175 kcal/(100g.d). Diet (SSB, FFQ): 0.5. 0 g/d; 0.25. ≤250 g/d; 0. >250 g/d. Diet (fruit and vegetables, FFQ): 0.5. ≥400 g/d; 0.25. 200–399 g/d; 0. <200 g/d. Diet (DF, FFQ): 0.5. ≥25 g/d; 0.25. 12.5–24.9 g/d; 0. <12.5 g/d. Diet (red and processed meat, FFQ): 1. <500 g/w and <3 g/d; 0.5. <500 g/w and 3–49.9 g/w; 0. ≥500 g/w or ≥50 g/d.  Breastfeeding (for female only): 1. cumulative breastfeeding ≥6 months; 0.5 cumulative breastfeeding >0 to <6 months; 0. no breastfeeding. | 8 | |
| Sheikh et al, 2019^96^ | Golestan Cohort Study | Iran | 2004–2017 (10.00) | 42.51 | 40–75 (52.05) | White predominant | <31.10 | General population | 50045 | | Esophageal squamous cell carcinoma cases were collected by active annual telephone surveys, home visits (when telephone contact was not successful), and monthly review of provincial cancer and death registration data. | Opium smoking: 7. never; 5. mild; 4. moderate; 0. Heavy.  Diet (daily intake of fruits, FFQ): 4. ≥3 servings; 1. 1–2 servings; 0. <1 serving.  Diet (daily intake of vegetables, FFQ): 5. ≥3 servings/d; 0. ≤2 servings/d.  Drinking hot tea: 5. only warm tea or ≤3 cups/d; 1. 4–6 cups/d; 0. >6 cups/d.  Excessive tooth loss: 6. the first two quartiles; 2. third quartile; 0. highest quartile.  Drinking un–piped water: 8. ≥31 years ago; 2. 1–30 years ago; 0. continued.  Exposure to indoor air pollution: 5. never; 2. ≥20 years ago; 1. 1–19 years ago; 0. continued. | 8 | |
| Thomson et al, 2014^29^ | Women’s Health Initiative Observational Study | US | 1993–NA (12.60) | 0 | 50–79 (63.23) | White 88.57  Black 7.01  Asian 2.97 | >79.57 | Postmenopausal women | 65838 | | Cancer cases (other than nonmelanoma skin cancer) were identified through self-reported data and verified through medical records. | Alcohol drinking: 2. nondrinker; 1. 0.1–1 drink/d; 0. >1 drink/d PA: 2. >17.5 MET–h/w; 1. 8.75–17.5 MET–h/w; 0. <8.75 MET–h/w. BMI: 2. <25 kg/m^2^ at age 18 years and baseline; 1. 25–29.9 kg/m^2^ at age 18 years and baseline; 0. ≥30 kg/m^2^ at age 18 years and baseline. Diet (score consisting of fruit and vegetable intake, total carotenoids level, whole grains%, and red and processed meat, FFQ): 2. 7–9 components; 1. 3–6 components; 0. 0–2 components. | 8 | |
| Wang et al, 2017^99^ | Singapore Chinese Health Study | Singapore | 1993–2014 (16.90) | 44.49 | 45–74 (56.40) | Asian predominant | <28.39 | General population | 61321 | | Gastric cancer cases (ICD-O-3, C16.0–C16.9) were identified through the Singapore Cancer Registry. | Smoking: 1. ≤21.9 pack–years; 0. >21.9 pack–years. Alcohol drinking: 1. ≤8.1 g/d; 0. >8.1 g/d. BMI: 1. <27.5 kg/m^2^; 0. ≥27.5 kg/m^2^. Diet (sodium, FFQ): 1. ≥782 g/1000kcal energy; 0. <782 g/1000kcal energy. Diet (dietary pattern score, FFQ): 1. ≥62 points; 0. <62 points. | 7 | |
| Wang et al, 2018^100^ | Health Professionals Follow-up Study & Nurses’ Health Study | US | 1984–2012 (21.52) | 38.18 | 38–75 (63.01) | White predominant | Predominant | General population | 120007 | | Incident digestive system cancer was reported through biennial follow-up questionnaires through 2012 for both cohorts and was confirmed by medical records. | Empirical lifestyle index for hyperinsulinemia=0.433*BMI (kg/m^2^)+0.039*Margarine (servings/d)+0.035*Liquor (servings/d)+0.035*Cream soups (servings/d)++0.028*Butter (servings/d)+0.026*Fruit juice (servings/d)+0.026*Red meat (servings/d)–0.046*Whole fruit (servings/d)–0.047*Coffee (servings/d)–0.036*Wine (servings/d)–0.032*PA (MET h/w)–0.032*High fat diary (servings/d)–0.026*Snacks (servings/d)–0.020*Salad dressing (servings/d) | 8 | |
| Warren Andersen et al, 2016 (1)^50^ | the Southern Community Cohort Study | US | 2002–2011 (6.00) | 40.30 | 40–79 (50.00) | White 27.00  Black 69.00 | 71.90 | General population | 61098 | | Cancer cases (invasive cancers for all primary sites and in situ bladder cancers) were identified through cancer registries. | Alcohol drinking (M/F): 1. 0.1– ≤2/1 drink/d; 0. none or >2/1 drinks/d. PA: 1. MPA ≥150 min/w, or VPA ≥75 min/w, or MVPA ≥150 min/w; 0. MPA <150 min/w, and VPA <75 min/w, and MVPA <150 min/w. BMI: 1. 18.5–24.9 kg/m^2^; 0. <18.5 kg/m^2^ or ≥25.0 kg/m^2^. Diet ( score consisting of processed meat and red meat, whole grains, and vegetables and fruits, FFQ): 1. 2–3 points; 0. 0–1 point. | 7 | |
| Xu et al, 2019^51^ | Alberta's Tomorrow Project | Canada | 2001–2016 (11.70) | 37.10 | 35–69 (50.50) | White predominant | >72.50 | General population | 25100 | | Incident cancer was identified via linkage with the Alberta Cancer Registry. | Alcohol drinking (M/F): 1. ≤2/1 drink/d; 0. >2/1 drink/d.  PA: 1. moderate/vigorous–intensity recreational physical activity <210 min/w over the last 12 months; 0. moderate/vigorous–intensity recreational physical activity ≥210 min/w over the last 12 months.  BMI: 1. <25.0 kg/m^2^; 0. ≥25.0 kg/m^2^.  Diet (fruit and vegetables, FFQ): 1. ≥5 servings/d over the past 12 months; 0. <5 servings/d over the past 12 months.  Diet (red meat, FFQ): 1. ≥500 g/w; 0. <500 g/w.  Diet (dietary supplements, FFQ): 1. no dietary supplement over the past 12 months; 0. at least one dietary supplement over the past 12 months. | 8 |  |
| Zhang et al, 2017 (2)^105^ | Shanghai Men’s Health Study | China | 2004–2013 (9.28) | 100 | 40–74 (55.31) | Asian predominant | 59.90 | General population | 59503 | | Gastric cancer cases (ICD-9, 151) were identified through active surveillance, the Shanghai Cancer Registry, and the Shanghai Municipal Vital Statistics. | 5–point score: Smoking: 1. never smokers or quitting ≥10 years; 0. ever smokers or quitting <10 years. Alcohol drinking: 1. ≤14 drinks/w; 0. >14 drinks/w. PA (MVPA): 1. ≥150 min/w; 0. <150 min/w. BMI: 1. 18.5–23.9 kg/m^2^; 0. ≥24 kg/m^2^ or <18.5 kg/m^2^. Diet (Chinese Food Pagoda score consisting of grains, vegetables, fruits, dairy, beans, meat and poultry, fish and shrimp, eggs, fats and oils, and salt, FFQ): 1. >median (34.06 points); 0. ≤median. 11–point score: Smoking: 3. never smokers; 2. former smokers; 1. current smokers <20 cigarettes/d or <20 years; 0. current smokers ≥20 cigarettes/d and ≥20 years. Alcohol drinking: 2. 0.1–14 drinks/w; 1. none; 0. >14 drinks/w. PA: 2. ≥150 min/w; 1. 1–150 min/w; 0. none. BMI: 2. 18.5–23.9 kg/m^2^; 1. 24.0–27.9 kg/m^2^; 0. ≥28.0 kg/m^2^ or <18.5 kg/m^2^. Diet (Chinese Food Pagoda score, FFQ): 2. ≥35.9 points; 31.9–35.8 points; 0. <31.9 points. | 8 | |
| Zhang et al, 2018^106^ | Shanghai Men’s Health Study | China | 2002–2013 (9.28) | 100 | 40–74 (55.31) | Asian predominant | 59.90 | General population | 59503 | | Colorectal cancer cases (ICD-9, 153–154) were identified through the Shanghai Cancer Registry and the Shanghai Municipal Vital Statistics. | Smoking: 1. never smokers or quitting ≥10 years; 0. ever smokers or quitting <10 years. Alcohol drinking: 1. ≤14 drinks/w; 0. >14 drinks/w. PA (MVPA): 1. ≥150 min/w; 0. <150 min/w. WHR: 1. <0.9; 0. ≥0.9. Diet (Chinese Food Pagoda score consisting of grains, vegetables, fruits, dairy, beans, meat and poultry, fish and shrimp, eggs, fats and oils, and salt, FFQ): 1. top three quintiles; 0. lower two quintiles | 8 | |

*%E* percentage of total energy intake; *ACS* American Cancer Society; *AHA* American Heart Association; *AHEI* Alternative Healthy Eating Index; *AICR* American Institute for Cancer Research; *ASCVD* atherosclerotic cardiovascular disease; *BMI* body mass index; *CBVD* cerebrovascular disease; *CHD* coronary heart disease; *CRF* cardiorespiratory fitness; *CVD* cardiovascular disease; *DASH* Dietary Approaches to Stop Hypertension; *DBP* diastolic blood pressure; *DF* dietary fiber; *DHA* docosahexaenoic acid; *DM* diabetes mellitus; *ECG* electrocardiogram; *EPA* eicosapentaenoic acid; *FA* fatty acid; *FBG* fasting blood glucose; *FFQ* food frequency questionnaire; *FPG* fasting plasm glucose; *FSG* fasting serum glucose; *HbA1c* glycosylated hemoglobin; *HF* heart failure; *ICD* International Classification of Diseases; *IHD* ischemic heart disease; *LTPA* leisure time physical activity; *M/F* for male and female respectively; *MDS* Mediterranean diet score; *MET* metabolic equivalent of task; *MI* myocardial infarction; *mMDS* modified Mediterranean diet score; *MPA* moderate physical activity; *MVPA* moderate to vigorous physical activity; *NA* not available; *NOS* Newcastle-Ottawa Scale; *PA* physical activity; *PUFA* polyunsaturated fatty acid; *SBP* systolic blood pressure; *SFA* saturated fatty acid; *SSB* sugar-sweetened beverage; *TC* total cholesterol; *UK* the United Kingdom; *US* the United States; *VPA* vigorous physical activity; *WC* waist circumference; *WCRF* World Cancer Research Fund; *WHO* World Health Organization; *WHR* waist-to-hip ratio

^a^The percentage of ethnic groups may not sum to 100% since some participants belonged to the other ethnic groups or did not report the information

Supplementary Table 6 Characteristics of studies related to cancer mortality

| Author-year | Cohort | Country | Follow–up duration (mean or median) | Men (%) | Age (mean) | Ethnicity (%)^a^ | Proportion of high school graduates (%) | Health status | Sample size | Outcome attainment | Definition of healthy lifestyles | NOS score |
| --- | --- | --- | --- | --- | --- | --- | --- | --- | --- | --- | --- | --- |
| Artero et al, 2012^b, 53^ | Aerobics Center Longitudinal Study | US | 1987–2003 (11.60) | 75.67 | 20–88 (46.00) | White >99.00 | >70.00 | General population | 11993 | Cancer mortality (ICD-9, 140–208; ICD-10, C00–C97) was identified through the National Death Index and death certificates. | Smoking: 1. never smoker; 0. ever smoker; PA: 1. ≥500 MET–min/w; 0. <500 MET–min/w. BMI: 1. 18.5–24.9 kg/m^2^; 0. ≥25 kg/m^2^. Diet (AHA, 3–d dietary record): 1. 3–4 components; 0. 0–2 components. SBP/DBP: 1. <120 and 80 mmHg (not treated); 0. <120 and 80 mmHg (treated) or ≥120 or 80 mmHg. FPG: 1. <100 mg/dl (5.55 mmol/l, not treated); 0. <100 mg/dl (5.55 mmol/l, treated) ≥100 mg/d (5.55 mmol/l). TC: 1. <200 mg/dl (6.22 mmol/l, not treated); 0. ≥200 mg/dl (6.22 mmol/l, treated). | 9 |
| Bonaccio et al, 2019^56^ | Moli-sani Study | Italy | 2005–2015 (8.20) | 47.70 | 35–NA (55.00) | White predominant | 47.74 | General population | 22839 | All–cause mortality, CVD mortality (ICD-9, 390–459), and cancer mortality were assessed by the Italian mortality registry and validated by Italian death certificates. | Smoking: 1. abstention from smoking; 0. current smoking.  PA: 1. LTPA ≥30 min/d; 0. LTPA <30 min/d.  WHR (M/F): 1. <0.90/0.85; 0. ≥0.90/0.85.  Diet (MDS, FFQ): 1. above the sex–specific medians; 0. not above the sex–specific medians. | 9 |
| Breslow et al, 1980^57^ | "Alameda cohort" | US | 1965–1974 (8.61) | 44.22 | NA (<53.28) | White 84.00 | NA | General population | 4864 | Cancer mortality (ICD-8, 390–458) was identified through active follow–up. | Smoking: 1. never smoker; 0. ever smoker. Alcohol drinking: 1. ≤4 drinks/episode; 0. >4 drinks/episode. PA: 1. often or sometimes engage in active sports, swim or take long walks, or often garden or do physical exercises; 0. not often or sometimes engage in active sports, swim or take long walks, or often garden or do physical exercises. BMI (M/F): 1. between 20%–95%/10%–90% desirable weight for height; 0. <20%/10% or >95%/90% desirable weight for height. Diet (eating breakfast almost every day): 1. yes; 0. no. Diet (eating between meals once in a while, rarely or never): 1. yes; 0. no. Sleep: 1. 7–8 h/d; 0. <7 or >8 h/d. | 5 |
| Cerhan et al, 2004^b, 37^ | Iowa Women’s Health Study | US | 1986–1998 (11.39) | 0 | 55–69 (61.70) | White predominant | 86.10 | Postmenopausal women | 29838 | Cancer mortality was determined by linkage to Iowa death certificates. | Alcohol drinking: 1. <1 drink/d (14 g/d); 0. ≥1 drinks/d. PA: 1. exercise moderately daily and vigorously ≥1 h/w; 0. exercise moderately <once /d or vigorously <1 h/w. BMI: 1. ≤25.0 kg/m^2^; 0. > 25.0 kg/m^2^. Weight gain since age 18: 1. <11 pounds; 0. ≥11 pounds. Diet (vegetable and fruit intake excluding pulses and starchy, FFQ): 1. ≥5 servings/d; 0. <5 servings/d. Diet (complex carbohydrates intake, FFQ): 1. ≥400 g/d; 0. <400 g/d. Diet (red meat intake, FFQ): 1. <80 g/d; 0. ≥ 80g/d. Diet (consumption of fat as percentage total calories, FFQ): 1. ≤30%; 0. >30%. Diet (sodium, FFQ): 1. <2400 mg/d; 0. ≥2400 mg/d. | 7 |
| Cheng et al, 2018 (1)^59^ | Iowa Women’s Health Study | US | 1986–2012 (≥16.63) | 0 | 55–69 (61.70) | White predominant | 86.10 | Postmenopausal women | 35221 | Deaths were identified through the State Health Registry of Iowa and the National Death Index. The underlying cause of death was assigned and coded by state vital registries according to the ICD. Cancer mortality was defined using ICD-9 codes 140–239 and ICD-10 codes C00–D48. | Smoking: 5. never smoker; 1.67. former smoker; 0.35. current smoker.  PA: 5.81. VPA ≥twice/w or MPA >4 times/w; 3.49.VPA once/w plus MPA once/w, or MPA 2–4 times/w; 1. no VPA or MPA <twice/w.  BMI: 5. <25.0 kg/m^2^; 3.03. 25.0–29.9 kg/m^2^; 0.91. ≥30.0 kg/m^2^. | 8 |
| Eguchi et al, 2017^63^ | Japan Collaborative Cohort Study | Japan | 1988–2009 (19.30) | 43.24 | 40–79 (55.52) | Asian predominant | 63.88 | General population | 42647 | Cause and date of death were determined by reviewing death certificates. ICD-10 for cancer was not available. | Smoking: 1. not a current smoker; 0. current smoker. Alcohol drinking: 1. <2 gou/d (46g ethanol/d); 0. ≥2 gou/d. PA: 1. ≥0.5 h/d or ≥5 h/w; 0. <0.5 h/d and <5 h/w. BMI: 1. 21–25 kg/m^2^; 0. <21 kg/m^2^ or >25 kg/m^2^. Diet (fruit): 1. ≥1 servings/d; 0. <1 serving/d. Diet (fish): 1. ≥1 servings/d; 0. <1 serving/d. Diet (milk): 1. almost daily; 0. <once /d. Sleeping: 1. 5.5–7.4 h/d; 0. <5.5 h/d or >7.4 h/d. | 8 |
| Fazel-Tabar Malekshah et al, 2016^65^ | Golestan Cohort | Iran | 2004–2015 (8.08) | 42.75 | 40–75 (51.54) | White >75.59 | <31.10 | General population | 40708 | Cancer mortality (ICD-10, not reported) were identified through active follow–up and confirmed by verbal autopsy and extensive medical documents. | Smoking: 1. never smoker; 0. ever smoker. PA: 1. MVPA ≥30 min/d; 0. MVPA <30 min/d. Diet (AHEI, FFQ): 1. highest 40%; 0. lower 60%. | 8 |
| Ford et al, 2011^66^ | National Health and Nutrition Examination Surveys III | US | 1988–2006 (>17.00) | 46.75 | 17–NA (59.00) | White 81.10  Black 11.10 | 61.88 | General population | 16958 | Malignant neoplasms mortality (ICD-10, C00–C97) was identified through the National Death Index. | Smoking: 1. <100 cigarettes; 0. ≥100 cigarettes. Alcohol drinking (M/F): 1. 0.1–59.9/29.9 drinks/m; 0. none or ≥60/30 drinks/m. PA: 1. VPA ≥3 times/w or MPA ≥5 times/w; 0. VPA <3 times/w and MPA <5 times/w. Diet (a single 24–h recall, HEI): 1. top 40%; 0. lower 60%. | 8 |
| Ford et al, 2012^67^ | National Health and Nutrition Examination Surveys 1999 | US | 1999–2006 (5.70) | 50.11 | 20–NA (45.60) | White 72.64  Black 19.15 | 52.90 | General population | 8375 | Malignant neoplasms (ICD-10, C00–C97) mortality was identified through the National Death Index. | Smoking: 1. not a current smoker; 0. current smoker. PA: 1. MVPA ≥150 min/w; 0. MVPA <150 min/w. Diet (HEI, a single 24–h recall): 1. top 40%; 0. lower 60%. | 8 |
| Gopinath et al, 2010^68^ | Blue Mountains Eye Study | Australia | 1992–2007 (>11.40) | NA | 49–NA (>62.51) | NA | NA | General population | 2283 | Cancer mortality was assessed using ICD-9 and identified through the Australian National Death Index data. | Smoking: 1. not a current smoker; 0. current smoker. Alcohol drinking (M/F): 1. ≤21/14 units/w; 0. >21/14 units/w. PA: 1. ≥3 times/w; 0. <3 times/w. Diet (fruits and vegetables consumption): 1. ≥3 times/d; 0. <3 times/d. | 8 |
| Greenlee et al, 2017^2^ | Cardiovascular Health Study | US | 1989–2011 (15.00) | 38.56 | 65–98 (72.00) | White 86.71  Black 11.72 | 72.91 | General population | 3491 | Cancer deaths were identified from National Death Index and interviews with proxy respondents. | ACS: Smoking: 2. never smokers or quitting >1 year; 1. quitting ≤1 year; 0. current smokers. Alcohol drinking (M/F): 2. non–drinker; 1. <2/1 unit/d; 0. >2/1 unit/d. PA: 2. LTPA ≥8.75 MET–h/w; 1. LTPA 0.10–8.74 MET–h/w; 0. 0 MET–h/w. BMI: 2. <25 kg/m^2^ at baseline and age 50; 1. 25–29.9 kg/m^2^ at baseline and <30 kg/m^2^ at age 50, or 25–29.9 kg/m^2^ at age 50 and <30 kg/m^2^ at baseline; 0. ≥30 kg/m^2^ at either baseline, age 50, or both. Diet (ACS, including vegetables and fruits, red and processed meats, and whole grains consumption, FFQ): 2. ≥6 score; 1. 3–5 score; 0. <3 score. AHA: Smoking: 2. never smokers or quitting >1 year; 1. quitting ≤1 year; 0. current smokers. PA: 2. LTPA ≥8.75 MET–h/w; 1. LTPA 0.10–8.74 MET–h/w; 0. 0 MET–h/w. BMI: 2. <25 kg/m^2^; 1. 25–29.9 kg/m^2^; 0. ≥30 kg/m^2^. Diet (AHA, FFQ): 2. 4–5 components; 1. 2–3 components; 0. 0–1 components. SBP/DBP: 2. <120 and 80 mmHg (untreated); 1. 120–139 or 80–89 mmHg (untreated) or <120 and 80 mmHg (treated); 0. ≥140 or 90 mmHg. FPG: 2. <100 mg/dl (5.55 mmol/l, untreated); 1. 100–125 mg/dl (5.55–6.99 mmol/l­, untreated) or <100 mg/dl (5.55 mmol/l, treated); 0. ≥126 mg/dl (7.00 mmol/l). TC: 2. <200 mg/dl (5.18 mmol/l, untreated); 1. 200–239 mg/dl (5.18–6.21 mmol/l, untreated) or <200 mg/dl (5.18 mmol/l, treated); 0. ≥ 240 mg/dl (6.22 mmol/l). | 8 |
| Hamer et al, 2011^70^ | National Diet and Nutrition Survey | UK | NA–2008 (9.20) | 50.75 | 65–99 (76.50) | White predominant | NA | General population | 1062 | Cancer mortality was identified through National Health Service administrative mortality data. | Smoking: 1. never smoker; 0. ever smoker. Alcohol drinking (M/F): 1. 1–21/14 units/w; 0. 0 or >21/14 units/w. PA: 1. regular moderate to vigorous PA; 0. irregular or no moderate to vigorous PA. Diet (daily vitamin C intake): 1. ≥50 mU; 0. <50 mU. | 8 |
| Hastert et al, 2014^73^ | Vitamins and Lifestyle Study | US | 2000–2010 (7.70) | 50.20 | 50–76 (60.70) | White 97.20  Black 1.10  Asian <2.30 | >81.70 | General population | 54370 | Cancer–cause mortality (malignant neoplasm, ICD-10, C00–C97) was tracked through the Washington State death file. | Alcohol drinking (M/F): 1. ≤2/1 drinks/d; 0. > 2/1 drinks/d. PA: 1. moderate/fast walking or moderate/strenuous activities ≥30 min/d on ≥5 d/w in ≥7 years of the past 10 years; 0. moderate/fast walking or moderate/strenuous activities <30 min/d, or ≥5 d/w, or in ≥7 years of the past 10 years. BMI: 1. 18.5–24.9 kg/m^2^ at age 18, 30, 45 and at baseline; 0. <18.5 kg/m^2^ or ≥25.0 kg/m^2^ at age 18, 30, 45 or at baseline. Diet (energy–density and SSB, FFQ): 1. <125 kcal/100g and <1 drink/w; 0. ≥125 kcal/100g or ≥1 drinks/d. Diet (plant foods, FFQ): 1. fruits and vegetables ≥5 servings/d and whole grains/legumes ≥1 serving/d; 0. fruits and vegetables <5 servings/d or whole grains/legumes <1 serving/d. Diet (red meat and processed meat, FFQ): 1. <18 oz/w; 0. ≥18 oz/w. | 8 |
| Heitz et al, 2017^74^ | 4-Corners Women Health Study | US | 1999–2009 (<10.00) | 0 | 25–79 (55.21) | White 100 | 88.17 | Women with invasive breast cancer | 837 | The stage at diagnosis of breast cancer was obtained from the New Mexico Tumor Registry, and breast cancer mortality (ICD-10, C50) was identified through the National Death Index. | Smoking: 2. never smokers; 1. former smokers; 0. current smokers. Alcohol drinking: 2. ≤0.5 drinks/d; 1. 0.5–1 drinks/d; 0. >1 drinks/d. PA: 2. VPA >75 min/w; 1. VPA ≤75 min/w; 0. none VPA. BMI: 2. <25 kg/m^2^; 1. 25–30 kg/m^2^; 0. ≥30 kg/m^2^. WHR: 2. <0.775; 1–0.775–0.84; 0. ≥0.84. Diet (score consisting of dairy fat, refined grains, snacks, gravies and sauces, potatoes, bacon, beef, sugary drinks and desserts, prepared foods, fast foods, and fresh fruits and vegetables): 2. lowest tertile; 1. medium tertile; 0. highest tertile. | 7 |
| Inoue-Choi et al, 2013^75^ | Iowa Women’s Health Study | US | 2004–2009 (5.40) | 0 | 73–87 (78.90) | White predominant | 86.10 | Cancer patients | 2017 | Cancer mortality was identified through the State Health Registry of Iowa, supplemented with the National Death Index (National Center for Health Statistics). | Alcohol drinking: 1. ≤10.0 g/d; 0.5. 10.1–20.0 g/d; 0. >20.0 g/d. PA: 1. ≥30.0 min/d; 0.5. 0.1–29.9 min/d; 0. none. BMI: 1. 18.5–24.9 kg/m^2^; 0.5 25.0–29.9 kg/m^2^; 0. ≥30.0 kg/m^2^. Diet (SSB, FFQ): 1. 0 g/d; 0.5. <250 g/d; 0. ≥250 g/d. Diet (fruit and vegetable, FFQ): 1. ≥5 servings/d; 0.5. 3–4 servings/d; 0. <3 servings/d. Diet (DF, FFQ): 1. ≥25.0 g/d; 0.5. 12.5–24.9 g/d; 0. <12.5 g/d. Diet (red meat and processed meat, FFQ): 1. <500 g/d and <3 g/d; 0.5. <500 g/d and 3–49 g/d; 0. ≥500 g/d or ≥50 g/d. Diet (sodium, FFQ): 1. ≤1500 mg/d; 0.5. 1501–2400 mg/d; 0. >2400 mg/d. | 8 |
| Kabat et al, 2015^31^ | American Association of Retired Persons | US | 1995–2008 (12.60) | 60.21 | 50–71 (62.00) | White 91.46 | 92.65 | General population | 476396 | Cancer mortality was identified through Social Security Administration Death Master File, the National Death Index Plus, and cancer registry records, confirmed by questionnaires and other mailings. | 11–point score: Alcohol drinking (M/F): 2. 1–2/1 drinks/d; 1. nondrinkers; 0. ≥3/2 drinks/d. PA: 3. ≥5 times/w; 2. 3–4 times/w; 1. 1–2 times/w; 0. less than once/w. BMI: 3. 18.5–24.9 kg/m^2^; 2. 25.0–29.9 kg/m^2^; 1. 30.0–34.9 kg/m^2^; 0. ≥35 kg/m^2^. Diet (score consisting of fruit and vegetable, the ratio of whole grains to total grains, and red plus processed meats, FFQ): 3. 6–9 points; 2. 5 points; 1. 4 points; 0. 0–3 points. 4–point score: Alcohol drinking (M/F): 1. 1–2/1 drinks/d; 0. none or ≥3/2 drinks/d. PA: 1. ≥5 times/w; 0. <5 times/w. BMI: 1. 18.5–24.9 kg/m^2^; 0. ≥25.0 kg/m^2^. Diet (score consisting of fruit and vegetable, the ratio of whole grains to total grains, and red plus processed meats, FFQ): 1. 6–9 points; 0. 0–5 points. | 8 |
| Khaw et al, 2008^b,78^ | European Prospective Investigation into Cancer and Nutrition-Norfolk | UK | 1993–2006 (11.00) | 45.35 | 45–79 (58.13) | White 99.50 | 53.38 | General population | 20244 | Cancer mortality (ICD-9, 140–208; ICD-10, C00–C97) was identified through death certification at the Office of National Statistics. | Smoking: 1. not a current smoker; 0. current smoker. Alcohol drinking: 1. 1–14 units/w (1 unit≈8 g alcohol); 0. none or >14 units/w. PA: 1. LTPA ≥0.5 h/d; 0. LTPA <0.5 h/d. Diet (plant food intake, blood vitamin C level): 1. ≥50 mmol/l; 0. <50 mmol/l. | 9 |
| Knoops et al, 2004^81^ | Healthy Ageing: a Longitudinal study in Europe | Europe | 1988–2000 (10.00) | 64.43 | 70–90 (74.24) | White predominant | <66.86 | General population | 2339 | Identification of cancer mortality (ICD-9, 140–240) was not reported. | Smoking: 1. never smokers or quitting >15 years; 0. quitting ≤15 years or current smokers. Alcohol drinking: 1. >0 g/d; 0. none. PA (Voorrips or Morris questionnaire): 1. the intermediate and the highest tertile; 0. the lowest tertile. Diet (mMDS, dietary history method): 1. ≥4 points; 0. <4 points. | 8 |
| Kvaavik et al, 2010^82^ | Health and Lifestyle Survey | UK | 1985–2005 (20.00) | 51.35 | 18–NA (43.70) | White 98.00 | NA | General population | 4886 | Cancer mortality (ICD-9, 140–209) was ascertained from death certificates. | Smoking: 1. not a current smoker; 0. a current smoker. Alcohol drinking (M/F): 1. ≤21/14 units/w; 0. >21/14 units/w. PA: 1. ≥120 min/w; 0. <120 min/w. Diet (fruits and vegetables consumption, FFQ): 1. ≥3 times/d; 0. <3 times/d. | 8 |
| Lee et al, 2011^83^ | Aerobics Center Longitudinal Study | US | 1972–2003 (14.50) | 100 | 20–84 (43.11) | White 95.00 | >70.00 | General population | 24731 | Cancer mortality (ICD-9, 140–208; ICD-10, C00–C97) were identified from the National Death Index and official death certificates. | Smoking: 1. never smoking; 0. ever smoking. Fitness (CRF): 1. higher 80%; 0. lower 20%. WC: 1. <94 cm; 0. ≥94 cm. | 9 |
| Li et al, 2018^35^ | Nurses' Health Study & Health Professionals Follow-Up Study | US | 1980–2014 (27.20–33.90) | 36.00 | 34–75 (48.96) | White 96.34 | Predominant | General population | 123219 | Cancer mortality was identified from state vital statistics records, the National Death Index, reports by the families, and the postal system. | Smoking: 1. never smoker; 0. ever smoker.  Alcohol drinking (M/F): 1. 5–30/15 g/d; 0. <5 g/d or >30/15 g/d.  MVPA: 1. >30 min/d; 0. ≤30 min/d.  BMI: 1. 18.5–24.9 kg/m^2^; 0. <18.5 kg/m^2^ or ≥25 kg/m^2^.  Diet (AHEI, FFQ): 1. top 40% of each cohort distribution; 0. lower 60% of each cohort distribution. | 8 |
| Lin et al, 2012^84^ | Taichung Diabetes Study | Taiwan, China | 2002–2008 (4.02) | 51.93 | 30–NA (58.51) | Asian predominant | NA | Patients with type 2 diabetes mellitus | 5686 | Cancer mortality (ICD-9-CM, 140–208) was identified through the Taiwan National Death Index. | Smoking: 1. never smoker; 0. ever smoker. Alcohol drinking: 1. abstainer; 0. drinker. PA: 1. ≥once /w for >1 m continuously; 0. <once /w or <1 m continuously. Diet (carbohydrate intake, 24–h food diary): 1. <65%E; 0. ≥65%E. | 7 |
| Lohse et al, 2016^b, 85^ | MONItoring trends and determinants of CArdiovascular disease-Switzerland & National Research Programme1A | Switzerland | 1977–NA (21.70) | 50.60 | 25–74 (46.06) | White predominant | 58.85 | General population | 16722 | Cancer mortality (ICD-8, 140–209, 225, and 230–239; ICD-10, C00–C97, D32–D33, and D37–D48) including lung, colorectal, upper aerodigestive tract, lymphatic and hematopoietic tissue, pancreatic, urinary tract, liver, stomach, breast, female genital tract, and prostate cancer mortality, was identified through the Swiss National Cohort. | Alcohol drinking: 1. didn't drink yesterday; 0. drank yesterday. PA (MPA ≥60 min/d or VPA ≥30 min/d): 1. ≥2 d/w; 0.5. 1 d/w; 0. <1d/w. Sedentary behavior: 1. regular exercise or exhausting; 0.5. walking, cycling, other regular activities such as gardening, or average; 0. mostly sitting or sedentary. BMI: 1. 18.5–24.9 kg/m^2^; 0.5. 25–29.9 kg/m^2^; 0. <18.5 kg/m^2^ or ≥30 kg/m^2^. Diet (energy density, score consisting of fat for cooking, bread, or salad, cut away fat from meat, and sweets/chocolate): 1. 2–3 points; 0.5. 1 point; 0. 0 point. Diet (fruits and vegetables): 1. yesterday consume both fruits and vegetables; 0.5. yesterday consume either fruits or vegetables; 0. yesterday consume no fruits and vegetables. Diet (grains): 1. consume yesterday; 0. no yesterday. Diet (processed meat): 1. didn't consume yesterday; 0.5. consume meat yesterday; 0. consume sausage products yesterday. Diet (salt): 1. never adding salt; 0.5. sometimes adding salt; 0. always adding salt. | 9 |
| Martin-Diener et al, 2014^86^ | MONItoring trends and determinants of CArdiovascular disease-Switzerland & National Research Programme1A | Switzerland | 1977–2008 (21.35) | 48.60 | 16–90 (45.10) | White predominant | <65.77 | General population | 16721 | Cancer mortality (ICD-8, 140–239; ICD-10, C00–C99 and D00–D48) was identified through the Swiss National Cohort. | Smoking: 1. not a current smoker; 0. current smoker. Alcohol drinking (M/F): 1. <40/20 g/d; 0. >40/20 g/d. PA: 1. frequent walking or cycling, other frequent activities such as gardening, or regular VPA; 0. light PA, mostly sedentary. Diet (fruit intake on the previous day): 1. yes; 0. no. | 8 |
| McCullough et al, 2011^87^ | Cancer Prevention Study-II Nutrition Cohort | US | 1992–2006 (12.82) | 45.31 | 50–74 (62.67) | White 97.99 | 92.66 | Current non–smoker | 111966 | Cancer mortality (ICD-9, 140–195 and 199–208; ICD-10, C00–C76 and C80–C97) was identified through National Death Index. | Alcohol drinking (M/F): 2. 0.1–2/1 drinks/d; 1. none; 0. >2/1 drinks/d. PA: 2. ≥17.5 MET–h/w; 1. 8.75–17.4 MET–h/w; 0. <8.75 MET–h/w. BMI: 2. 18.5–24.9 kg/m^2^ at both time points; 1. 25–30 kg/m^2^ at both time points, or 18.5–24.9 kg/m^2^ at one time point and ≥25 kg/m^2^ at another time point; 0. >30 kg/m^2^ at both time points, or >30 kg/m^2^ at one time point and 25–30 kg/m^2^ at another time point. Diet (ACS, FFQ): 2. 7–9 points; 1. 3–6 points; 0. 0–2 points. | 7 |
| Meng et al, 1999^44^ | "Hawaii Department of Health survey" | US | 1975–1994 (15.36) | 49.50 | 18–NA (45.53) | White 31.06  Asian 62.61 | NA | General population | 31700 | Cancer mortality was identified through the mortality files from the Department of Health. | Smoking: 4. never smokers; 3. former smokers; 2. current smokers ≤1 ppd; 1. current smokers 1.1–1.5 ppd; 0. current smokers >1.5 ppd. Alcohol drinking (M/F): 1. 1–7/3 drinks/w; 0. none or >7/3 drinks/w. BMI: 3. 19.6–24.8 kg/m^2^; 2. <19.6 kg/m^2^ or 24.9–29.2 kg/m^2^; 1. 29.3–32.5 kg/m^2^; 0. ≥32.6 kg/m^2^. Diet (fat intake from animal products): 1. >385 g/w; 0. ≤385 g/w. Diet (fruit and vegetables consumption): 1. >1350 g/w; 0. ≤1350 g/w. | 8 |
| Nechuta et al, 2010^89^ | Shanghai Women’s Health Study | China | 1996–2007 (9.10) | 0 | 40–70 (<56.95) | Asian 100 | 42.10 | Non–smoker and non–drinker | 63791 | Cancer mortality (ICD-9, 140–208) was identified through Shanghai cancer, vital statistics registries, and death certificates. | 9–point score: Exposed to spouse smoke: 1. never; 0. ever. PA: 2. ≥2.0 MET h/d; 1. 0.1–1.99 MET h/d; 0. none. BMI: 2. 18.5–24.99 kg/m^2^; 1. 25.0–29.99 kg/m^2^; 0. ≥30.0 kg/m^2^ or <18.5 kg/m^2^. WHR: 2. tertile 1 ( <0.786); 1. tertile 2; 0. tertile 3 ( ≥0.830). Diet (fruit and vegetable intake, FFQ): 2. tertile 3 ( ≥626.5 g/d); 1. tertile 2; 0. tertile 1 ( <404.3 g/d). 5–point score: Exposed to spouse smoke: 1. never; 0. ever. PA: 1. ≥2.0 MET h/d; 0. <2.0 MET h/d. BMI: 1. 18.5–24.99 kg/m^2^; 0. ≥25.0 kg/m^2^ or <18.5 kg/m^2^. WHR: 1. tertile 1 ( <0.786); 0. tertile 2 and 3 ( ≥0.786). Diet (fruit and vegetable intake, FFQ): 1. tertile 3 ( ≥626.5 g/d); 0. tertile 2 and 1 ( <626.5 g/d). | 8 |
| Odegaard et al, 2011^c,1^ | Singapore Chinese Health Study | Singapore | 1993–2016 (20.60) | 45.10 | 45–74 (55.30) | Asian predominant | <32.70 | General population | 44052 | Cancer mortality (ICD-9, 140–208; ICD-10, C00–C97) was obtained through linkage with the nation-wide Singapore Birth and Death Registry. | 6–point score:  Smoking: 1. never smokers; 0. ever smokers.  Alcohol drinking (M/F): 1. 0.1–14/7 drinks/w; 0. none or >14/7 drinks/w.  PA: 1. moderate activity ≥2 h/w, or strenuous activity ≥0.5 h/w; 0. moderate activity <2 h/w, and strenuous activity <0.5 h/w.  BMI (aged <65/aged ≥65): 1. 18.5–21.4/24.4 kg/m^2^; 0. <18.5 or ≥21.5/24.5 kg/m^2^.  Diet (AHEI–2010, FFQ): 1. highest 40%; 0. Lower 60%.  Sleeping: 1. 7–8 h/d; 0. <7 or >8 h/d.  5–point score: smoking, drinking, PA, BMI, and diet.  4–point score: smoking, PA, BMI, and diet. | 8 |
| Petersen et al, 2015^94^ | Diet, Cancer and Health cohort study | Denmark | 1993–2010 (14.00) | 47.10 | 50–64 (55.53) | White predominant | 24.33 | General population | 51521 | Cancer mortality (ICD-10, C00–C97, and D00–D09) was identified through the Central Population Registry and Register of Causes of Death. | 5–point score: Smoking: 1. never smokers or quitting ≥15 years; 0. current smokers or quitting <15 years. Alcohol drinking (M/F): 1. ≤14/7 units/w; 0. >14/7 units/w. PA: 1. ≥30 min/d; 0. <30 min/d. WC: 1. ≤102 cm; 0. >102 cm. Diet (score consisting of fat, red and processed meat, fish, whole grain, and fruit and vegetable consumption, FFQ): 1. 2–5 components; 0. 0–1 component. 4–point score: smoking, drinking, PA, and diet. | 8 |
| Romaguera et al, 2015^95^ | European Prospective Investigation into Cancer and Nutrition | Europe | 1992–2009 (4.20) | 45.47 | NA (64.60) | White predominant | <57.90 | Patients with colorectal cancer | 3292 | Colorectal cancer mortality (ICD-10, not reported) was identified through health insurance records, cancer and pathology registries, and active follow-up through study subjects and their next-of-kin | Alcohol drinking (M/F): 1. ≤20/10 g/d; 0.5. 20.1–30/10.1–20 g/d; 0. >30/20 g/d. PA: 1. Manual/heavy manual job, or >2 h/w of VPA, or >30 min/d of cycling/sports; 0.5. cycling/sports 15–30 min/d; 0. cycling/sports <15 min/d. BMI: 1. 18.5–24.9 kg/m^2^; 0.5. 25–29.9 kg/m^2^; 0. <18.5 kg/m^2^ or ≥30 kg/m^2^. Diet (energy–dense, dietary questionnaires): 0.5. ≤125 kcal/100g/d; 0.25. 126–175 kcal/100g/d; 0. >175 kcal/100g/d. Diet (SSB, dietary questionnaires): 0.5. 0 g/d; 0.25. ≤250 g/d; 0. >250 g/d. Diet (fruit and vegetables, dietary questionnaires): 0.5. ≥400 g/d; 0.25. 200–399 g/d; 0. <200 g/d. Diet (DF, dietary questionnaires): 0.5. ≥25 g/d; 0.25. 12.5–24.9 g/d; 0. <12.5 g/d. Diet (red and processed meat, dietary questionnaires): 1. <500 g/w and <3 g/d; 0.5. <500 g/w and 3–49 g/d; 0. ≥500 g/w or ≥50 g/d. Cumulative breastfeeding: 1. ≥6 m; 0.5. 0.1–5.9 m; 0. 0 m. | 6 |
| Thomson et al, 2014^29^ | Women’s Health Initiative Observational Study | US | 1993–NA (12.60) | 0 | 50–79 (63.23) | White 88.57  Black 7.01  Asian 2.97 | >79.57 | Postmenopausal women | 65838 | Cancer mortality was identified through self-reported data and verified through Medical records and the National Death Index. | Alcohol drinking: 2. nondrinker; 1. 0.1–1 drink/d; 0. >1 drink/d PA: 2. >17.5 MET–h/w; 1. 8.75–17.5 MET–h/w; 0. <8.75 MET–h/w. BMI: 2. <25 kg/m^2^ at age 18 years and baseline; 1. 25–29.9 kg/m^2^ at age 18 years and baseline; 0. ≥30 kg/m^2^ at age 18 years and baseline. Diet (score consisting of fruit and vegetable intake, total carotenoids level, whole grains%, and red and processed meat, FFQ): 2. 7–9 components; 1. 3–6 components; 0. 0–2 components. | 8 |
| Van Dam et al, 2008^b, 34^ | Nurses’ Health Study | US | 1980–2004 (22.62) | 0 | 34–59 (NA) | White predominant | Predominant | General population | 77782 | Cancer mortality (ICD-8, 140–207) was identified through reports by next of kin, the postal authorities, death certificates, medical records, and National Death Index. | Smoking: 1. never smoker; 0. ever smoker. Alcohol drinking: 1. 1–14.9 g/d; 0. <1 g/d or ≥15.0 g/d. PA (MVPA): 1. ≥30 min/d; 0. <30 min/d. BMI: 1. 18.5–25.0 kg/m^2^; 0. >25.0 kg/m^2^. Diet (AHEI, FFQ): 1. upper two fifths; 0. lower three fifths. | 6 |
| van Lee et al, 2016^97^ | Rotterdam Study | Netherlands | 1990–2011 (20.00) | 40.47 | 55–NA (65.43) | White predominant | 69.73 | General population | 2987 | Cancer mortality (ICD-10, C00–C97, D00–D09, and D37–D48) was identified through municipal population registries. | Alcohol drinking (M/F): 10. ≤2/1 drink/d; 0. >6/4 drinks/d. PA: 10. ≥5 activities/w; 0. 0 activities/w. Diet (vegetable consumption, FFQ): 10. ≥200 g/d; 0. 0 g/d. Diet (fruit consumption, FFQ): 10. ≥200 g/d; 0. 0 g/d. Diet (DF, FFQ): 10. ≥14 g/4.2 MJ; 0. 0 g/4.2 MJ. Diet (fish consumption, FFQ): 10. ≥450 mg/d EPA+DHA; 0. 0 mg/d EPA+DHA. Diet (SFA, FFQ): 10. ≤10%E; 0. >15%E. Diet (mono trans–FA, FFQ): 10. ≤1%E; 0. >1%E. Diet (sodium, FFQ): 10. ≤1.68 g/d; 0. >2.52 g/d. | 9 |
| Vergnaud et al, 2013^98^ | European Prospective Investigation into Cancer and Nutrition | Europe | 1992–2010 (12.80) | 32.05 | 25–70 (51.90) | White predominant | <67.34 | General population | 378864 | Cancer mortality (ICD-10, C00–D48) was identified through cancer registries, boards of health, and death indexes, or active follow-up including inquiries to participants, municipal registries, regional health departments, physicians and hospitals. | Alcohol drinking (M/F): 1. ≤20/10 g/d; 0.5. 21–30/11–20 g/d; 0. >30/20 g/d. PA: 1. manual/heavy manual job, or >2 h/w of vigorous PA, or >30 min/d of cycling/sports; 0.5. cycling/sports 15–30 min/d; 0. cycling/sports <15 min/d. BMI: 1. 18.5–24.9 kg/m^2^; 0.5. 25–29.9 kg/m^2^; 0. <18.5 kg/m^2^ or ≥30.0 kg/m^2^. Diet (energy–density, dietary questionnaires): 0.5. ≤125 kcal/100g/d; 0.25. 126–175 kcal/100g/d; 0. >175 kcal/100g/d. Diet (SSB, dietary questionnaires): 0.5. 0 g/d; 0.25. ≤250 g/d; 0. >250 g/d. Diet (fruit and vegetable consumption, dietary questionnaires): 0.5. ≥400 g/d; 0.25.200–399 g/d 0. <200 g/d. Diet (DF, dietary questionnaires): 0.5. ≥25 g/d; 0.25. 12.5–24.9 g/d; 0. <12.5 g/d. Diet (red and processed meat consumption, dietary questionnaires): 1. <500 g/w and <3 g/d; 0.5. <500 g/w and 3–49 g/d; 0. ≥500 g/w or ≥50 g/d. Cumulative breastfeeding: 1. ≥6 m; 0.5. 0.1–5.9 m; 0. 0 m. | 7 |
| Warren Andersen et al, 2016 (2)^101^ | the Southern Community Cohort Study | US | 2002–2011 (<10.00) | 40.66 | 40–79 (50.65) | White 31.36  Black 68.64 | 73.06 | General population | 75689 | Cancer mortality (ICD-10, C00–C97) was identified through the Social Security Administration’s Death Master File and National Death Index. | Smoking: 1. never smoker; 0. ever smoker. Alcohol drinking (M/F): 1. ≤2/1 drink/d; 0. >2/1 drink/d. PA: 1. MPA ≥150 min/w, or VPA ≥75 min/w, or MVPA ≥150 min/w; 0. MPA <150 min/w, and VPA <75 min/w, and MVPA <150 min/w. Sedentary behavior: 1. ≤5.75 h/d; 0. >5.75 h/d (lowest quartile). Diet (HEI, FFQ): 1. >66.7 points (highest quartile); 0. ≤66.7 points. | 7 |
| Wingard et al, 1982^102^ | Human Population Laborator | US | 1965–1974 (<10.00) | 47.17 | 30–69 (<53.28) | NA | NA | General population | 4725 | Cancer mortality was identified through California Death Registry. | Smoking: 1. never smoker; 0. ever smoker. Alcohol drinking: 1. <45 drinks/m; 0. >45 drinks/m. PA: 1. active; 0. inactive. Quetelet index (weight in pounds/(height in inches)^2^) based on Metropolitan Life Insurance reports: 1. 9.9% underweight–29.9% overweight; 0. extreme underweight or overweight. Sleeping: 1. 7–8 h/night; 0. <7 h/night or >8 h/night. | 7 |
| Yun et al, 2012^103^ | Korean Cancer Prevention Study | South Korea | 1994–2009 (10.30) | 53.14 | 30–84 (47.17) | Asian predominant | NA | General population | 59941 | Cancer mortality (ICD-10, C00–C99) was identified through the National Statistical Office. | 5–point score: Smoking: 1. never smokers or quitting ≥10 years; 0. current smokers or quitting <10 years. Alcohol drinking (M/F): 1. ≤2/1 drinks/d; 0. >2/1 drinks/d. PA: 1. ≥3 times/w; 0. <3 times/w. BMI: 1. ≤25 kg/m^2^; 0. >25 kg/m^2^. Diet (fruit and vegetable consumption): 1. highest quartile; 0. lower 3 quartiles. 4–point score: smoking, drinking, PA, and BMI. | 7 |
| Zhang et al, 2017 (1)^104^ | Shanghai Men’s Health Study | China | 2002–2013 (9.29) | 100 | 40–74 (55.34) | Asian predominant | 59.86 | General population | 59747 | Cancer mortality (ICD-9, 140–208) was identified through Shanghai Vital Statistics. | Smoking: 1. never smokers or quitting ≥10 years; 0. current smokers or quitting <10 years. Alcohol drinking: 1. ≤14 drinks/w; 0. >14 drinks/w. PA: 1. MVPA ≥150 min/w (2 MET–h/d); 0. MVPA <150 min/w. Diet (Chinese Food Pagoda score consisting of grains, vegetables, fruits, dairy, beans, meat and poultry, fish and shrimp, eggs, fats and oils, and salt, FFQ): 1. top three quintiles; 0. lower two quintiles. | 8 |

*%E* percentage of total energy intake; *ACS* American Cancer Society; *AHA* American Heart Association; *AHEI* Alternative Healthy Eating Index; *BMI* body mass index; *CRF* cardiorespiratory fitness; *DBP* diastolic blood pressure; *DF* dietary fiber; *FA* fatty acid; *FFQ* food frequency questionnaire; *FPG* fasting plasma glucose; *HEI* Healthy Eating Index; *ICD* International Classification of Diseases; *LTPA* leisure time physical activity; *M/F* for male and female respectively; *MET* metabolic equivalent of task; *mMDS* modified Mediterranean diet score; *MPA* moderate physical activity; *MVPA* moderate to vigorous physical activity; *NA* not available; *NOS* Newcastle-Ottawa Scale; *PA* physical activity; *SBP* systolic blood pressure; *SFA* saturated fatty acid; *SSB* sugar-sweetened beverage; *TC* total cholesterol; *UK* the United Kingdom; *US* the United States; *VPA* vigorous physical activity; *WC* waist circumference; *WHR* waist-to-hip ratio

^a^The percentage of ethnic groups may not sum to 100% since some participants belonged to the other ethnic groups or did not report the information

^b^These studies were only used in stratified analyses

^c^The author provided updated analyses for all-cause mortality, cardiovascular disease mortality, and cancer mortality, so the information and data were based on the updated analyses

Supplementary Table 7 Results of the publication bias tests

| Outcomes | Classic fail-safe N | *P* value for Begg and Mazumdar rank correlation | *P* value for Egger's regression intercept | Studies trimmed | Adjusted HR (95%CI) |
| --- | --- | --- | --- | --- | --- |
| Cancer incidence | 2660 | 0.037 | 0.002 | 0 | NA |
| Cancer mortality | 9936 | 0.12 | 0.002 | 0 | NA |

*CI* confidence interval; *HR* hazard ratio; *NA* not available

Supplementary Fig. 1 Funnel plot of association between combined lifestyle factors and incident cancer


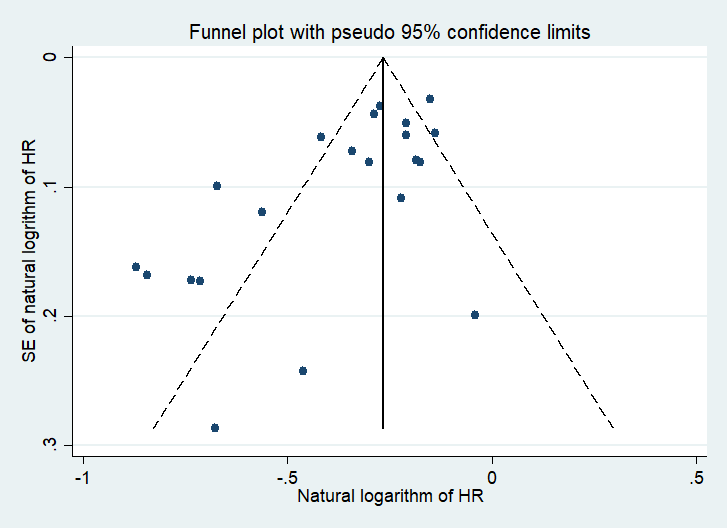


Supplementary Fig. 2 Forest plot of the healthiest lifestyle versus the least healthy lifestyle with site-specific cancer incidence


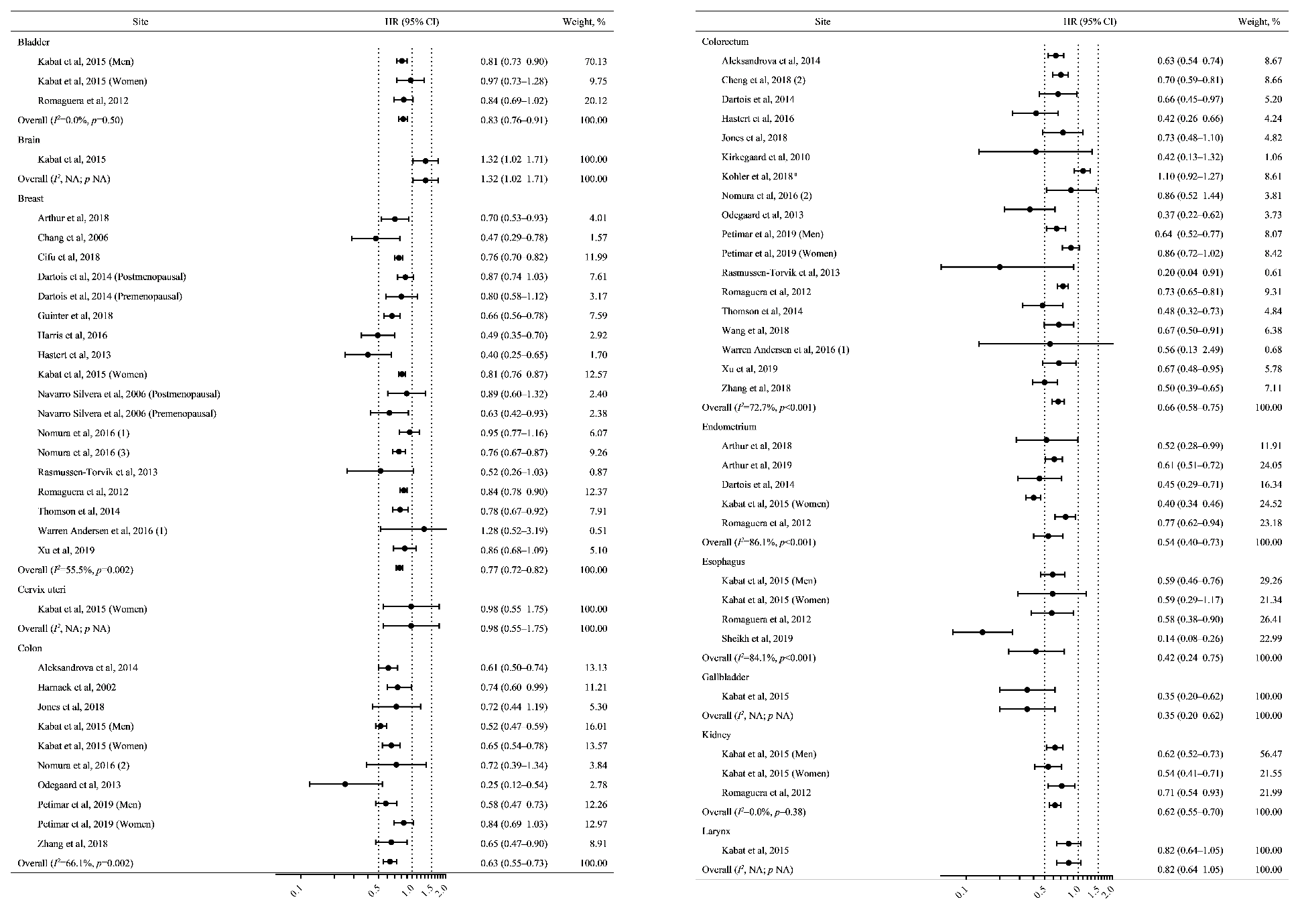


1. ^(B)^


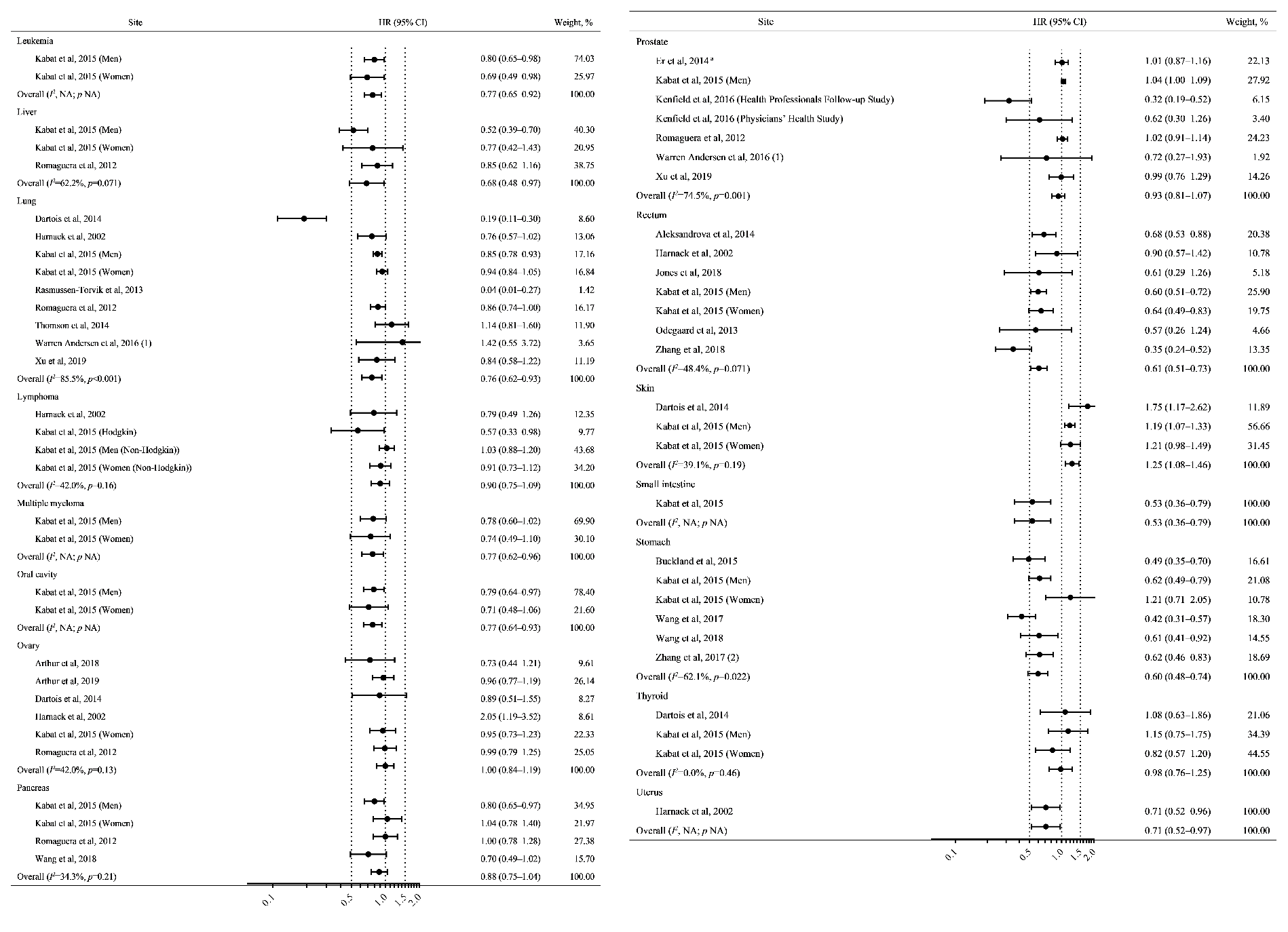


^(C) （D）^

(A) The results for bladder, brain, breast, cervix uteri, and colon cancer. (B) The results for colorectal, endometrial, esophagus, gallbladder, renal, and larynx cancer. (C) The results for leukemia, lymphoma, and multiple myeloma, as well as liver, lung, oral, ovary, and pancreas cancer. (D) The results for prostate, rectal, skin, small intestine, stomach, thyroid, and uterus cancer.

^a^Odds ratio was reported in the study and was transformed into relative risk, which was then used in the pooled analysis.

Supplementary Fig. 3 Forest plot of the healthiest lifestyle versus the least healthy lifestyle with cancer mortality among cancer survivors


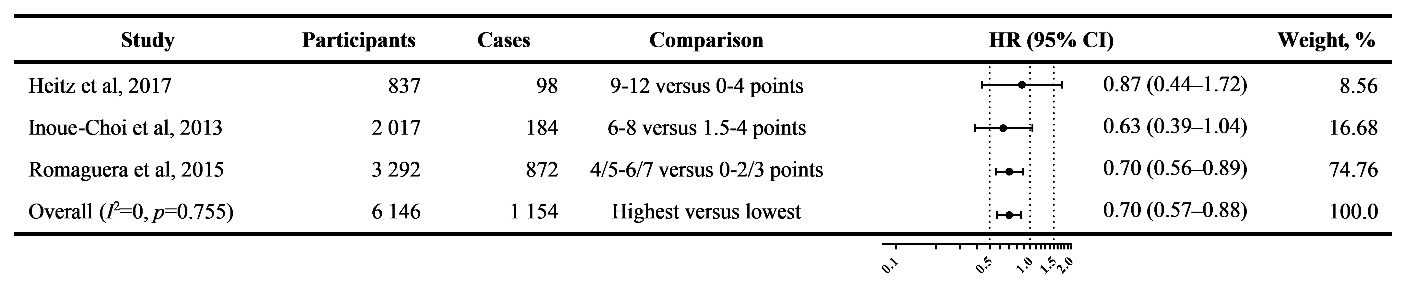


Supplementary Fig. 4 Funnel plot of association between combined lifestyle factors and cancer mortality

Supplementary Reference

1. Odegaard AO, Koh WP, Gross MD, Yuan JM, Pereira MA. Combined lifestyle factors and cardiovascular disease mortality in Chinese men and women: the Singapore Chinese health study. Circulation 2011, 124, 2847-2854

2. Greenlee H, Strizich G, Lovasi GS, Kaplan RC, Biggs ML, Li CI et al. Concordance with prevention guidelines and subsequent cancer, cardiovascular disease, and mortality: a longitudinal study of older adults. Am J Epidemiol 2017, 186, 1168-1179

3. Hastert TA, White E. Association between meeting the WCRF/AICR cancer prevention recommendations and colorectal cancer incidence: results from the VITAL cohort. Cancer Causes Control 2016, 27, 1347-1359

4. Brown K, Rumgay H, Dunlop C, Ryan M, Quartly F, Cox A et al. What proportion of cancers in the UK and its constituent countries could be prevented? An updated analysis. Journal of Global Oncology 2018, 4, 25s

5. Catsburg C, Miller AB, Rohan TE. Adherence to cancer prevention guidelines and risk of breast cancer. Int J Cancer 2014, 135, 2444-2452

6. Gu MJ, Huang QC, Bao CZ, Li YJ, Li XQ, Ye D et al. Attributable causes of colorectal cancer in China. BMC Cancer 2018, 18, 38

7. Jankovic N, Geelen A, Winkels RM, Mwungura B, Fedirko V, Jenab M et al. Adherence to the WCRF/AICR Dietary Recommendations for Cancer Prevention and Risk of Cancer in Elderly from Europe and the United States: A Meta-Analysis within the CHANCES Project. Cancer Epidemiol Biomarkers Prev 2017, 26, 136-144

8. Kulhanova I, Znaor A, Shield KD, Arnold M, Vignat J, Charafeddine M et al. Proportion of cancers attributable to major lifestyle and environmental risk factors in the Eastern Mediterranean region. Int J Cancer 2019,

9. Laaksonen MA, Arriaga M, Hull P, Canfell K, MacInnis R, Banks E et al. Burden of colorectal cancer in Australia attributable to lifestyle-related risk factors. Asia Pac J Clin Oncol 2016, 12 (Supplement 5), 73

10. Laaksonen MA, Arriaga M, Hull P, Canfell K, Macinnis R, Banks E et al. Burden of lung cancer in Australia avoidable by modifications to lifestyle-related risk factors. Cancer Research Conference: American Association for Cancer Research Annual Meeting 2017, 77,

11. Laaksonen MA, Arriaga ME, Canfell K, MacInnis RJ, Hull P, Banks E et al. Future burden of cancer attributable to current modifiable behaviours: A pooled study of seven australian cohorts. Journal of Global Oncology 2018, 4, 5s

12. Menotti A, Puddu PE, Lanti M, Maiani G, Catasta G, Fidanza AA. Lifestyle habits and mortality from all and specific causes of death: 40-year follow-up in the Italian rural areas of the seven countries study. J Nutr Health Aging 2014, 18, 314-321

13. Makarem N, Lin Y, Bandera EV, Jacques PF, Parekh N. Concordance with World Cancer Research Fund/American Institute for Cancer Research (WCRF/AICR) guidelines for cancer prevention and obesity-related cancer risk in the Framingham Offspring cohort (1991-2008). Cancer Causes Control 2015, 26, 277-286

14. Nöthlings U, Ford ES, Kroger J, Boeing H. Lifestyle factors and mortality among adults with diabetes: findings from the European Prospective Investigation into Cancer and Nutrition-Potsdam study. J Diabetes 2010, 2, 112-117

15. Pacheco-Figueiredo L, Antunes L, Bento MJ, Lunet N. Health-related behaviours in the EpiPorto study: cancer survivors versus participants with no cancer history. Eur J Cancer Prev 2011, 20, 348-354

16. Platz EA, Willett WC, Colditz GA, Rimm EB, Spiegelman D, Giovannucci E. Proportion of colon cancer risk that might be preventable in a cohort of middle-aged US men. Cancer Causes Control 2000, 11, 579-588

17. Vajdic CM, Arriaga M, Hull P, Canfell K, Macinnis R, Banks E et al. Burden of colorectal cancer attributable to lifestyle-related risk factors: a pooled study of seven Australian cohorts. Cancer Research Conference: American Association for Cancer Research Annual Meeting 2017, 77,

18. van Gemert WA, Lanting CI, Goldbohm RA, van den Brandt PA, Grooters HG, Kampman E et al. The proportion of postmenopausal breast cancer cases in the Netherlands attributable to lifestyle-related risk factors. Breast Cancer Res Treat 2015, 152, 155-162

19. Yokoyama A, Katada C, Yokoyama T, Yano T, Kaneko K, Oda I et al. Alcohol abstinence and risk assessment for second esophageal cancer in Japanese men after mucosectomy for early esophageal cancer. PLoS One 2017, 12, e0175182

20. Yun YH, Lim MK, Won YJ, Park SM, Chang YJ, Oh SW et al. Dietary preference, physical activity, and cancer risk in men: national health insurance corporation study. BMC Cancer 2008, 8, 366

21. Assi N, Gunter MJ, Thomas DC, Leitzmann M, Stepien M, Chajes V et al. Metabolic signature of healthy lifestyle and its relation with risk of hepatocellular carcinoma in a large European cohort. Am J Clin Nutr 2018, 108, 117-126

22. Romaguera D, Vergnaud AC, Peeters PH, van Gils CH, Chan DS, Ferrari P et al. Is concordance with World Cancer Research Fund/American Institute for Cancer Research guidelines for cancer prevention related to subsequent risk of cancer? Results from the EPIC study. Am J Clin Nutr 2012, 96, 150-163

23. Assi N, Thomas DC, Leitzmann M, Stepien M, Chajes V, Philip T et al. Are metabolic signatures mediating the relationship between lifestyle factors and hepatocellular carcinoma risk? Results from a nested case-control study in EPIC. Cancer Epidemiol Biomarkers Prev 2018, 27, 531-540

24. Charvat H, Sasazuki S, Inoue M, Iwasaki M, Sawada N, Shimazu T et al. Impact of five modifiable lifestyle habits on the probability of cancer occurrence in a Japanese population-based cohort: rdartoiesults from the JPHC study. Prev Med 2013, 57, 685-689

25. Sasazuki S, Inoue M, Iwasaki M, Sawada N, Shimazu T, Yamaji T et al. Combined impact of five lifestyle factors and subsequent risk of cancer: the Japan Public Health Center Study. Prev Med 2012, 54, 112-116

26. Erdrich J, Zhang X, Giovannucci E, Willett W. Proportion of colon cancer attributable to lifestyle in a cohort of US women. Cancer Causes Control 2015, 26, 1271-1279

27. Petimar J, Smith-Warner SA, Rosner BA, Chan AT, Giovannucci EL, Tabung FK. Adherence to the World Cancer Research Fund/American Institute for Cancer Research 2018 recommendations for cancer prevention and risk of colorectal cancer. Cancer Epidemiol Biomarkers Prev 2019, cebp.0165.2019

28. Gramling R, Lash TL, Rothman KJ, Cabral HJ, Silliman R, Roberts M et al. Family history of later-onset breast cancer, breast healthy behavior and invasive breast cancer among postmenopausal women: a cohort study. Breast Cancer Res 2010, 12, R82

29. Thomson CA, Van Horn L, Caan BJ, Aragaki AK, Chlebowski RT, Manson JE et al. Cancer incidence and mortality during the intervention and postintervention periods of the women's health initiative dietary modification trial. Cancer Epidemiol Biomarkers Prev 2014, 23, 2924-2935

30. Jiao L, Mitrou PN, Reedy J, Graubard BI, Hollenbeck AR, Schatzkin A et al. A combined healthy lifestyle score and risk of pancreatic cancer in a large cohort Study. Arch Intern Med 2009, 169, 764-770

31. Kabat GC, Matthews CE, Kamensky V, Hollenbeck AR, Rohan TE. Adherence to cancer prevention guidelines and cancer incidence, cancer mortality, and total mortality: a prospective cohort study. Am J Clin Nutr 2015, 101, 558-569

32. McKenzie F, Ferrari P, Freisling H, Chajes V, Rinaldi S, De Batlle J et al. Healthy lifestyle and risk of breast cancer among postmenopausal women in the European Prospective Investigation into Cancer and Nutrition cohort study. Int J Cancer 2015, 136, 2640-2648

33. Veronese N, Li Y, Manson JE, Willett WC, Fontana L, Hu FB. Combined associations of body weight and lifestyle factors with all cause and cause specific mortality in men and women: prospective cohort study. BMJ 2016, 355, i5855

34. Van Dam RM, Li T, Spiegelman D, Franco OH, Hu FB. Combined impact of lifestyle factors on mortality: prospective cohort study in US women. BMJ 2008, 337, 742-745

35. Li Y, Pan A, Wang DD, Liu X, Dhana K, Franco OH et al. Impact of healthy lifestyle factors on life expectancies in the US population. Circulation 2018, 138, 345-355

36. Atkins JL, Delgado J, Pilling LC, Bowman K, Masoli JAH, Kuchel GA et al. Impact of low cardiovascular risk profiles on geriatric outcomes: evidence from 421,000 participants in two cohorts. J Gerontol A Biol Sci Med Sci 2018,

37. Cerhan JR, Potter JD, Gilmore JM, Janney CA, Kushi LH, Lazovich D et al. Adherence to the AICR cancer prevention recommendations and subsequent morbidity and mortality in the Iowa Women's Health Study cohort. Cancer Epidemiol Biomarkers Prev 2004, 13, 1114-1120

38. Dartois L, Fagherazzi G, Boutron-Ruault MC, Mesrine S, Clavel-Chapelon F. Association between five lifestyle habits and cancer risk: results from the E3N cohort. Cancer Prev Res (Phila) 2014, 7, 516-525

39. Foraker RE, Abdel-Rasoul M, Kuller LH, Jackson RD, Van Horn L, Seguin RA et al. Cardiovascular health and incident cardiovascular disease and cancer: the Women's Health Initiative. Am J Prev Med 2016, 50, 236-240

40. Ford ES, Bergmann MM, Kroger J, Schienkiewitz A, Weikert C, Boeing H. Healthy living is the best revenge: findings from the European Prospective Investigation Into Cancer and Nutrition-Potsdam study. Arch Intern Med 2009, 169, 1355-1362

41. Harnack L, Nicodemus K, Jacobs DR, Jr., Folsom AR. An evaluation of the Dietary Guidelines for Americans in relation to cancer occurrence. Am J Clin Nutr 2002, 76, 889-896

42. Lingfors H, Persson LG. All-cause mortality among young men 24-26 years after a lifestyle health dialogue in a Swedish primary care setting: a longitudinal follow-up register study. BMJ open 2019, 9, e022474

43. McKenzie F, Biessy C, Ferrari P, Freisling H, Rinaldi S, Chajes V et al. Healthy lifestyle and risk of cancer in the European Prospective Investigation into Cancer and Nutrition cohort study. Medicine (Baltimore) 2016, 95, e2850

44. Meng L, Maskarinec G, Lee J, Kolonel LN. Lifestyle factors and chronic diseases: application of a composite risk index. Prev Med 1999, 29, 296-304

45. Ogunmoroti O, Allen NB, Cushman M, Michos ED, Rundek T, Rana JS et al. Association between Life's Simple 7 and noncardiovascular disease: the Multi-Ethnic Study of Atherosclerosis. J Am Heart Assoc 2016, 5,

46. Rasmussen-Torvik LJ, Shay CM, Abramson JG, Friedrich CA, Nettleton JA, Prizment AE et al. Ideal cardiovascular health is inversely associated with incident cancer the atherosclerosis risk in communities study. Circulation 2013, 127, 1270-1275

47. Struijk EA, May AM, Beulens JW, Fransen HP, de Wit GA, Boer JM et al. Adherence to the Dutch Guidelines for a Healthy Diet and cancer risk in the European Prospective Investigation into Cancer and Nutrition-Netherlands (EPIC-NL) cohort. Public Health Nutr 2014, 17, 2546-2553

48. Tang RQ, Zheng W, Li HL, Hu XO, Xiang YB. [Lifestyle-related factors and cancer incidence in men: a public health assessment based on a 'healthy lifestyle score']. Zhonghua Liu Xing Bing Xue Za Zhi 2013, 34, 109-113

49. Wang X, Yang X, Li J, Liu F, Chen J, Liu X et al. Impact of healthy lifestyles on cancer risk in the Chinese population. Cancer 2019,

50. Warren Andersen S, Blot WJ, Shu XO, Sonderman JS, Steinwandel MD, Hargreaves MK et al. Adherence to cancer prevention guidelines and cancer risk in low-income and African American populations. Cancer Epidemiol Biomarkers Prev 2016, 25, 846-853

51. Xu JY, Vena JE, Whelan HK, Robson PJ. Impact of adherence to cancer-specific prevention recommendations on subsequent risk of cancer in participants in Alberta's Tomorrow Project. Public Health Nutr 2019, 22, 235-245

52. Aleksandrova K, Pischon T, Jenab M, Bueno-de-Mesquita HB, Fedirko V, Norat T et al. Combined impact of healthy lifestyle factors on colorectal cancer: a large European cohort study. BMC Med 2014, 12, 168

53. Artero EG, España-Romero V, Lee DC, Sui X, Church TS, Lavie CJ et al. Ideal cardiovascular health and mortality: Aerobics Center Longitudinal Study. Mayo Clin Proc 2012, 87, 944-952

54. Arthur R, Kirsh VA, Kreiger N, Rohan T. A healthy lifestyle index and its association with risk of breast, endometrial, and ovarian cancer among Canadian women. Cancer Causes Control 2018, 29, 485-493

55. Arthur R, Brasky TM, Crane TE, Felix AS, Kaunitz AM, Shadyab AH et al. Associations of a Healthy Lifestyle Index With the Risks of Endometrial and Ovarian Cancer Among Women in the Women's Health Initiative Study. Am J Epidemiol 2019, 188, 261-273

56. Bonaccio M, Di Castelnuovo A, Costanzo S, De Curtis A, Persichillo M, Cerletti C et al. Impact of combined healthy lifestyle factors on survival in an adult general population and in high-risk groups: prospective results from the Moli-sani Study. J Intern Med 2019, 286, 207-220

57. Breslow L, Enstrom JE. Persistence of health habits and their relationship to mortality. Prev Med 1980, 9, 469-483

58. Buckland G, Travier N, Huerta JM, Bueno-De-Mesquita HB, Siersema PD, Skeie G et al. Healthy lifestyle index and risk of gastric adenocarcinoma in the EPIC cohort study. Int J Cancer 2015, 137, 598-606

59. Cheng E, Um CY, Prizment A, Lazovich D, Bostick RM. Associations of evolutionary-concordance diet, Mediterranean diet and evolutionary-concordance lifestyle pattern scores with all-cause and cause-specific mortality. Br J Nutr 2018, 1-10

60. Cheng E, Um CY, Prizment AE, Lazovich D, Bostick RM. Evolutionary-Concordance Lifestyle and Diet and Mediterranean Diet Pattern Scores and Risk of Incident Colorectal Cancer in Iowa Women. Cancer Epidemiol Biomarkers Prev 2018, 27, 1195-1202

61. Chang SC, Ziegler RG, Dunn B, Stolzenberg-Solomon R, Lacey Jr JV, Huang WY et al. Association of energy intake and energy balance with postmenopausal breast cancer in the prostate, lung, colorectal, and ovarian cancer screening trial. Cancer Epidemiol Biomarkers Prev 2006, 15, 334-341

62. Cifu G, Arem H. Adherence to lifestyle-related cancer prevention guidelines and breast cancer incidence and mortality. Ann Epidemiol 2018, 28, 767-773.e761

63. Eguchi E, Iso H, Honjo K, Yatsuya H, Tamakoshi A. No modifying effect of education level on the association between lifestyle behaviors and cardiovascular mortality: the Japan Collaborative Cohort Study. Sci Rep 2017, 7, 39820

64. Er V, Lane JA, Martin RM, Emmett P, Gilbert R, Avery KN et al. Adherence to dietary and lifestyle recommendations and prostate cancer risk in the prostate testing for cancer and treatment (ProtecT) trial. Cancer Epidemiol Biomarkers Prev 2014, 23, 2066-2077

65. Fazel-Tabar Malekshah A, Zaroudi M, Etemadi A, Islami F, Sepanlou S, Sharafkhah M et al. The combined effects of healthy lifestyle behaviors on all-cause mortality: the Golestan Cohort Study. Arch Iran Med 2016, 19, 752-761

66. Ford ES, Zhao G, Tsai J, Li C. Low-risk lifestyle behaviors and all-cause mortality: findings from the National Health and Nutrition Examination Survey III Mortality Study. Am J Public Health 2011, 101, 1922-1929

67. Ford ES, Bergmann MM, Boeing H, Li C, Capewell S. Healthy lifestyle behaviors and all-cause mortality among adults in the United States. Prev Med 2012, 55, 23-27

68. Gopinath B, Flood VM, Burlutsky G, Mitchell P. Combined influence of health behaviors on total and cause-specific mortality. Arch Intern Med 2010, 170, 1605-1607

69. Guinter MA, McLain AC, Merchant AT, Sandler DP, Steck SE. An estrogen-related lifestyle score is associated with risk of postmenopausal breast cancer in the PLCO cohort. Breast Cancer Res Treat 2018, 170, 613-622

70. Hamer M, Bates CJ, Mishra GD. Multiple health behaviors and mortality risk in older adults. J Am Geriatr Soc 2011, 59, 370-372

71. Harris HR, Bergkvist L, Wolk A. Adherence to the World Cancer Research Fund/American Institute for Cancer Research recommendations and breast cancer risk. Int J Cancer 2016, 138, 2657-2664

72. Hastert TA, Beresford SAA, Patterson RE, Kristal AR, White E. Adherence to WCRF/AICR cancer prevention recommendations and risk of postmenopausal breast cancer. Cancer Epidemiol Biomarkers Prev 2013, 22, 1498-1508

73. Hastert TA, Beresford SAA, Sheppard L, White E. Adherence to the WCRF/AICR cancer prevention recommendations and cancer-specific mortality: results from the Vitamins and Lifestyle (VITAL) Study. Cancer Causes Control 2014, 25, 541-552

74. Heitz AE, Baumgartner RN, Baumgartner KB, Boone SD. Healthy lifestyle impact on breast cancer-specific and all-cause mortality. Breast Cancer Res Treat 2018, 167, 171-181

75. Inoue-Choi M, Robien K, Lazovich D. Adherence to the WCRF/AICR guidelines for cancer prevention is associated with lower mortality among older female cancer survivors. Cancer Epidemiol Biomarkers Prev 2013, 22, 792-802

76. Jones P, Cade JE, Evans CEL, Hancock N, Greenwood DC. Does adherence to the World Cancer Research Fund/American Institute of Cancer Research cancer prevention guidelines reduce risk of colorectal cancer in the UK Women's Cohort Study? Br J Nutr 2018, 119, 340-348

77. Kenfield SA, Batista JL, Jahn JL, Downer MK, Van Blarigan EL, Sesso HD et al. Development and application of a lifestyle score for prevention of lethal prostate cancer. J Natl Cancer Inst 2016, 108, djv329

78. Khaw KT, Wareham N, Bingham S, Welch A, Luben R, Day N. Combined impact of health behaviours and mortality in men and women: the EPIC-Norfolk prospective population study. PLoS Med 2008, 5, 0039-0047

79. Kirkegaard H, Johnsen NF, Christensen J, Frederiksen K, Overvad K, Tjonneland A. Association of adherence to lifestyle recommendations and risk of colorectal cancer: a prospective Danish cohort study. BMJ 2010, 341, 978

80. Kohler LN, Harris RB, Oren E, Roe DJ, Lance P, Jacobs ET. Adherence to Nutrition and Physical Activity Cancer Prevention Guidelines and Development of Colorectal Adenoma. Nutrients 2018, 10,

81. Knoops KT, de Groot LC, Kromhout D, Perrin AE, Moreiras-Varela O, Menotti A et al. Mediterranean diet, lifestyle factors, and 10-year mortality in elderly European men and women: the HALE project. JAMA 2004, 292, 1433-1439

82. Kvaavik E, Batty GD, Ursin G, Huxley R, Gale CR. Influence of individual and combined health behaviors on total and cause-specific mortality in men and women: The United Kingdom Health and Lifestyle Survey. Arch Intern Med 2010, 170, 711-718

83. Lee CD, Sui X, Hooker SP, Hebert JR, Blair SN. Combined impact of lifestyle factors on cancer mortality in men. Ann Epidemiol 2011, 21, 749-754

84. Lin CC, Li CI, Liu CS, Lin WY, Fuh MM, Yang SY et al. Impact of lifestyle-related factors on all-cause and cause-specific mortality in patients with type 2 diabetes: the Taichung Diabetes Study. Diabetes Care 2012, 35, 105-112

85. Lohse T, Faeh D, Bopp M, Rohrmann S. Adherence to the cancer prevention recommendations of the World Cancer Research Fund/American Institute for Cancer Research and mortality: a census-linked cohort. Am J Clin Nutr 2016, 104, 678-685

86. Martin-Diener E, Meyer J, Braun J, Tarnutzer S, Faeh D, Rohrmann S et al. The combined effect on survival of four main behavioural risk factors for non-communicable diseases. Prev Med 2014, 65, 148-152

87. McCullough ML, Patel AV, Kushi LH, Patel R, Willett WC, Doyle C et al. Following cancer prevention guidelines reduces risk of cancer, cardiovascular disease, and all-cause mortality. Cancer Epidemiol Biomarkers Prev 2011, 20, 1089-1097

88. Navarro Silvera SA, Jain M, Howe GR, Miller AB, Rohan TE. Energy balance and breast cancer risk: a prospective cohort study. Breast Cancer Res Treat 2006, 97, 97-106

89. Nechuta SJ, Shu XO, Li HL, Yang G, Xiang YB, Cai H et al. Combined impact of lifestyle-related factors on total and cause-specific mortality among Chinese women: prospective cohort study. PLoS Med 2010, 7, e1000339

90. Nomura SJ, Dash C, Rosenberg L, Yu J, Palmer JR, Adams-Campbell LL. Adherence to diet, physical activity and body weight recommendations and breast cancer incidence in the Black Women's Health Study. Int J Cancer 2016, 139, 2738-2752

91. Nomura SJ, Dash C, Rosenberg L, Yu J, Palmer JR, Adams-Campbell LL. Is adherence to diet, physical activity, and body weight cancer prevention recommendations associated with colorectal cancer incidence in African American women? Cancer Causes Control 2016, 27, 869-879

92. Nomura SJ, Inoue-Choi M, Lazovich D, Robien K. WCRF/AICR recommendation adherence and breast cancer incidence among postmenopausal women with and without non-modifiable risk factors. Int J Cancer 2016, 138, 2602-2615

93. Odegaard AO, Koh WP, Yuan JM. Combined lifestyle factors and risk of incident colorectal cancer in a Chinese population. Cancer Prev Res 2013, 6, 360-367

94. Petersen KE, Johnsen NF, Olsen A, Albieri V, Olsen LK, Dragsted LO et al. The combined impact of adherence to five lifestyle factors on all-cause, cancer and cardiovascular mortality: a prospective cohort study among Danish men and women. Br J Nutr 2015, 113, 849-858

95. Romaguera D, Ward H, Wark PA, Vergnaud AC, Peeters PH, van Gils CH et al. Pre-diagnostic concordance with the WCRF/AICR guidelines and survival in European colorectal cancer patients: a cohort study. BMC Med 2015, 13, 107

96. Sheikh M, Poustchi H, Pourshams A, Etemadi A, Islami F, Khoshnia M et al. Individual and Combined Effects of Environmental Risk Factors for Esophageal Cancer Based on Results From the Golestan Cohort Study. Gastroenterology 2019, 156, 1416-1427

97. van Lee L, Geelen A, Kiefte-de Jong JC, Witteman JC, Hofman A, Vonk N et al. Adherence to the Dutch dietary guidelines is inversely associated with 20-year mortality in a large prospective cohort study. Eur J Clin Nutr 2016, 70, 262-268

98. Vergnaud AC, Romaguera D, Peeters PH, van Gils CH, Chan DS, Romieu I et al. Adherence to the World Cancer Research Fund/American Institute for Cancer Research guidelines and risk of death in Europe: results from the European Prospective Investigation into Nutrition and Cancer cohort study. Am J Clin Nutr 2013, 97, 1107-1120

99. Wang Z, Koh WP, Jin A, Wang R, Yuan JM. Composite protective lifestyle factors and risk of developing gastric adenocarcinoma: the Singapore Chinese Health Study. Br J Cancer 2017, 116, 679-687

100. Wang W, Fung TT, Wang M, Smith-Warner SA, Giovannucci EL, Tabung FK. Association of the Insulinemic Potential of Diet and Lifestyle With Risk of Digestive System Cancers in Men and Women. JNCI cancer spectrum 2018, 2, pky080

101. Warren Andersen S, Zheng W, Sonderman J, Shu XO, Matthews CE, Yu D et al. Combined impact of health behaviors on mortality in low-income Americans. Am J Prev Med 2016, 51, 344-355

102. Wingard DL, Berkman LF, Brand RJ. A multivariate analysis of health-related practices: a nine-year mortality follow-up of the Alameda County Study. Am J Epidemiol 1982, 116, 765-775

103. Yun JE, Won S, Kimm H, Jee SH. Effects of a combined lifestyle score on 10-year mortality in Korean men and women: a prospective cohort study. BMC Public Health 2012, 12, 673

104. Zhang QL, Zhao LG, Zhang W, Li HL, Gao J, Han LH et al. Combined impact of known lifestyle factors on total and cause-specific mortality among Chinese men: a prospective cohort study. Sci Rep 2017, 7, 5293

105. Zhang QL, Zheng W, Li HL, Gao J, Fang J, Gao LF et al. [The joint effects of major lifestyle factors on stomach cancer risk among Chinese men: a prospective cohort study]. Zhonghua Yu Fang Yi Xue Za Zhi 2017, 51, 386-392

106. Zhang QL, Zhao LG, Li HL, Gao J, Yang G, Wang J et al. The joint effects of major lifestyle factors on colorectal cancer risk among Chinese men: a prospective cohort study. Int J Cancer 2018, 142, 1093-1101
